# Synthesis of 1,2,4-Oxadiazin-5(6*H*)-One Derivatives and Their Biological Investigation as Monoamine Oxidase Inhibitors

**DOI:** 10.3390/molecules29235550

**Published:** 2024-11-25

**Authors:** Sofia I. Presnukhina, Valentina D. Kotlyarova, Anton A. Shetnev, Sergey V. Baykov, Rakhymzhan Turmanov, Nurbol Appazov, Rakhmetulla Zhapparbergenov, Leilya Zhussupova, Nurila Togyzbayeva, Stephanus J. Cloete, Mikhail K. Korsakov, Vadim P. Boyarskiy, Anél Petzer, Jacobus P. Petzer

**Affiliations:** 1 Institute of Chemistry, Saint Petersburg State University, Universitetskaya Nab., 7/9, 199034 Saint Petersburg, Russia; sonya.presnuxina.98@mail.ru (S.I.P.); sergei.v.baikov@yandex.ru (S.V.B.); v.boiarskii@spbu.ru (V.P.B.); 2 Pharmaceutical Technology Transfer Centre, Yaroslavl State Pedagogical University Named after K.D. Ushinsky, Respublikanskaya St., 108, 150000 Yaroslavl, Russia; kvd35@mail.ru (V.D.K.); mkkors@mail.ru (M.K.K.); 3 Laboratory of Engineering Profile “Physical and Chemical Methods of Analysis”, Korkyt Ata Kyzylorda University, 29 Aiteke Bi Str., Kyzylorda 120014, Kazakhstan; a.shetnev@yspu.org (A.A.S.); nurasar.82@korkyt.kz (N.A.); ulagat-91@mail.ru (R.Z.); laila.zhusupova@mail.ru (L.Z.); nurila2009@mail.ru (N.T.); 4 Moscow Center for Advanced Studies, 20, Kulakova Str., 123592 Moscow, Russia; 5 “DPS Kyzylorda” LLP, Amangeldy Imanov Str., 112A, Kyzylorda 120008, Kazakhstan; 6 “CNEC” LLP, Dariger Ali Lane, 2, Kyzylorda 120001, Kazakhstan; 7 Pharmaceutical Chemistry and Centre of Excellence for Pharmaceutical Sciences, North-West University, Potchefstroom 2520, South Africa; 23496959@mynwu.ac.za (S.J.C.); anel.petzer@nwu.ac.za (A.P.)

**Keywords:** monoamine oxidase, MAO, oxadiazine, inhibition, Parkinson’s disease

## Abstract

Monoamine oxidase (MAO) plays a key role in the pathogenesis of central nervous system disorders, and MAO inhibitors have been used in the treatment of depression and Parkinson’s disease. In the search for new classes of MAO inhibitors, the present study investigated a series of 1,2,4-oxadiazin-5(6*H*)-one derivatives. This study provides the first optimization of the reaction conditions for the condensation of amidoximes with alkyl 2-halocarboxylates to yield the desired 1,2,4-oxadiazin-5(6*H*)-ones. The results of the in vitro MAO inhibition studies showed that the 1,2,4-oxadiazin-5(6*H*)-ones were indeed inhibitors of human MAO with the most potent inhibition observed for **5f** (IC_50_ = 0.900 µM) and **7c** (IC_50_ = 0.371 µM). It was concluded that, with appropriate substitution, 1,2,4-oxadiazin-5(6*H*)-one derivatives would act as good potency MAO-B inhibitors and lead compounds for the development of antiparkinsonian drugs. In Parkinson’s disease, MAO-B inhibitors enhance central dopamine levels and reduce MAO-mediated production of hydrogen peroxide and resultant oxidative injury. This study represents one of few works to investigate synthetic approaches and biological activities of the 1,2,4-oxadiazin-5(6*H*)-one class of heterocycles.

## 1. Introduction

Monoamine oxidase (MAO, EC 1.4.3.4) is an intracellular, mitochondrial enzyme that contains a covalently bound flavin adenine dinucleotide (FAD) cofactor [1,2]. MAO is expressed in the brain as well as peripheral tissues where it has a key function in the metabolism of neurotransmitter amines while also acting as a metabolic barrier to bioactive amine compounds that are derived from the diet [2]. In mammals, two isoforms of MAO are expressed, namely, MAO-A and MAO-B, which are products of distinct genes [3]. Although the MAO isoforms share approximately 70% amino acid sequence identity and have similar three-dimensional structures and active sites, these isoforms display different substrate and inhibitor specificities [4,5]. MAO-A specifically metabolizes serotonin, while the arylalkylamine compounds, benzylamine and phenylethylamine, are specific substrates for MAO-B. Dopamine, noradrenaline, adrenaline, and tyramine are examples of compounds that are metabolized by both isoforms [6]. MAO catalyzes the α-carbon oxidation of these amine substrates to yield the corresponding aldehyde, ammonia (from primary amines) and hydrogen peroxide, which is generated upon reoxidation of the FAD by molecular oxygen. The by-products of MAO catalysis, most notably hydrogen peroxide, have been implicated in the pathogenesis of certain diseases [7]. In Parkinson’s disease, hydrogen peroxide generated by MAO in the brain may, under certain conditions, contribute to oxidative damage and neurodegeneration. Hydrogen peroxide may be converted in the Fenton reaction to the highly reactive hydroxyl radical, which reacts with lipids, proteins and DNA and contributes to neurodegeneration in Parkinson’s disease. This is further aggravated by the fact that MAO-B expression in neuronal tissues increases with aging [8]. In heart tissue, MAO-A has a similar role where MAO-A-generated hydrogen peroxide and resulting oxidative stress may lead to mitochondrial and cardiac dysfunction [9,10]. Based on the role of MAO-A as a source of oxidative stress in the heart, MAO-A inhibitors may represent a future strategy for the treatment of heart disease. Interestingly, increased expression of MAO-A has been linked to prostate cancer, and the pharmacological inhibition of MAO-A has been shown to reduce or even eliminate prostate tumor growth and metastasis in animal models [11,12].

Based on their physiological roles in the metabolism of neurotransmitters, the MAOs are validated drug targets and inhibitors of these enzymes have been used to treat disorders such as Parkinson’s disease and depression [13]. Besides a potential neuroprotective role in Parkinson’s disease, MAO-B inhibitors also reduce central dopamine metabolism and depletion and thereby provide a symptomatic benefit, particularly as an adjuvant to levodopa therapy [14]. Although both MAO isoforms metabolize dopamine, MAO-B inhibitors are used in Parkinson’s disease due to safety concerns over early irreversible MAO-A inhibitors, while with aging, the MAO-B isoform increases in the brain, suggesting a more dominant role in dopamine metabolism for MAO-B versus MAO-A. In depression, MAO-A inhibitors reduce the central metabolism of serotonin and noradrenaline, while enhanced MAO-A density in several brain regions has been implicated in the depletion of monoamine levels in major depression [15]. Among the classical neurotransmitters that are metabolized by the MAOs, serotonin and the deficiency thereof has been most closely linked to depression, which underscores a role of MAO-A inhibitors in the treatment of this illness. MAO inhibitor drugs that have been approved for clinical use include irreversible (e.g., tranylcypromine, phenelzine, rasagiline, selegiline) and reversible (e.g., moclobemide, safinamide) inhibitors [6].

**Figure 1:**
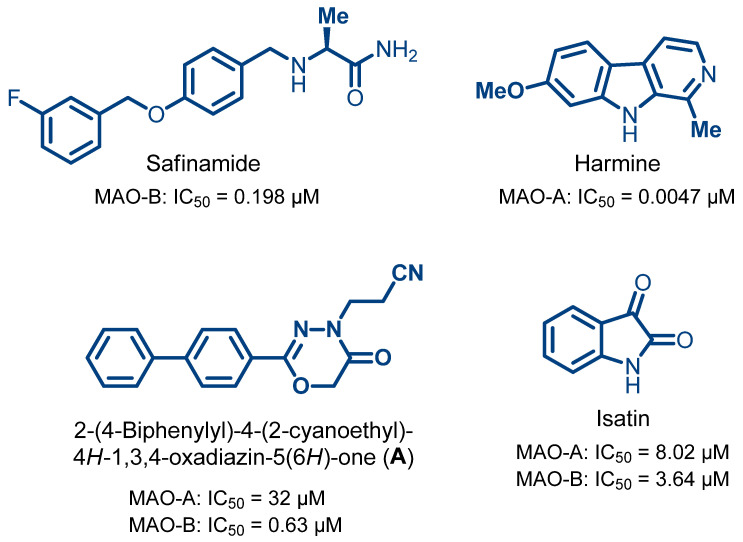
The structures of selected MAO inhibitors.

Numerous heterocyclic compounds have been reported as possessing MAO inhibition activities. Examples are the nonspecific inhibitor, isatin, and the MAO-A-specific inhibitor, harmine (Figure 1) [5,16]. Heterocyclic moieties possess the required polar groups that can act as hydrogen bond donors and acceptors for residues and water molecules in the MAO active site. In general, both MAO isoforms have nonpolar active sites; however, regions where polar interactions are possible exist in proximity to the FAD, which is where the heterocyclic moieties of many MAO inhibitors bind [17]. In the search for new classes of heterocyclic compounds that inhibit the MAO isoform, the present study investigates a series of 1,2,4-oxadiazin-5(6*H*)-ones as potential MAO inhibitors. Isomeric 1,3,4-oxadiazin-5(6*H*)-one derivatives (e.g., compound **A**) have been reported to inhibit MAO [18]. These compounds have the potential to form hydrogen bonds, while cyclic amidine structures have been shown to possess a diversity of biological activities [19,20,21,22,23]. In this work, we investigated the 1,2,4-oxadiazine heterocycles, which belong to the class of cyclic amidines. Several studies to date have investigated synthetic approaches and biological activities of this class of compounds [24,25,26,27]. Particularly, in our investigation of the transformation of amidoximes to different aza-heterocycles in base/DMSO medium [28], we reported the formation of the 1,2,4-oxadiazin-5(6*H*)-one moiety by the reaction of amidoximes with dimethyl maleate [29]. The obtained 1,2,4-oxadiazin-5(6*H*)-ones were functionalized on the C6 position with a carboxylic acid group or its derivatives (methoxy carbonyl group or 1,2,4-oxadiazole ring). Methyl chloroacetate was also evaluated as a reagent for the preparation of the 1,2,4-oxadiazin-5(6*H*)-one ring via condensation with *p*-tolyl amidoxime [30]; however, the product yield was extremely low (13%). Thus, this study had two goals: first, to optimize the conditions of the reaction between 2-halocarboxylates and amidoximes, and second, to evaluate all 1,2,4-oxadiazin-5(6*H*)-ones (new as well as previously obtained) as MAO inhibitors.

## 2. Results and Discussion

### 2.1. Chemistry

This study set out to first optimize the reaction conditions for the condensation of *p*-tolyl amidoxime (**1a**) and methyl 2-haloacetates (**2a**,**b**), which served as model substrates (Table 1). Carbonyl compounds **2**, bases, reagent amounts, solvents, and time were varied; in all cases, the reactions were carried out at room temperature. First, methyl 2-bromoacetate and NaOH (Table 1, entries 1–3) were used in the reaction. Initially, methyl 2-bromoacetate/NaOH at a 1:1 ratio (Table 1, entry 1) and a 1:1.25 ratio (Table 1, entry 2) was tested with a reaction time of 4 h. In both cases, uncycled intermediate ***IP*** was isolated as the only product. By increasing the base amount and extending the reaction time, target product **3a** was obtained in a yield of 29% (Table 1, entry 3). To increase the product yield, *t*-BuONa was used as a stronger base (Table 1, entries 4–6) with the conditions being similar to entries 1–3. When methyl 2-bromoacetate/*t*-BuONa were used in a 1:1 ratio, the reaction afforded only intermediate ***IP*** (Table 1, entry 4). Interestingly, if the 2-bromoacetate/*t*-BuONa ratio was 1:1.25 for 4 h (Table 1, entry 5), only the by-product ***BP***, which is the result of N-alkylation of the target oxadiazinone, was isolated from the reaction mixture. Therefore, we increased the amount of base and the reaction time. Indeed, this allowed us to increase the proportion of cyclization product **3a**, which we were able to isolate with a yield of 42% (Table 1, entry 6). Surprisingly, changing the carbonyl compound to methyl 2-chloroacetate led to improved yields (Table 1, entries 7–14). In the first trial, we used the conditions from entry 3, which has proven to be successful, and the yield of the target product was 35% (Table 1, entry 7). This yield was higher than that obtained in entry 3. The use of KOH instead of NaOH did not lead to a significant increase in product yield, which was 38% (Table 1, entry 8). However, the reaction with *t*-BuONa gave 3-(4-methylphenyl)-4*H*-1,2,4-oxadiazin-5(6*H*)-one (**3a**) in a satisfactory yield of 50% (Table 1, entry 9). In the next trial, the stronger base *t*-BuOK was used with the aim to increase the product yield, but the corresponding 1,2,4-oxadiazin-5(6*H*)-one was obtained in a lower yield of 32% (Table 1, entry 10). Further optimization showed that the substitution of DMSO for alternative aprotic solvents also was not beneficial (Table 1, entries 11 and 12). Increasing the reaction time to 48 h (Table 1, entry 13) also did not increase the product yield (45%). Finally, we carried out the reaction with 1.5 equiv. of methyl 2-chloroacetate (Table 1, entry 14) and found a yield of only 13%. Thus, entry 9 represents the most optimal conditions (e.g., base, reagent amounts, solvent, and time) when using carbonyl compound **2b** as a substrate, producing the target 1,2,4-oxadiazin-5(6*H*)-one (**3a**) with a satisfactory yield of 50%.

**Scheme 1:**
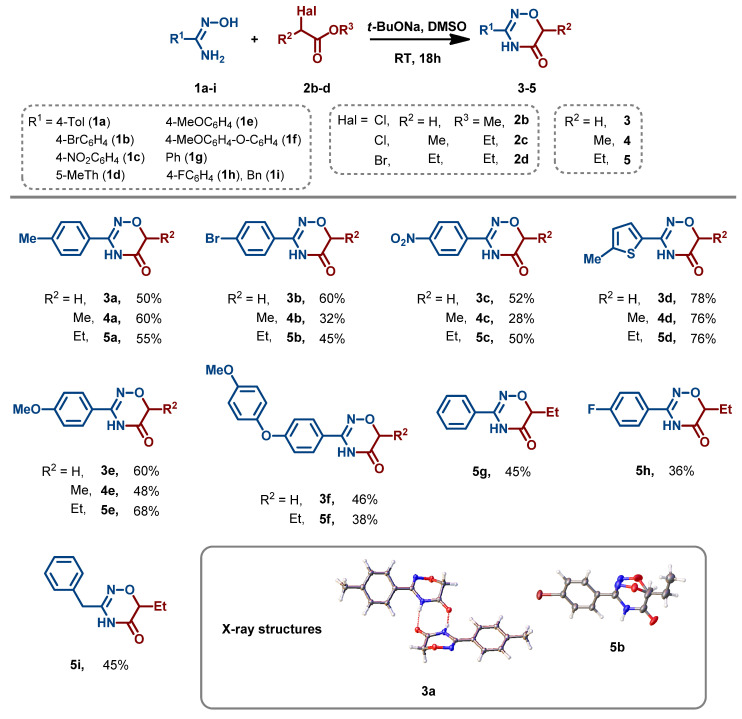
Reactions of amidoximes **1** with 2-halocarboxylate **2** in a *t*-BuONa–DMSO medium (top panel); single-crystal XRD structures of products **3a** and **5b** (side panel). Conditions: **1** (2 mmol), **2** (2.4 mmol), *t*-BuONa (4 mmol), DMSO (3 mL), RT, 18 h. Isolated yields are presented.

Using the optimized conditions, we applied the reactions of amidoximes **1a**–**i** and carbonyl compounds **2b**–**d** to synthesize the corresponding 1,2,4-oxadiazin-5(6*H*)-ones **3**–**5** (Scheme 1). All products were obtained in moderate to good yields. The method can also be used to synthesize 6-alkylsubstituted 1,2,4-oxadiazin-5(6*H*)-ones. Ethyl 2-chloropropionate and ethyl 2-bromobutyrate afford 6-methyl- and 6-ethylsubstituted 1,2,4-oxadiazin-5(6*H*)-ones, respectively. It was found that the presence of electron-withdrawing and electron-donating groups on the phenyl ring of amidoximes did not have a significant effect on the product yield. Phenylamidoxime **1g** and benzylamidoxime **1i** reacted with ethyl 2-bromobutyrate. Both gave the desired product **5** in isolated yields of 45%. The best result was found for heterocyclic amidoxime **1d**, which gave the highest yields for the condensation products (**3d** 78%; **4d** 76%; **5d** 76%). The structures of two compounds (**3a** and **5b**) were confirmed by X-ray diffraction (Scheme 1). These are rare examples of crystallographic studies of 1,2,4-oxadiazin-5(6*H*)-ones (in the Cambridge Structural Database, only six of structures bearing the 1,2,4-oxadiazin-5(6*H*)-one core were found; CSD-codes: DERYUY, DERZAF, DERZEJ, PAJQOH, WIFYAP, ZUGFIS). Also, it should be noted that for **3a**, there were two crystallographically independent molecules, which were bound by N–H⋯O hydrogen bonds, while for the structure of **5b**, a disordered 1,2,4-oxadiazin-5(6*H*)-one ring was observed.

**Scheme 2:**
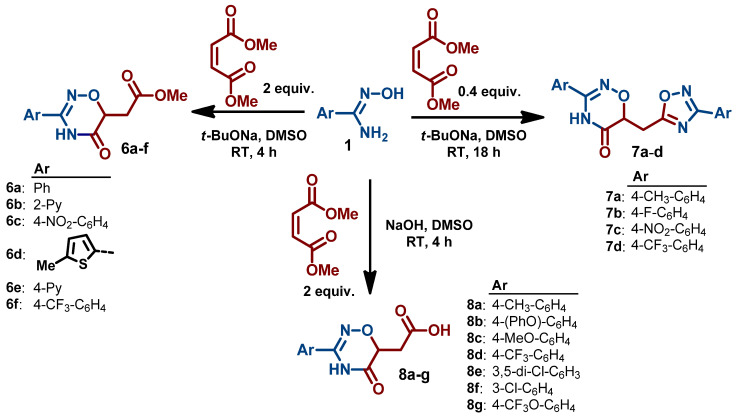
Synthesis of compounds **6**–**8** via reactions of amidoximes **1** with dimethyl maleate.

To more systematically evaluate 1,2,4-oxadiazin-5(6*H*)-one derivatives as potential MAO inhibitors, a series of 15 of our previously synthesized compounds [29], containing a methoxycarbonyl function (**6a**–**f**), an oxadiazole ring (**7a**–**d**), and a carboxyl group (**8a**–**e**) at position 6 (Scheme 2), were included in this study. Moreover, two carboxylic acids (**8f** and **8g**, Scheme 2) were also prepared and included in the MAO inhibition studies. The structures of the 1,2,4-oxadiazin-5(6*H*)-ones were characterized with NMR and mass spectrometry, while the X-ray crystal structures of **3a** and **5b** were recorded (Scheme 1). In all instances, the physical data corresponded with the proposed structures. It should be noted that C6 of the 1,2,4-oxadiazin-5(6*H*)-one derivatives (**4a**–**e**, **5a**–**i**, **6a**–**f**, **7a**–**d**, and **8a**–**g**) is a stereogenic center, yielding enantiomers for each derivative. This study did not attempt to isolate the enantiomers, and the biological data were recorded for the racemic mixtures.

### 2.2. Biological Studies

**Figure 2:**
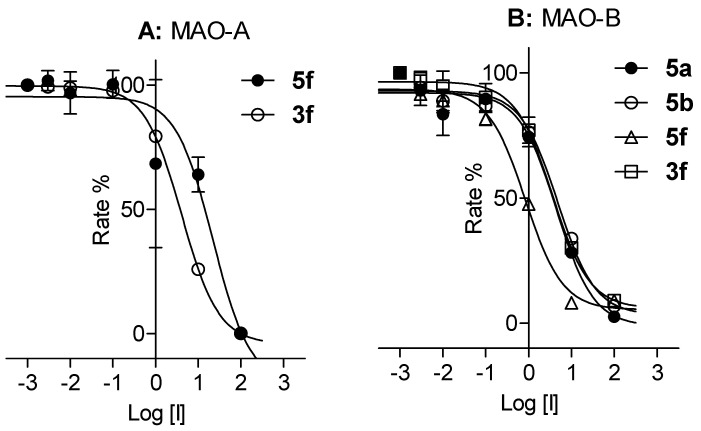
Plots of the activities of MAO-A (**A**) and MAO-B (**B**) in the presence of selected inhibitors. These plots were used to estimate IC_50_ values. Each data point represents the mean ± standard deviation (SD) of triplicate measurements.

The 1,2,4-oxadiazin-5(6*H*)-one derivatives (**3**–**8**, 37 compounds) were investigated as potential inhibitors of MAO by employing the recombinant human MAO-A and MAO-B enzymes and kynuramine as a substrate [31]. The in vitro inhibition studies were carried out according to the published procedure, and the inhibition potencies were expressed as the IC_50_ values (Figure 2) [32]. The reference inhibitors, isatin, harmine and safinamide, were selected for this study since they are, similar to the study compounds, reversible MAO inhibitors. Isatin is a nonspecific inhibitor, while harmine and safinamide represent potent and specific inhibitors of MAO-A and MAO-B, respectively. The results are presented in Table 2 and show that most 1,2,4-oxadiazin-5(6*H*)-one derivatives inhibited MAO-A and MAO-B with modest potencies (IC_50_ > 10 µM). Only seven derivatives exhibited IC_50_ < 10 µM for the inhibition of MAO-B: **3f** (IC_50_ = 3.98 µM), **5a** (IC_50_ = 4.28 µM), **5b** (IC_50_ = 5.11 µM), **5d** (IC_50_ = 6.14 µM), **5e** (IC_50_ = 6.22 µM), **5f** (IC_50_ = 0.900 µM) and **7c** (IC_50_ = 0.371 µM). While clear structure–activity relationships were not apparent, it was noticeable that five of the most potent MAO-B inhibitors (**5a**, **5b**, **5d**, **5e** and **5f**) were substituted with an ethyl on the C6 position of the 1,2,4-oxadiazin-5(6*H*)-one ring. These compounds were more potent inhibitors than the unsubstituted (**3**) and methyl (**4**) substituted homologues, which indicated that an ethyl in this position was more advantageous for MAO-B inhibition. However, not all derivatives with C6 ethyl substituents were potent MAO-B inhibitors (e.g., **5c**, **5g**, **5h**, **5i**). Other substituents on the C6 position explored in this study (e.g., substituted oxadiazoles, methyl acetate and acetic acid) resulted in moderate to poor inhibition. Two other compounds were also found to be good potency MAO-B inhibitors: **3f** and **7c**. Compound **3f** was not substituted on C6 but possessed a larger substituent on the C3 position. The good potency of this compound may have been dependent on an ability to better fill the MAO-B active site. Indeed, many good potency MAO-B inhibitors such as safinamide have larger extended structures. A variety of C3 substituents were explored in this study, which included the phenyl, benzyl and 5-methylthiophen-2-yl moieties, while different substituents on the phenyl (e.g., CH_3_, OCH_3_, Cl, Br, F, CF_3_, OCF_3_, NO_2_, PhO) were considered. Although it was not possible to highlight a specific C3 substituent as most optimal for MAO inhibition, among the seven most potent MAO-B inhibitors, two compounds were substituted with the 4-(methoxyphenoxy)phenyl on C3 (**3f** and **5f**). Compound **5f** was the second most potent MAO-B inhibitor of the study, which indicated that this group should be considered in further structure optimization studies.

Only four derivatives exhibited IC_50_ < 15 µM for the inhibition of MAO-A: **3b** (IC_50_ = 14.2 µM), **3f** (IC_50_ = 3.75 µM), **7c** (IC_50_ = 12.9 µM) and **8b** (IC_50_ = 12.7 µM). The large structural diversity of these compounds precluded the possibility to derive meaningful structure–activity relationships. It was, however, noticeable that both **3b** and **3f** were unsubstituted on the C6 position.

**Figure 3:**
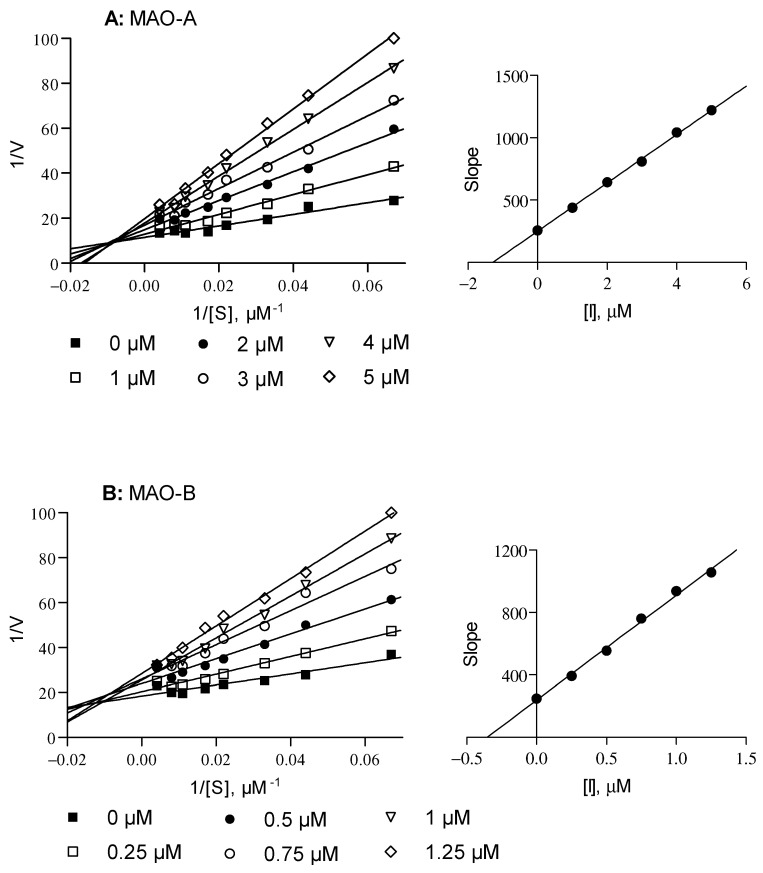
Lineweaver–Burk plots for the inhibition of MAO-A (**A**) and MAO-B (**B**) by **3f** and **5f**, respectively. The insets are replots of the slopes of the Lineweaver–Burk plots versus the concentration of the inhibitor. The legends indicate the inhibitor concentrations used.

To investigate the mode of MAO inhibition, Lineweaver–Burk plots were constructed for the two inhibitors of the respective MAO isoforms, **3f** and **5f**. For each inhibitor, six plots were constructed using a range of eight substrate concentrations (15–250 µM) for each plot. The inhibitor concentrations were selected to bracket the IC_50_ values. For the inhibition of MAO-A by **3f,** the inhibitor concentrations ranged from 1 to 5 µM, while for the inhibition of MAO-B by **5f,** the inhibitor concentrations ranged from 0.25 to 1.25 µM. For each isoform, a plot was also constructed in the absence of the inhibitor. The Lineweaver–Burk plots are presented in Figure 3 and are indicative of competitive inhibition since the lines intersect close to the y-axis, while the slopes increase with increasing inhibitor concentration. From replots of the slopes versus inhibitor concentration, K_i_ values of 1.3 and 0.35 µM were estimated for the inhibition of MAO-A and MAO-B by **3f** and **5f**, respectively.

### 2.3. Molecular Docking

**Figure 4:**
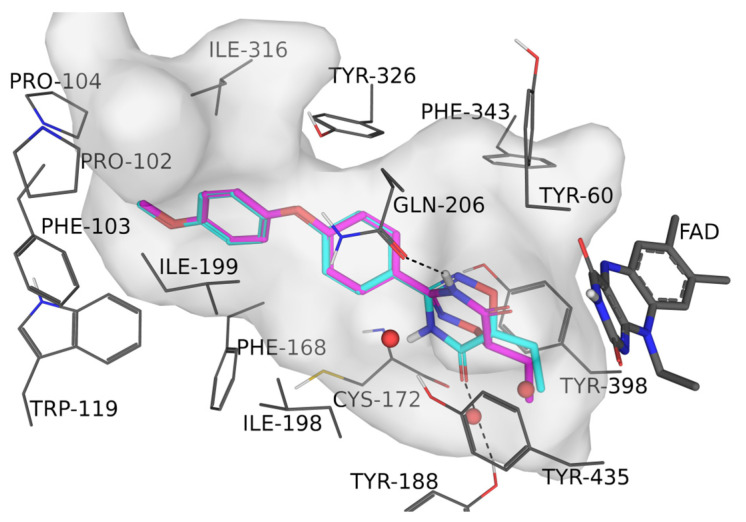
The binding orientations (R)-**5f** (cyan) and (S)-**5f** (magenta) to the active site of MAO-B. The surface indicates the active site cavity, while dashed lines indicate hydrogen bonds.

To determine the role of the 1,2,4-oxadiazin-5(6*H*)-one moiety in the binding of the derivatives to MAO-B, molecular docking experiments were carried out. Compound **5f** was selected for the docking simulations and both enantiomers were considered, while molecular docking was performed with the CDOCKER module of Discovery Studio 3.1 as previously reported [32]. The X-ray structure of human MAO-B with safinamide bound to the active site (PDB code: 2V5Z) was selected as the protein model and was prepared for the simulations as reported [32]. The results showed that both enantiomers of **5f** bound with the 1,2,4-oxadiazin-5(6*H*)-one moiety in proximity of the FA,D, while the 4-(methoxyphenoxy)phenyl substituent on C3 projected into the entrance cavity, the space beyond the gating residue, Ile199 (Figure 4). For (R)-**5f**, hydrogen bonding was observed between the carbonyl of the inhibitor and the phenol group of Tyr188, while for (S)-**5f**, hydrogen bonding was observed between the oxadiazinone NH and the side chain carbonyl of Gln206. It was noteworthy that, compared to (R)-**5f**, the 1,2,4-oxadiazin-5(6*H*)-one moiety of (S)-**5f** was rotated through approximately 150° to allow for placement of the ethyl substituents of the two enantiomers in the same space between the aromatic rings of Tyr398 and Tyr435. The placement of the ethyl side chain in this space seems to be beneficial for inhibitor stabilization when considering that the unsubstituted compound **3f** is a weaker MAO-B inhibitor than **5f**. For both enantiomers of **5f**, the phenyl substituent formed a pi–pi interaction with Tyr326 and a pi–sulfur interaction with Cys172. The C3 side chain underwent numerous van der Waals interactions with residues lining the entrance cavity (e.g., Phe103, Pro104, Trp119, Leu164, Leu171, Ile199 and Ile316).

## 3. Experimental Section

### 3.1. Material and Methods

Starting amidoximes were prepared from commercial nitriles according to the procedures in [33,34,35]. The preparation and characteriation of compounds **6a**–**f**, **7a**–**d**, **8a**–**e** were described previously [29]. All other reagents and solvents were purchased from Merck and were used without furher purification. Reactions were monitored by analytical thin-layer chromatography (TLC) with Macherey–Nagel TLC sheets (Silufol UV–254) (Macherey–Nagel GmbH & Co. KG, Düren, Germany) using UV light for detection. NMR spectra were recorded with a Bruker Avance DPX 400 (Bruker Optics GmbH & Co. KG, Ettlingen, Germany) instrument (400 MHz, 101 MHz and 376 MHz for ^1^H, ^13^C and ^19^F, respectively) in DMSO-*d*_6_ or in CDCl_3_. Chemical shifts are reported as parts per million (*δ*, ppm); the solvent peaks were used as internal standards: 2.50 ppm for residual ^1^H and 39.50 ppm for ^13^C in DMSO–*d*_6_; 7.26 ppm for residual ^1^H and 77.16 ppm for ^13^C in CDCl_3_. Multiplicities are abbreviated as follows: s, singlet; d, doublet; t, triplet; q, quartet; m, multiplet; br, broad. Coupling constants, *J*, are reported in Hertz (Hz). Melting points were determined in open capillary tubes with an Electrothermal IA 9300 (Electrothermal Engineering Ltd, Rochford, UK) series digital melting point apparatus. High-resolution mass spectra (HRMS) were measured with Bruker Maxis–qTOF (Bruker Daltonics GmbH & Co. KG, Bremen, Germany) (ESI, negative mode).

### 3.2. Synthesis and Characterization of 1,2,4-Oxadiazin-5(6H)-Ones

*General procedure for the synthesis of 1,2,4-oxadiazin-5(6H)-ones* (**3–5**). To a solution of amidoxime **1** (2 mmol) in DMSO (3 mL), *t*-BuONa (384 mg, 4 mmol) was rapidly added at room temperature. The reaction mixture was stirred at room temperature for 10 min, and ester **2** (2.4 mmol) was added. The reaction mixture was stirred at room temperature for another 18 h and was then diluted with HCl (10% solution in water) (30 mL). The resulting precipitate was collected by filtration, washed with cold water (5 mL), and air-dried at 50 °C.
*3-(4-Methylphenyl)-4H-1,2,4-oxadiazin-5(6H)-one* (**3a**). White powder; 50% yield; mp 140–141 °C. ^1^H NMR (400 MHz, CDCl_3_) *δ* 9.05 (s, 1H), 7.66 (d, *J* = 8.2 Hz, 2H), 7.31 (d, *J* = 8.0 Hz, 2H), 4.47 (s, 2H), 2.43 (s, 3H).^13^C NMR (101 MHz, CDCl_3_) *δ* 166.6, 151.0, 142.3, 129.8, 126.0, 125.7, 66.9, 21.5. HRMS (ESI), *m*/*z*: [M–H]^−^ calcd for C_10_H_9_N_2_O_2_^−^ 189.0669; found 189.0667.
*3-(4-Bromophenyl)-4H-1,2,4-oxadiazin-5(6H)-one* (**3b**). Pale beige powder; 59% yield; mp 180–181 °C. ^1^H NMR (400 MHz, DMSO-*d_6_*) *δ* 11.43 (s, 1H), 7.70 (s, 4H), 4.38 (s, 2H). ^13^C NMR (101 MHz, DMSO-*d_6_*) *δ* 166.4, 151.7, 132.4, 129.4, 129.0, 125.4, 67.2. HRMS (ESI), *m*/*z*: [M–H]^−^ calcd for C_9_H_6_BrN_2_O_2_^−^ 252.9618; found 252.9620.
*3-(4-Nitrophenyl)-4H-1,2,4-oxadiazin-5(6H)-one* (**3c**). Pale yellow powder; 52% yield; mp 248–250 °C. ^1^H NMR (400 MHz, DMSO-*d_6_*) *δ* 11.64 (s, 1H), 8.36–8.30 (m, 2H), 8.06–8.00 (m, 2H), 4.44 (s, 2H). ^13^C NMR (101 MHz, DMSO-*d_6_*) *δ* 166.0, 150.9, 149.4, 135.5, 128.7, 124.2, 67.0. HRMS (ESI), *m*/*z*: [M–H]^−^ calcd for C_9_H_6_N_3_O_4_^−^ 220.0364; found 220.0364.
*3-(5-Methylthiophen-2-yl)-4H-1,2,4-oxadiazin-5(6H)-one* (**3d**). Pale beige powder; 77% yield; mp 216–218 °C. ^1^H NMR (400 MHz, CDCl_3_) δ 9.30 (s, 1H), 7.35 (d, *J* = 3.7 Hz, 1H), 6.80 (d, *J* = 3.5 Hz, 1H), 4.47 (s, 2H), 2.54 (s, 3H). ^13^C NMR (101 MHz, DMSO-*d*_6_) *δ* 166.4, 148.4, 144.6, 129.7, 128.9, 126.8, 67.5, 15.7. HRMS (ESI), *m*/*z*: [M–H]^−^ calcd for C_8_H_8_N_2_O_2_S^−^ 195.0234; found 195.0235.
*3-(4-Methoxyphenyl)-4H-1,2,4-oxadiazin-5(6H)-one* (**3e**). White powder; 59% yield; mp 188–190 °C. ^1^H NMR (400 MHz, CDCl_3_) *δ* 8.29 (s, 1H), 7.68 (d, *J* = 8.8 Hz, 2H), 7.01 (d, *J* = 8.9 Hz, 2H), 4.47 (s, 2H), 3.89 (s, 3H). ^13^C NMR (101 MHz, DMSO-*d*_6_) *δ* 166.5, 162.0, 151.8, 128.7, 121.6, 114.5, 67.0, 55.8. HRMS (ESI), *m*/*z*: [M–H]^−^ calcd for C_10_H_9_N_2_O_3_^−^ 205.0619; found 205.0622.
*3-(4-(4-Methoxyphenoxy)phenyl)-4H-1,2,4-oxadiazin-5(6H)-one* (**3f**). White powder; 36% yield; mp 127–129 °C. ^1^H NMR (400 MHz, DMSO-*d_6_*) *δ* 11.33 (s, 1H), 7.74 (d, *J* = 8.8 Hz, 2H), 7.10–6.94 (m, 6H), 4.35 (s, 2H), 3.78 (s, 3H). ^13^C NMR (101 MHz, DMSO-*d_6_*) *δ* 166.4, 161.1, 156.7, 151.7, 148.8, 129.1, 123.4, 121.9, 117.1, 115.7, 67.0, 55.9. HRMS (ESI), *m*/*z*: [M–H]^−^ calcd for C_16_H_13_N_2_O_4_^−^ 297.0881; found 297.0880.
*6-Methyl-3-(4-methylphenyl)-4H-1,2,4-oxadiazin-5(6H)-one* (**4a**). White powder; 58% yield; mp 123–125 °C. ^1^H NMR (400 MHz, DMSO-*d_6_*) *δ* 11.25 (s, 1H), 7.64 (d, *J* = 7.8 Hz, 2H), 7.28 (d, *J* = 7.8 Hz, 2H), 4.32 (q, *J* = 6.6 Hz, 1H), 2.35 (s, 3H), 1.36 (d, *J* = 6.7 Hz, 3H). ^13^C NMR (101 MHz, DMSO-*d_6_*) *δ* 168.4, 152.0, 141.5, 129.6, 127.0, 126.7, 72.1, 21.4, 13.6. HRMS (ESI), *m*/*z*: [M–H]^−^ calcd for C_11_H_11_N_2_O_2_^−^ 203.0826; found 203.0826.
*3-(4-Bromophenyl)-6-methyl-4H-1,2,4-oxadiazin-5(6H)-one* (**4b**). White powder; 31% yield; mp 192–194 °C. ^1^H NMR (400 MHz, DMSO-*d_6_*) *δ* 11.37 (s, 1H), 7.70 (s, 4H), 4.35 (q, *J* = 6.7 Hz, 1H), 1.37 (d, *J* = 6.7 Hz, 3H). ^13^C NMR (101 MHz, DMSO-*d_6_*) *δ* 168.4, 151.6, 132.4, 129.4, 129.0, 125.4, 72.4, 13.8. HRMS (ESI), *m*/*z*: [M–H]^−^ calcd for C_10_H_8_BrN_2_O_2_^−^ 266.9775; found 266.9779.
*6-Methyl-3-(4-nitrophenyl)-4H-1,2,4-oxadiazin-5(6H)-one* (**4c**). White powder; 31% yield; mp 247–249 °C. ^1^H NMR (400 MHz, DMSO-*d_6_*) *δ* 11.59 (s, 1H), 8.32 (d, *J* = 8.5 Hz, 2H), 8.02 (d, *J* = 8.5 Hz, 2H), 4.41 (q, *J* = 6.7 Hz, 1H), 1.39 (d, *J* = 6.7 Hz, 3H). ^13^C NMR (101 MHz, DMSO-*d_6_*) *δ* 168.1, 151.1, 149.6, 135.7, 128.8, 124.4, 72.5, 13.8. HRMS (ESI), *m*/*z*: [M–H]^−^ calcd for C_11_H_11_N_2_O_3_^−^ 219.0775; found 219.0775.
*6-Methyl-3-(5-methylthiophen-2-yl)-4H-1,2,4-oxadiazin-5(6H)-one* (**4d**). Pale beige powder; 76% yield; mp 167–169 °C. ^1^H NMR (400 MHz, DMSO-*d_6_*) *δ* 11.41 (s, 1H), 7.56 (d, *J* = 3.7 Hz, 1H), 6.85 (d, *J* = 2.4 Hz, 1H), 4.34 (q, *J* = 6.7 Hz, 1H), 2.45 (s, 3H), 1.35 (d, *J* = 6.7 Hz, 3H). ^13^C NMR (101 MHz, DMSO-*d_6_*) *δ* 168.3, 148.4, 144.5, 129.6, 129.0, 126.7, 72.7, 15.7, 13.8. HRMS (ESI), *m*/*z*: [M–H]^−^ calcd for C_9_H_9_N_2_O_2_S^−^ 209.0390; found 209.0391.
*3-(4-Methoxyphenyl)-6-methyl-4H-1,2,4-oxadiazin-5(6H)-one* (**4e**). White powder; 47% yield; mp 134–136 °C. ^1^H NMR (400 MHz, DMSO-*d*_6_) δ 11.22 (s, 1H), 7.70 (d, *J* = 8.4 Hz, 2H), 7.02 (d, *J* = 8.4 Hz, 2H), 4.31–4.29 (m, 1H), 3.80 (s, 3H), 1.36 (d, *J* = 6.7 Hz, 3H). ^13^C NMR (101 MHz, DMSO-*d*_6_) δ 168.7, 162.2, 152.0, 128.9, 121.9, 114.7, 72.3, 56.1, 13.8. HRMS (ESI), *m*/*z*: [M–H]^−^ calcd for C_11_H_11_N_2_O_3_^−^ 219.0775; found 219.0775.
*6-Ethyl-3-(4-methylphenyl)-4H-1,2,4-oxadiazin-5(6H)-one* (**5a**). White powder; 55% yield; mp 112–114 °C. ^1^H NMR (400 MHz, DMSO-*d_6_*) *δ* 11.25 (s, 1H), 7.63 (d, *J* = 7.8 Hz, 2H), 7.28 (d, *J* = 7.9 Hz, 2H), 4.17 (dd, *J* = 7.7, 4.6 Hz, 1H), 2,35 (s, 3H), 1.91–1.82 (m, 1H), 1.76–1.65 (m, 1H), 1.00 (t, *J* = 7.4 Hz, 3H). ^13^C NMR (101 MHz, DMSO-*d_6_*) *δ* 168.1, 152.0, 141.7, 129.9, 127.2, 126.9, 76.7, 21.6, 21.5, 10.1. HRMS (ESI), *m*/*z*: [M–H]^−^ calcd for C_12_H_13_N_2_O_2_^−^ 217.0983; found 217.0983.
*3-(4-Bromophenyl)-6-ethyl-4H-1,2,4-oxadiazin-5(6H)-one* (**5b**). White powder; 45% yield; mp 176–178 °C. ^1^H NMR (400 MHz, DMSO-*d_6_*) *δ* 11.37 (s, 1H), 7.69 (s, 4H), 4.20 (dd, *J* = 7.7, 4.7 Hz, 1H), 1.93–1.83 (m, 1H), 1.77–1.66 (m, 1H), 1.00 (t, *J* = 7.4 Hz, 3H). ^13^C NMR (101 MHz, DMSO-*d_6_*) *δ* 167.6, 151.2, 132.1, 129.1, 128.8, 125.1, 76.6, 21.2, 9.9. HRMS (ESI), *m*/*z*: [M–H]^−^ calcd for C_11_H_10_BrN_2_O_2_^−^ 280.9931; found 280.9931.
*6-Ethyl-3-(4-nitrophenyl)-4H-1,2,4-oxadiazin-5(6H)-one* (**5c**). White powder; 49% yield; mp 177–179 °C. ^1^H NMR (400 MHz, DMSO-*d_6_*) *δ* 11.58 (s, 1H), 8.32 (d, *J* = 8.6 Hz, 2H), 8.02 (d, *J* = 8.6 Hz, 2H), 4.27 (dd, *J* = 7.7, 4.7 Hz, 1H), 1.96–1.85 (m, 1H), 1.80–1.69 (m, 1H), 1.02 (t, *J* = 7.4 Hz, 3H). ^13^C NMR (101 MHz, DMSO-*d_6_*) *δ* 167.4, 150.6, 149.3, 135.5, 128.6, 124.2, 76.7, 21.3, 9.8. HRMS (ESI), *m*/*z*: [M–H]^−^ calcd for C_11_H_10_N_3_O_4_^−^ 248.0677; found 248.0678.
*6-Ethyl-3-(5-methylthiophen-2-yl)-4H-1,2,4-oxadiazin-5(6H)-one* (**5d**). Pale beige powder; 61% yield; mp 151–153 °C. ^1^H NMR (400 MHz, DMSO-*d_6_*) *δ* 11.40 (s, 1H), 7.55 (d, *J* = 3.7 Hz, 1H), 6.85 (d, *J* = 3.7 Hz, 1H), 4.19 (dd, *J* = 7.8, 4.6 Hz, 1H), 2.45 (s, 3H), 1.90–1.81 (m, 1H), 1.73–1.65 (m, 1H), 0.99 (t, *J* = 7.4 Hz, 3H). ^13^C NMR (101 MHz, DMSO-*d_6_*) *δ* 167.8, 148.2, 144.5, 129.6, 129.0, 126.7, 77.2, 21.5, 15.7, 10.0. HRMS (ESI), *m*/*z*: [M–H]^−^ calcd for C_10_H_11_N_2_O_2_S^−^ 223.0547; found 223.0554.
*6-Ethyl-3-(4-methoxyphenyl)-4H-1,2,4-oxadiazin-5(6H)-one* (**5e**). Pale beige powder; 67% yield; mp 139–141 °C. ^1^H NMR (400 MHz, DMSO-*d_6_*) *δ* 11.21 (s, 1H), 7.69 (d, *J* = 8.5 Hz, 2H), 7.02 (d, *J* = 8.5 Hz, 2H), 4.15 (dd, *J* = 7.8, 4.6 Hz, 1H), 3,8 (s, 3H), 1.92–1.82 (m, 1H), 1.75–1.64 (m, 1H), 1.00 (t, *J* = 7.4 Hz, 3H). ^13^C NMR (101 MHz, DMSO-*d_6_*) *δ* 168.2, 162.1, 151.8, 128.9, 121.8, 114.7, 76.7, 56.1, 21.5, 10.1. HRMS (ESI), *m*/*z*: [M–H]^−^ calcd for C_12_H_13_N_2_O_3_^−^ 233.0932; found 233.0930.
*6-Ethyl-3-(4-(4-methoxyphenoxy)phenyl)-4H-1,2,4-oxadiazin-5(6H)-one* (**5f**). Pale beige powder; 38% yield; mp 117–119 °C. ^1^H NMR (400 MHz, DMSO-*d_6_*) *δ* 11.26 (s, 1H), 7.72 (d, *J* = 8.4 Hz, 2H), 7.09–6.92 (m, 6H), 4.16 (dd, *J* = 7.8, 4.6 Hz, 1H), 3.76 (s, 3H), 1.90–1.83 (m, 1H), 1.75–1.66 (m, 1H), 1.00 (t, *J* = 7.4 Hz, 3H). ^13^C NMR (101 MHz, DMSO-*d_6_*) *δ* 168.0, 161.2, 156.9, 151.6, 149.0, 129.2, 123.7, 122.1, 117.4, 116.0, 76.8, 56.1, 21.5, 10.1. HRMS (ESI), *m*/*z*: [M–H]^−^ calcd for C_18_H_17_N_2_O_4_^−^ 325.1194; found 325.1219.
*6-Ethyl-3-phenyl-4H-1,2,4-oxadiazin-5(6H)-one* (**5g**). White powder; 45% yield; mp 133–135 °C. ^1^H NMR (400 MHz, DMSO-*d_6_*) *δ* 11.31 (s, 1H), 7.74 (d, *J* = 7.5 Hz, 2H), 7.56–7.46 (m, 3H), 4.20 (dd, *J* = 7.8, 4.6 Hz, 1H), 1.92–1.83 (m, 1H), 1.77–1.66 (m, 1H), 1.01 (t, *J* = 7.4 Hz, 3H). ^13^C NMR (101 MHz, DMSO-*d_6_*) *δ* 168.0, 152.1, 131.8, 129.8, 129.3, 127.3, 76.8, 21.5, 10.1. HRMS (ESI), *m*/*z*: [M–H]^−^ calcd for C_11_H_11_N_2_O_2_^−^ 203.0826; found 203.0826.
*6-Ethyl-3-(4-fluorophenyl)-4H-1,2,4-oxadiazin-5(6H)-one* (**5h**). White powder; 36% yield; mp 153–155 °C. ^1^H NMR (400 MHz, DMSO-*d_6_*) *δ* 11.33 (s, 1H), 7.89–7.73 (m, 2H), 7.35–7.31 (m, 2H), 4.19 (dd, *J* = 7.7, 4.6 Hz, 1H), 1.92–1.88 (m, 1H), 1.77–1.66 (m, 1H), 1.01 (t, *J* = 7.4 Hz, 3H). ^13^C NMR (101 MHz, DMSO-*d_6_*) *δ* 167.9, 165.6, 163.2, 151.4, 129.9 (d, *J* = 8.8 Hz, 1C), 126.3, 116.4 (d, *J* = 22.0 Hz, 1C), 76.8, 21.4, 10.1. ^19^F NMR (376 MHz, CDCl_3_) *δ* –107.49. HRMS (ESI), *m*/*z*: [M–H]^−^ calcd for C_11_H_10_FN_2_O_2_^−^ 221.0732; found 221.0732.
*3-Benzyl-6-ethyl-4H-1,2,4-oxadiazin-5(6H)-one* (**5i**). White powder; 33% yield; mp 97–99 °C. ^1^H NMR (400 MHz, DMSO-*d_6_*) *δ* 10.91 (s, 1H), 7.35–7. 26 (m, 5H), 4.02 (dd, *J* = 7.8, 4.7 Hz, 1H), 3.54 (s, 2H), 1.79–1.69 (m, 1H), 1.63–1.52 (m, 1H), 0.94 (t, *J* = 7.4 Hz, 3H). ^13^C NMR (101 MHz, DMSO-*d_6_*) *δ* 168.0, 152.9, 136.1, 129.4, 129.2, 127.7, 76.4, 36.5, 21.5, 10.1. HRMS (ESI), *m*/*z*: [M–H]^−^ calcd for C_12_H_13_N_2_O_2_^−^ 217.0983; found 217.0983.

General procedure for preparation acids **8f** and **8g**. To a solution of amidoxime **1** (2 mmol) and dimethylmaleate (4 mmol) in DMSO (3 mL), powdered NaOH (240 mg, 4 mmol) was rapidly added. The reaction mixture was stirred at room temperature for 18 h, diluted with cold brine (30 mL) and washed twice with toluene (5 mL). The aqueous solution was acidified with hydrochloric acid to a pH of approximately 1 and cooled to 5 °C. The resulting precipitate was collected by filtration, washed with cold water (5 mL), and air-dried at 50 °C.

*2-(3-(3-Chlorophenyl)-5-oxo-5,6-dihydro-4H-1,2,4-oxadiazin-6-yl)acetic acid* (**8f**). Beige powder; 71% yield (380 mg); mp 211–212 °C. ^1^H NMR (400 MHz, DMSO-*d*_6_) δ 12.55 (s, 1H), 11.45 (s, 1H), 7.80 (t, *J* = 1.9 Hz, 1H), 7.72 (dt, *J* = 7.9, 1.3 Hz, 1H), 7.62 (ddd, *J* = 8.1, 2.2, 1.1 Hz, 1H), 7.52 (t, *J* = 7.9 Hz, 1H), 4.59 (dd, *J* = 7.7, 4.5 Hz, 1H), 2.93 (dd, *J* = 16.8, 4.5 Hz, 1H), 2.68 (dd, *J* = 16.8, 7.7 Hz, 1H). ^13^C NMR (101 MHz, DMSO-*d_6_*) δ 170.8, 166.4, 150.8, 133.4, 131.1, 131.0, 130.6, 126.7, 125.5, 72.5, 33.0. HRMS (ESI), *m*/*z*: [M+H]^+^ calcd. for C_11_H_10_ClN_2_O_4_^+^ 269.0324; found 269.0321.
*2-(5-Oxo-3-(4-(trifluoromethoxy)phenyl)-5,6-dihydro-4H-1,2,4-oxadiazin-6-yl)acetic acid* (**8g**). White powder; 77% yield (488 mg); mp 208–211 °C. ^1^H NMR (400 MHz, DMSO-*d*_6_) δ 11.49 (s, 1H), 7.96–7.77 (m, 2H), 7.49 (d, *J* = 8.4 Hz, 2H), 4.59 (dd, *J* = 7.7, 4.5 Hz, 1H), 2.93 (dd, *J* = 16.9, 4.4 Hz, 1H), 2.68 (dd, *J* = 16.7, 7.8 Hz, 1H). ^13^C NMR (101 MHz, DMSO-*d_6_*) δ 186.2, 171.4, 167.11, 151.6, 150.9, 129.8, 128.9, 121.9, 120.6 120.6 (q, *J* = 257.1 Hz), 73.1, 33.6. HRMS (ESI), *m*/*z*: [M+H]^+^ calcd. for C_12_H_10_F_3_N_2_O_5_^+^ 319.0536; found 319.0539.

### 3.3. X-Ray Studies

Singe crystals for X-ray diffraction were obtained by slow evaporation of solutions of 1,2,4-oxadiazin-5(6*H*)-ones in CDCl_3_ at room temperature in air. Crystals of **3a** and **5b** were analyzed at 100(2) K on a Rigaku XtaLAB Synergy-S diffractometer (HyPix-6000HE type detector) using Cu Kα (λ = 1.54184 Å) radiation. The structures were solved with the ShelXT [36] structure solution program using Intrinsic Phasing and refined with the ShelXL [37] refinement package incorporated in the OLEX2 program package [38] using Least Squares minimization. The hydrogen atoms in all structures were placed in ideally calculated positions according to neutron diffraction statistical data [39] and were refined as colliding atoms with parameters of relative isotropic displacement. Supplementary crystallographic data for this paper can be obtained free of charge from The Cambridge Crystallographic Data Centre via http://www.ccdc.cam.ac.uk (accessed on 2 September 2024). CCDC numbers 2376673 (**3a**), 2376674 (**5b**).

### 3.4. MAO Inhibition Studies

The inhibition potencies of the 1,2,4-oxadiazin-5(6*H*)-one derivatives were determined using commercially available recombinant human MAO-A and MAO-B (Merck) according to the published procedure [32]. Stock solutions of all compounds were prepared in DMSO and added to the enzyme reactions to yield a final concentration of 4% DMSO. Visual inspection of the enzyme reactions found no indication that the inhibitors precipitated from the aqueous reaction buffer at higher concentrations (e.g., 100 µM). Kynuramine was used as enzyme substrate for both MAO isoforms and was oxidized to ultimately form 4-hydroxyquinoline, which is a fluorescent metabolite [31]. The concentrations of 4-hydroxyquinoline in the reactions were measured at end-point by fluorescence spectrometry (λ_ex_ = 310 nm; λ_em_ = 400 nm). Using this approach, the rates of kynuramine oxidation by MAO were measured in the presence of different concentrations of the test inhibitors (0.003–100 µM) as well as in the absence of inhibitor. These data were used to construct sigmoidal curves of the rate versus the logarithm of inhibitor concentration (log[I]), which were subsequently fitted to the one-site competition model of the Prism 5 software package (GraphPad). IC_50_ values were estimated from these plots and were reported as the mean ± SD of triplicate determinations.

To investigate the mode of inhibition, Lineweaver–Burk plots were constructed. A set of six plots were constructed for each test inhibitor using five inhibitor concentrations that bracketed the IC_50_ value as well as one plot in the absence of inhibitor. For each line, eight different concentrations of kynuramine (15–250 µM) were used. Subsequently, the slopes of the Lineweaver–Burk plots were plotted versus inhibitor concentration, and the K_i_ values were estimated from the x-intercept (K_i_ = −x-intercept).

### 3.5. Molecular Docking Studies Studies

Molecular docking was performed with the CDOCKER module of the Discovery Studio 3.1 software according to the procedure reported in literature [32]. The X-ray crystal structure of MAO-B (PDB code: 2V5Z) bound to safinamide was selected as the protein model and was prepared for the simulations in Discovery Studio [40]. The presentations were prepared with the PyMOL molecular graphics system (version 1.7.4.0) [41].

## 4. Conclusions

In this study, the syntheses of series of 1,2,4-oxadiazin-5(6*H*)-one derivatives and their MAO inhibition properties were described. The optimized reaction conditions for the condensation of amidoximes **1** with alkyl 2-halocarboxylates **2** to yield the desired 1,2,4-oxadiazin-5(6*H*)-ones were determined with respect to base, reagent amounts, solvent and reaction time. *t*-BuONa (2 equiv.) in DMSO as solvent and a reaction time of 18 h gave the highest yield. The results of the MAO inhibition studies showed that only seven derivatives exhibited IC_50_ < 10 µM for the inhibition of MAO-B, while only four derivatives exhibited IC_50_ < 15 µM for the inhibition of MAO-A. The most potent MAO-A inhibitor was **3f** with an IC_50_ value of 3.75 µM. Two compounds were found to inhibit MAO-B with submicromolar potencies, **5f** and **7c**, with IC_50_ values of 0.900 and 0.371 µM, respectively. The 1,2,4-oxadiazin-5(6*H*)-one derivatives differed regarding substitution on the C3 and C6 positions. An analysis of the structure–activity relationships suggested that ethyl substitution on C6 was in general more optimal for MAO-B inhibition than the corresponding methyl substituted and unsubstituted compounds. In this regard, five of the most potent MAO-B inhibitors (**5a**, **5b**, **5d**, **5e** and **5f**) were substituted with an ethyl group on C6. For the C3 position, the 4-(methoxyphenoxy)phenyl substituent was highlighted as being present in two good potency MAO-B inhibitors (**3f** and **5f**) and one good potency MAO-A inhibitor (**3f**). Using Lineweaver–Burk plots, it was shown that the inhibition of MAO-A and MAO-B by **3f** and **5f**, respectively, was competitive. It may thus be concluded that the active 1,2,4-oxadiazin-5(6*H*)-one derivatives identified in this study could be used as leads for the development of new therapies for disorders that have been linked to increased MAO activity.

## Figures and Tables

**Table 1:** Optimization of the condensation of amidoxime **1a** with 2-halocarboxylic acid esters **2** ^a^.

	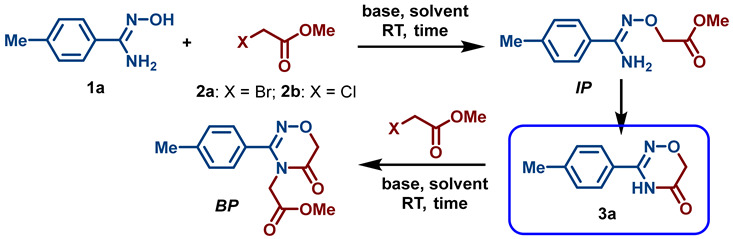					
Entry	Compound 2 (Equiv.)	X	Base (Equiv.)	Solvent	Time, h	Isolated Yield, %
1	**2a** (1.2)	Br	NaOH (1.2)	DMSO	4	– ^b^
2	**2a** (1.2)	Br	NaOH (1.5)	DMSO	4	– ^b^
3	**2a** (1.2)	Br	NaOH (2.0)	DMSO	18	29
4	**2a** (1.2)	Br	*t*-BuONa (1.2)	DMSO	4	– ^b^
5	**2a** (1.2)	Br	*t*-BuONa (1.5)	DMSO	4	– ^c^
6	**2a** (1.2)	Br	*t*-BuONa (2.0)	DMSO	18	42
7	**2b** (1.2)	Cl	NaOH (2.0)	DMSO	18	35
8	**2b** (1.2)	Cl	KOH (2.0)	DMSO	18	38
9	**2b** (1.2)	Cl	*t*-BuONa (2.0)	DMSO	18	50
10	**2b** (1.2)	Cl	*t*-BuOK (2.0)	DMSO	18	32
11	**2b** (1.2)	Cl	*t*-BuONa (2.0)	DMAA	18	37
12	**2b** (1.2)	Cl	*t*-BuONa (2.0)	DMF	18	43
13	**2b** (1.2)	Cl	*t*-BuONa (2.0)	DMSO	48	45
14	**2b** (1.5)	Cl	*t*-BuONa (2.0)	DMSO	18	13

^a^ Conditions: Compound **1a** (2 mmol), solvent (3 mL), RT. ^b^ The isolated product: methyl 2-(((amino(4-methylphenyl)methylene)amino)oxy)acetate ***IP***. ^c^ The isolated product: methyl 2-(5-oxo-3-(4-methylphenyl)-5,6-dihydro-4*H*-1,2,4-oxadiazin-4-yl)acetate ***BP***.

**Table 2:** The human MAO inhibition properties of oxadiazine derivatives **3**–**8** ^a,b^.

	IC_50_ (µM ± SD)			IC_50_ (µM ± SD)	
	MAO-A	MAO-B		MAO-A	MAO-B
**3a**	36.8 ± 1.83	37.2 ± 0.071	**6a**	NI ^c^	NI ^c^
**3b**	14.2 ± 0.940	13.6 ± 6.63	**6b**	34.2 ± 6.15	23.6 ± 1.11
**3c**	17.3 ± 0.594	24.4 ± 2.48	**6c**	NI ^c^	52.3 ± 12.7
**3d**	34.6 ± 5.47	16.6 ± 1.67	**6d**	40.7 ± 8.83	33.9 ± 1.44
**3e**	27.1 ± 3.98	37.6 ± 9.47	**6e**	NI ^c^	NI ^c^
**3f**	3.75 ± 0.212	3.98 ± 0.518	**6f**	NI ^c^	22.9 ± 5.27
**4a**	67.0 ± 0.007	24.5 ± 0.198	**7a**	54.3 ± 14.3	166 ± 49.4
**4b**	37.4 ± 4.18	27.1 ± 3.37	**7b**	NI ^c^	14.3 ± 3.27
**4c**	58.5 ± 4.17	14.6 ± 0.092	**7c**	12.9 ± 0.014	0.371 ± 0.037
**4d**	46.2 ± 9.22	22.3 ± 0.940	**7d**	NI ^c^	46.3 ± 17.8
**4e**	51.2 ± 15.07	20.0 ± 0.643	**8a**	NI ^c^	109 ± 0.566
**5a**	NI ^c^	4.28 ± 0.318	**8b**	12.7 ± 1.34	NI ^c^
**5b**	35.4 ± 1.54	5.11 ± 0.308	**8c**	NI ^c^	NI ^c^
**5c**	57.9 ± 9.84	21.0 ± 4.09	**8d**	NI ^c^	NI ^c^
**5d**	58.1 ± 5.28	6.14 ± 0.137	**8e**	NI ^c^	62.2 ± 15.7
**5e**	44.1 ± 6.05	6.22 ± 0.596	**8f**	NI ^c^	NI ^c^
**5f**	15.4 ± 1.98	0.900 ± 0.050	**8g**	NI ^c^	NI ^c^
**5g**	NI ^c^	NI ^c^			
**5h**	93.0 ± 3.23	30.7 ± 2.02			
**5i**	NI ^c^	NI ^c^			
Isatin ^d^	8.02 ± 1.69	3.64 ± 0.476			
Harmine ^d^	0.0047 ± 0.0001	NI ^c^			
Safinamide ^d^	NI ^c^	0.198 ± 0.014			

^a^ Values are given as the mean ± SD of triplicate determinations. ^b^ See Scheme 1 and Scheme 2 for structures. ^c^ No inhibition observed at the maximal tested concentration of 100 µM. ^d^ Reference inhibitors.

## Data Availability

Data are contained within the article and Supporting Materials. In addition, CIFs are openly available in https://www.ccdc.cam.ac.uk/structures/ (accessed on 2 September 2024).

## Supplementary Materials

The following supporting information can be downloaded at: https://www.mdpi.com/article/10.3390/molecules29235550/s1, and contains copies of NMR spectra for products **3**–**5** and crystallographic information for compounds **3a** and **5b** (Table S1, Figures S1 and S2).
