# Supplementary material for: Synthesis of 1,2,4-Oxadiazin-5(6H)-One Derivatives and Their Biological Investigation as Monoamine Oxidase Inhibitors
Source: Molecules. 2024 Nov 25;29(23):5550. doi: 10.3390/molecules29235550 (PMC11643077; doi:10.3390/molecules29235550)
Supplement: Supplementary file 1 [file molecules-29-05550-s001.zip › molecules-3225890-supplementary.pdf]

Supporting Information

Synthesis of 1,2,4-oxadiazin-5(6*H*)-one derivatives and their biological investigation as monoamine oxidase inhibitors

Sofia I. Presnukhina[1], Valentina D. Kotlyarova[2], Anton A. Shetnev[3,6], Sergey V. Baykov[1,3], Rakhymzhan Turmanov[3,4,*], Nurbol Appazov[3,5], Rakhmetulla Zhapparbergenov[3,4], Leilya Zhussupova[3], Nurila Togyzbayeva[3], Stephanus J. Cloete[7], Mikhail K. Korsakov[2,3], Vadim P. Boyarskiy[1], Anél Petzer[7], Jacobus P. Petzer[7,*]

[1] *Institute of Chemistry, Saint Petersburg State University, Universitetskaya Nab., 7/9, 199034 Saint Petersburg, Russian Federation*

[2] *Pharmaceutical Technology Transfer Centre, Yaroslavl State Pedagogical University named after K.D. Ushinsky, Respublikanskaya St., 108, 150000 Yaroslavl, Russian Federation*

[3] *Laboratory of Engineering Profile "Physical and Chemical Methods of Analysis", Korkyt Ata Kyzylorda University, 29 Aiteke bi str., Kyzylorda, Kazakhstan*

[4] *"DPS Kyzylorda" LLP, Amangeldy Imanov Str., 112A, Kyzylorda 120008, Kazakhstan*

[5] *''CNEC'' LLP, Dariger Ali Lane, 2, Kyzylorda 120001, Kazakhstan*

[6] *Moscow Center for Advanced Studies, 20, Kulakova Str., Moscow, Russia*

[7] *Pharmaceutical Chemistry and Centre of Excellence for Pharmaceutical Sciences, North-West University, Potchefstroom 2520, South Africa*

*Corresponding authors:

Rakhymzhan Turmanov, email: t.rahimjan.91@mail.ru; Tel.: +7 7242 23 10 41

Jacobus P. Petzer, email: Jacques.petzer@nwu.ac.za; Tel.: +27 18 2992206

Table of Content

S1. ^{1}H, ^{13}C, a ^{19}F NMR spectra of 1,2,4-oxadiazin-5(6*H*)-ones 3

S2. X–ray diffraction data for compounds **3a** and **5b** 26

S1. ^{1}H, ^{13}C, and ^{19}F NMR spectra of 1,2,4-oxadiazin-5(6*H*)-ones

^{1}H and ^{13}C spectra of 3-(4-methylphenyl)-4*H*-1,2,4-oxadiazin-5(6*H*)-one (**3a**)

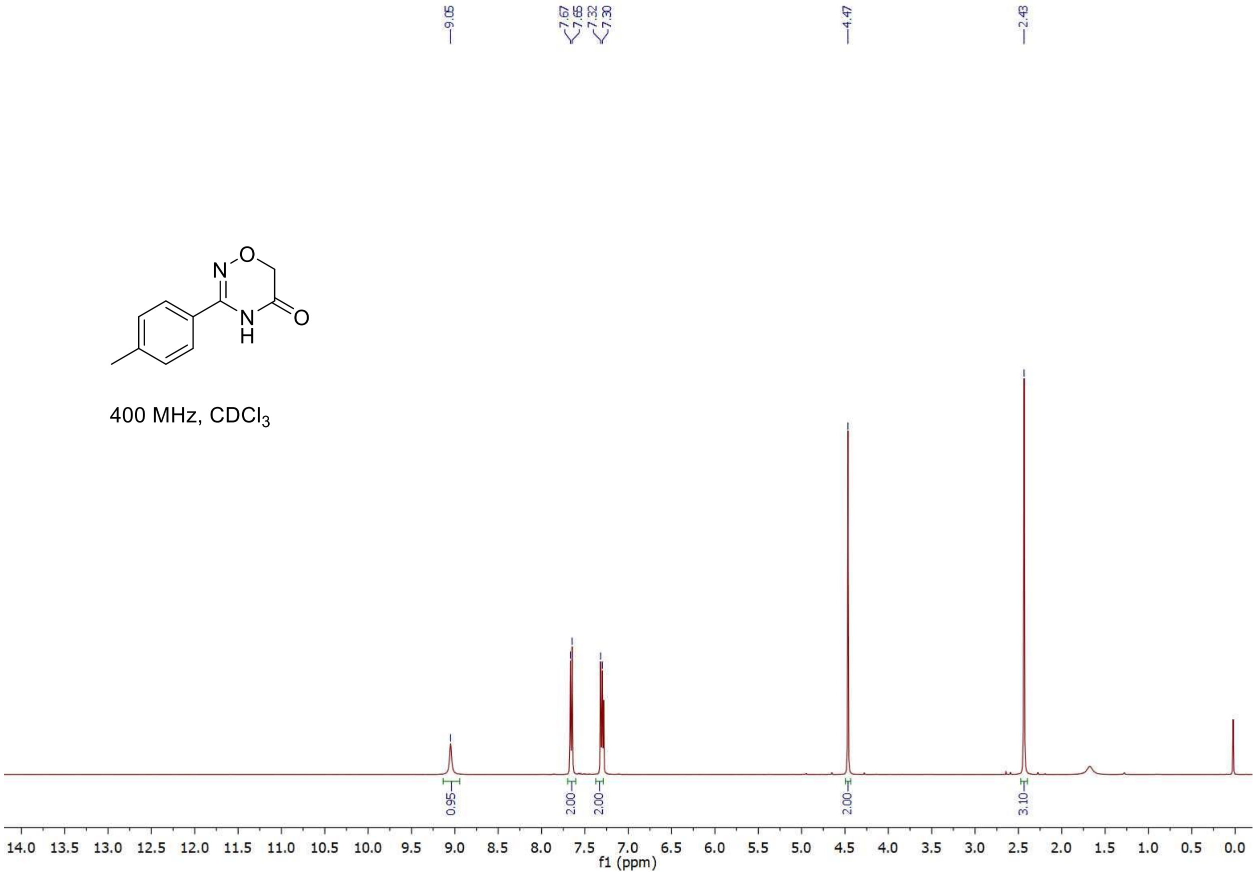

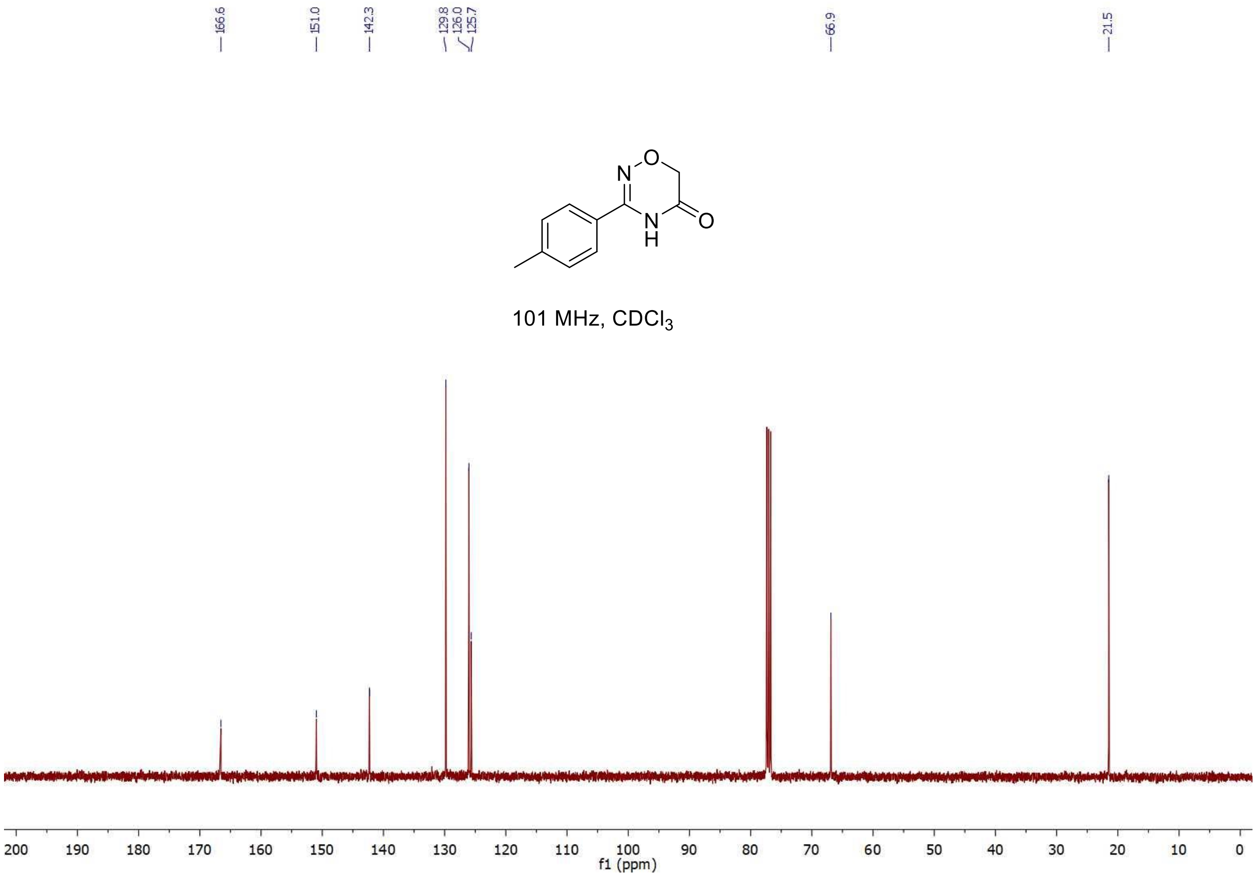

^{1}H and ^{13}C spectra of 3-(4-bromophenyl)-4*H*-1,2,4-oxadiazin-5(6*H*)-one (**3b**)

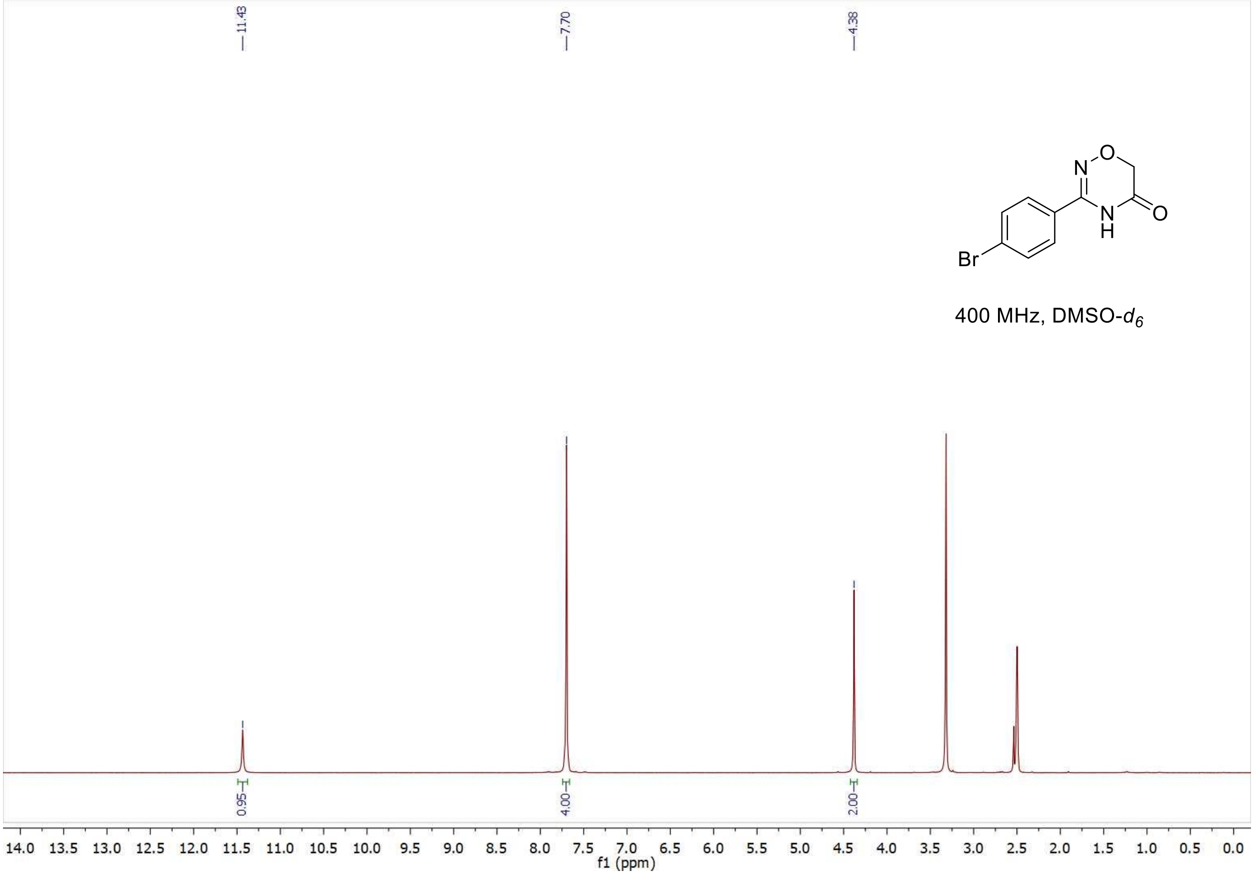

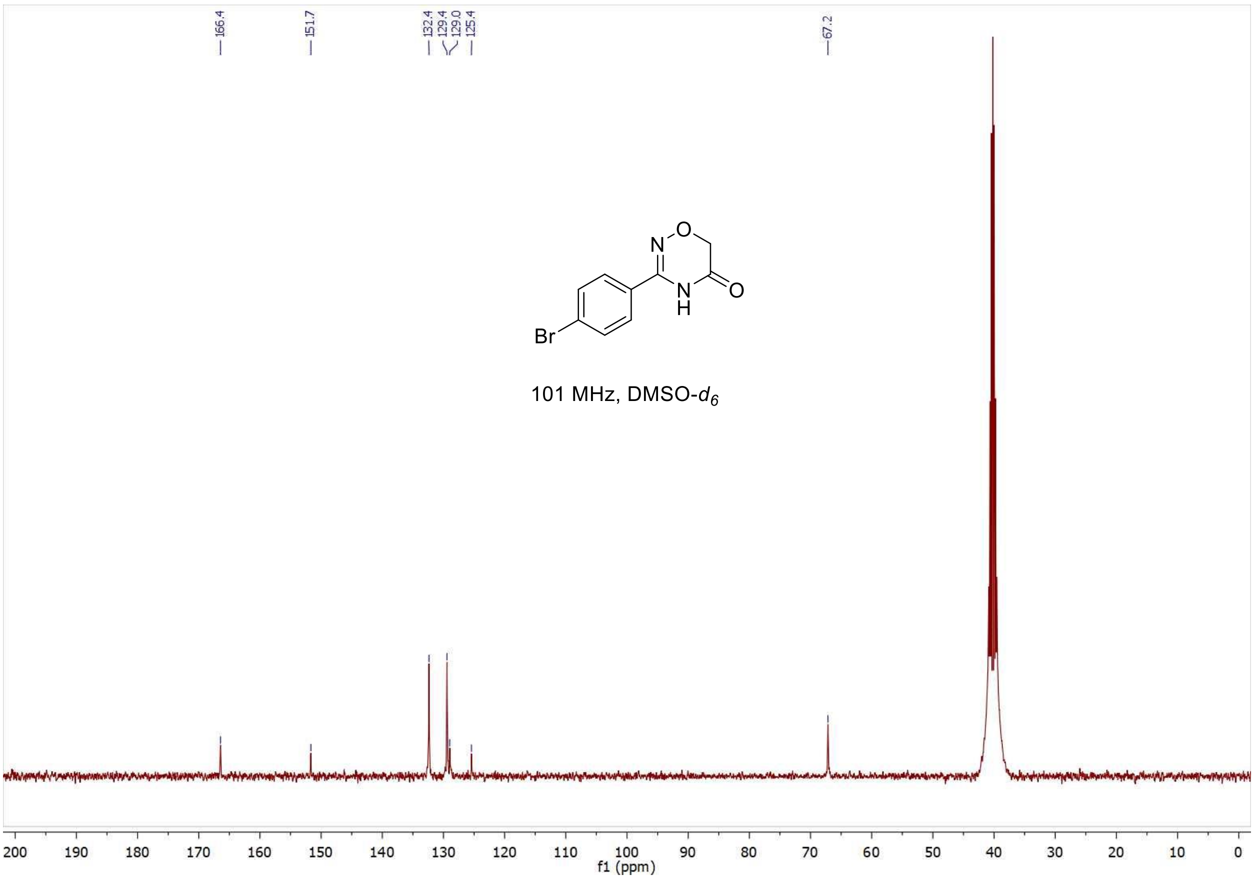

^{1}H and ^{13}C spectra of 3-(4-nitrophenyl)-4*H*-1,2,4-oxadiazin-5(6*H*)-one (**3c**)

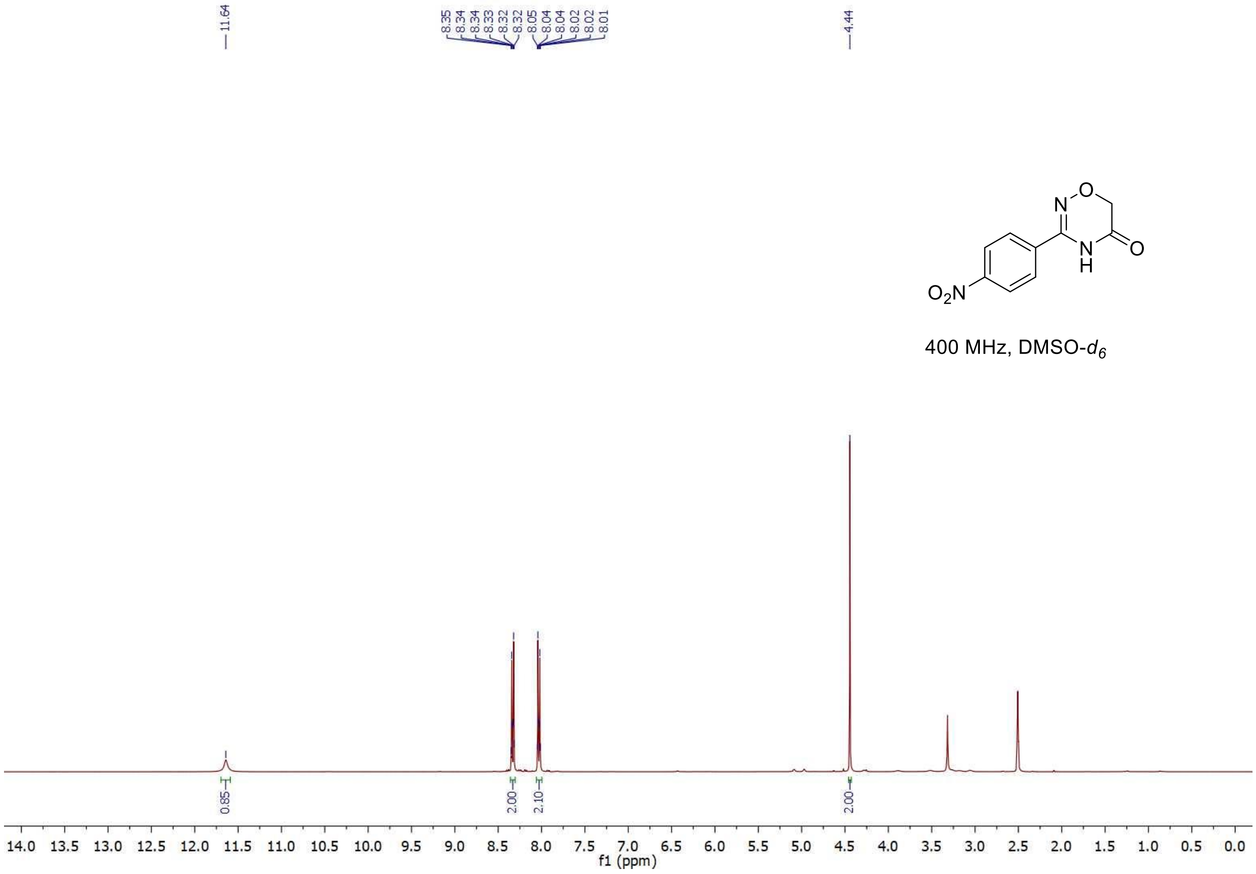

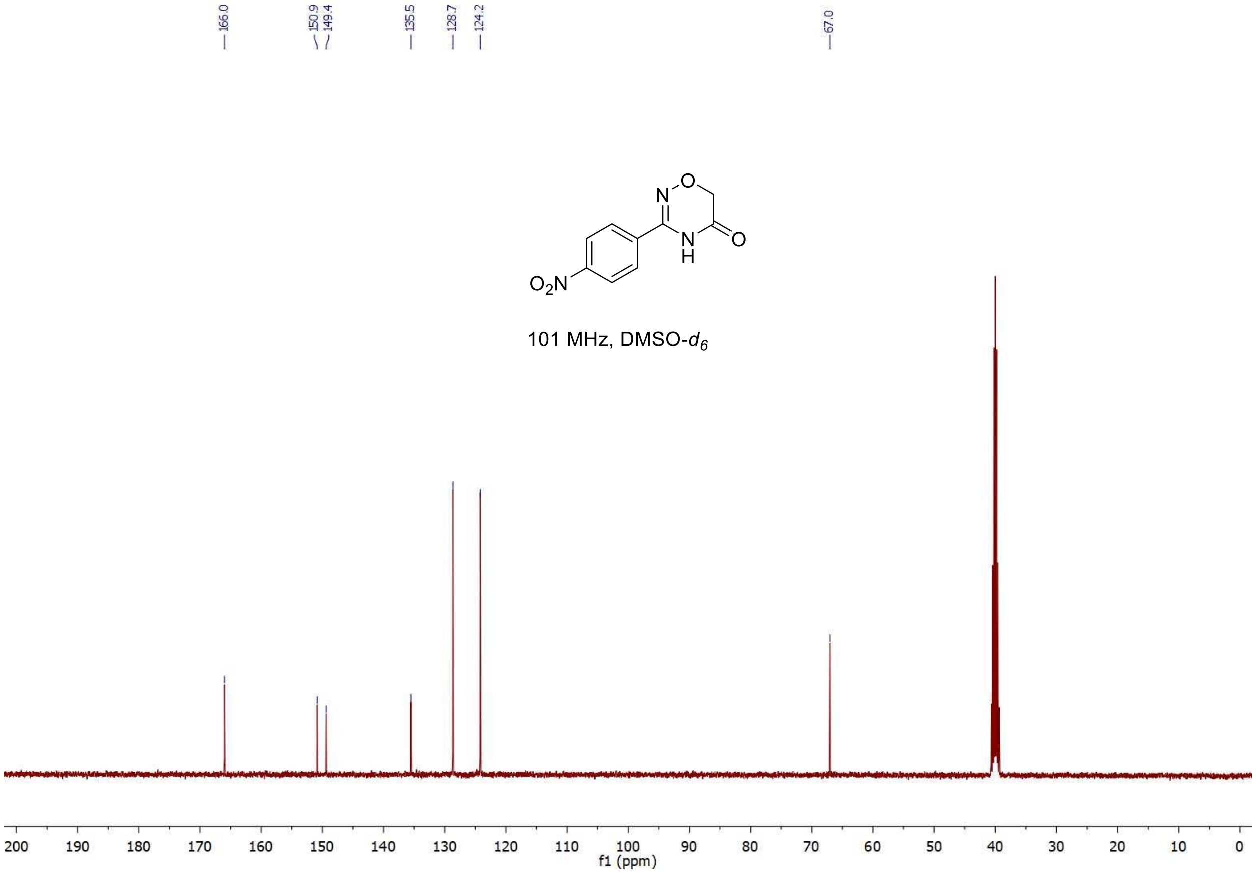

^{1}H and ^{13}C spectra of 3-(5-methylthiophen-2-yl)-4*H*-1,2,4-oxadiazin-5(6*H*)-one (**3d**)

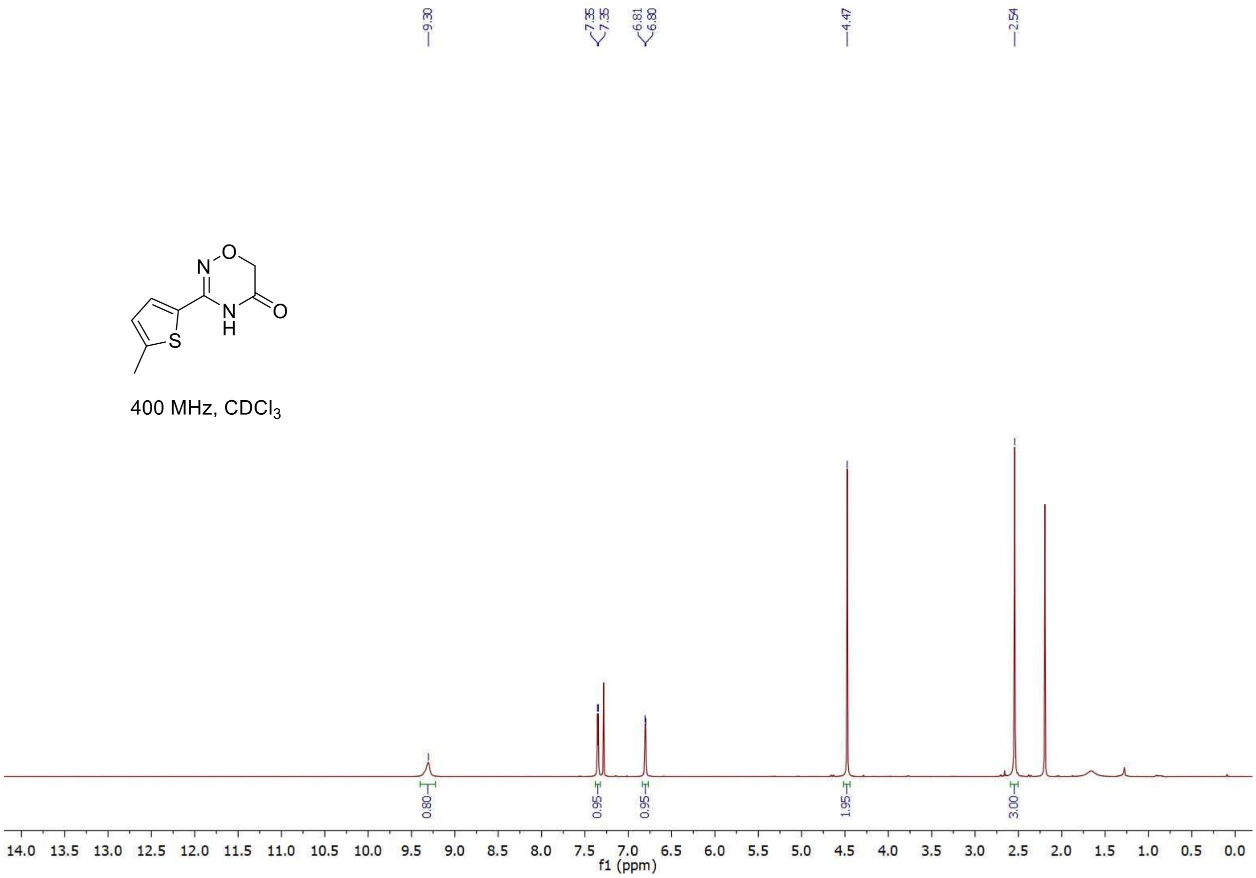

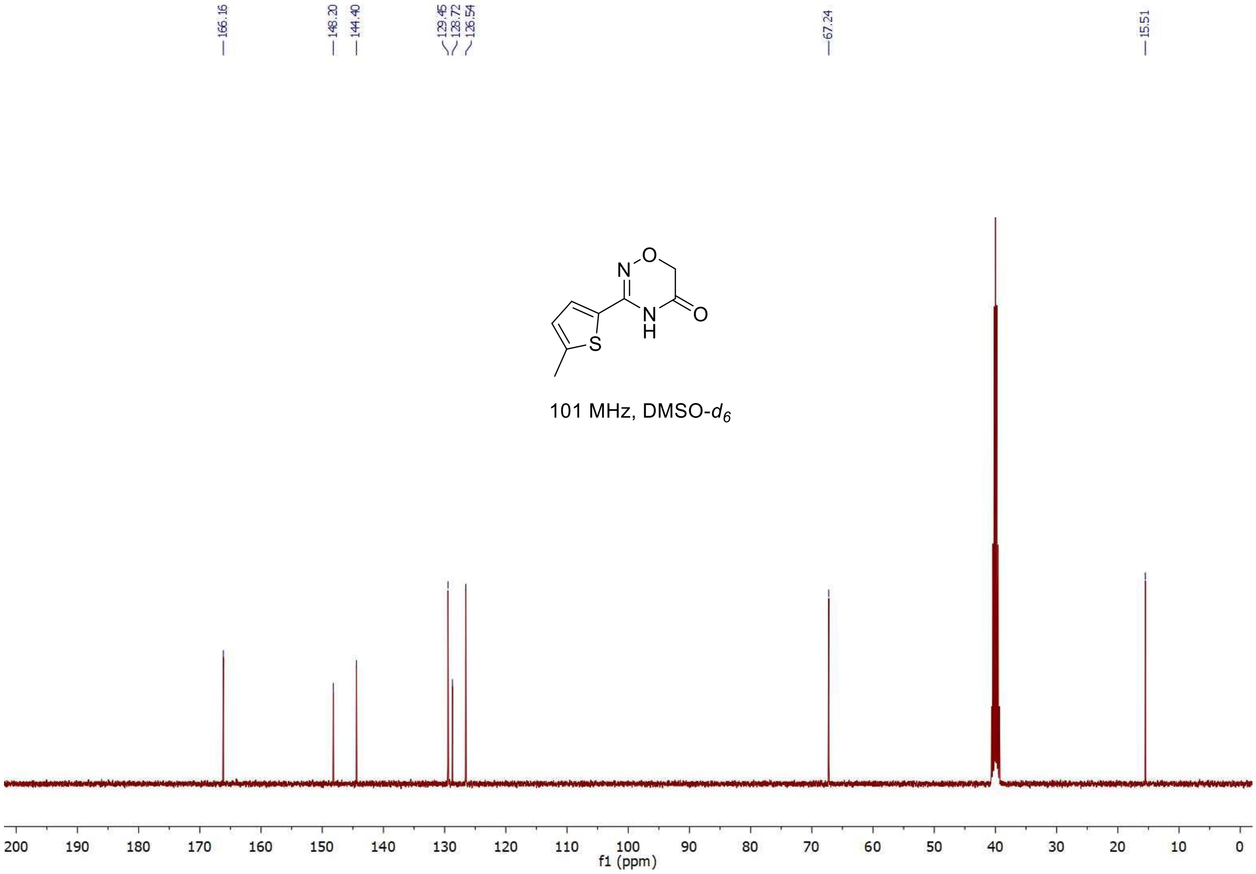

^{1}H and ^{13}C spectra of 3-(4-methoxyphenyl)-4*H*-1,2,4-oxadiazin-5(6*H*)-one (**3e**)

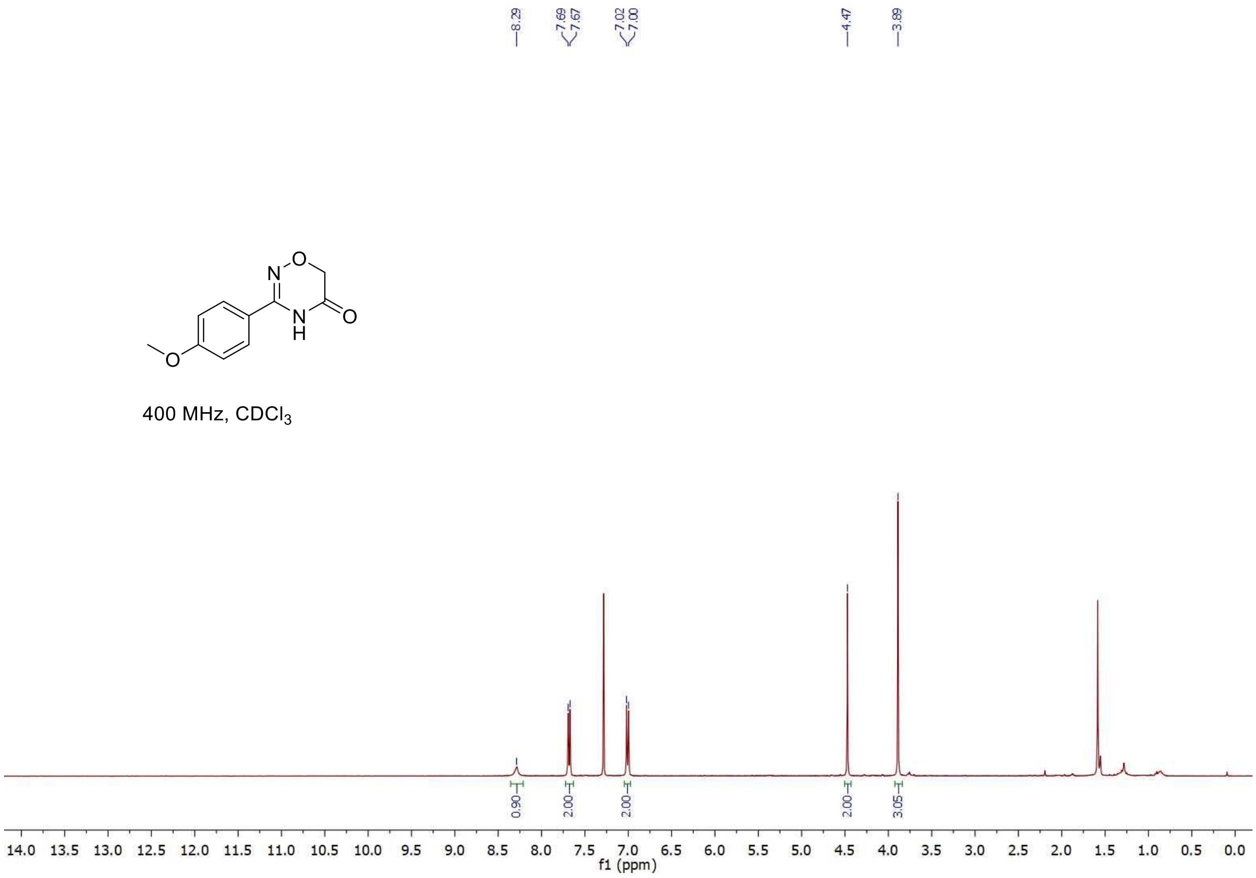

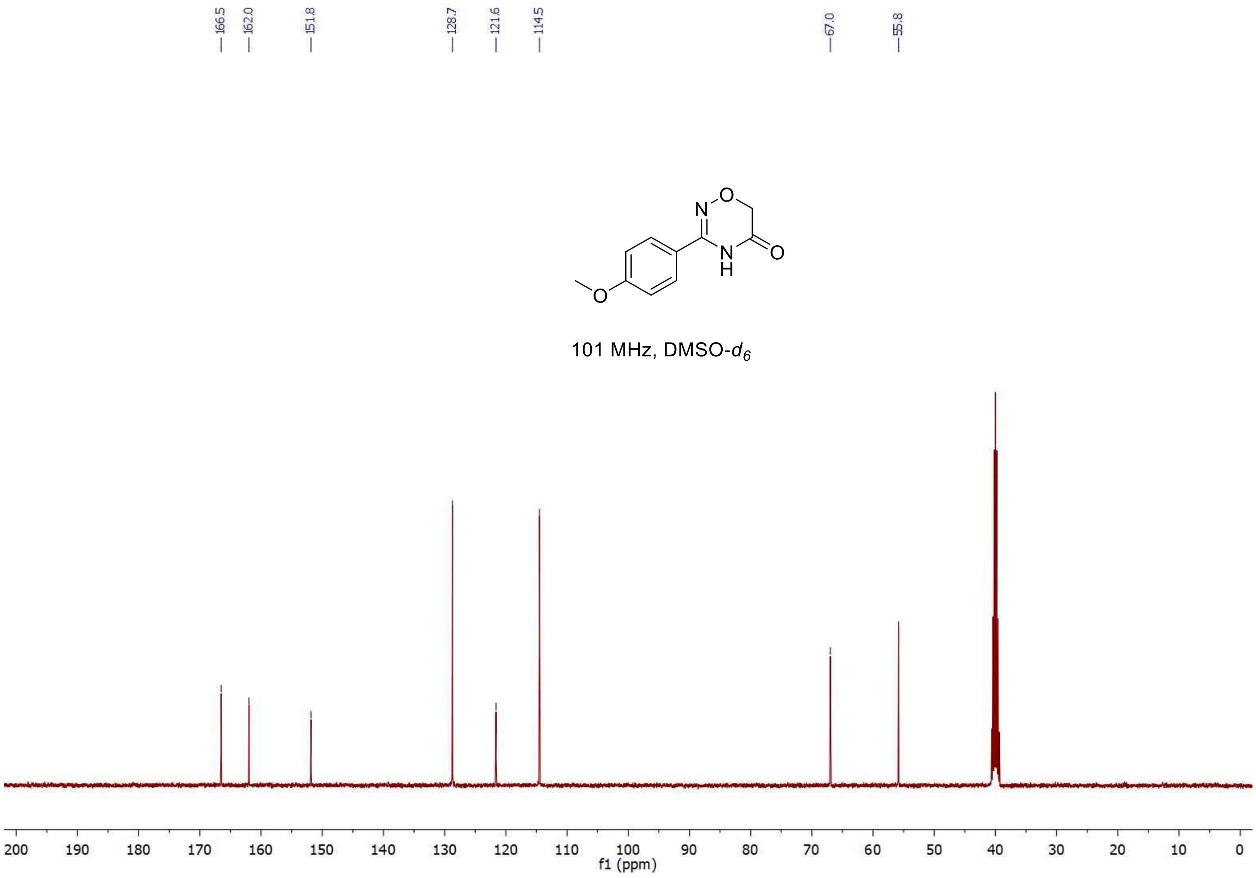

^{1}H and ^{13}C spectra 3-(4-(4-methoxyphenoxy)phenyl)-4*H*-1,2,4-oxadiazin-5(6*H*)-one (**3f**)

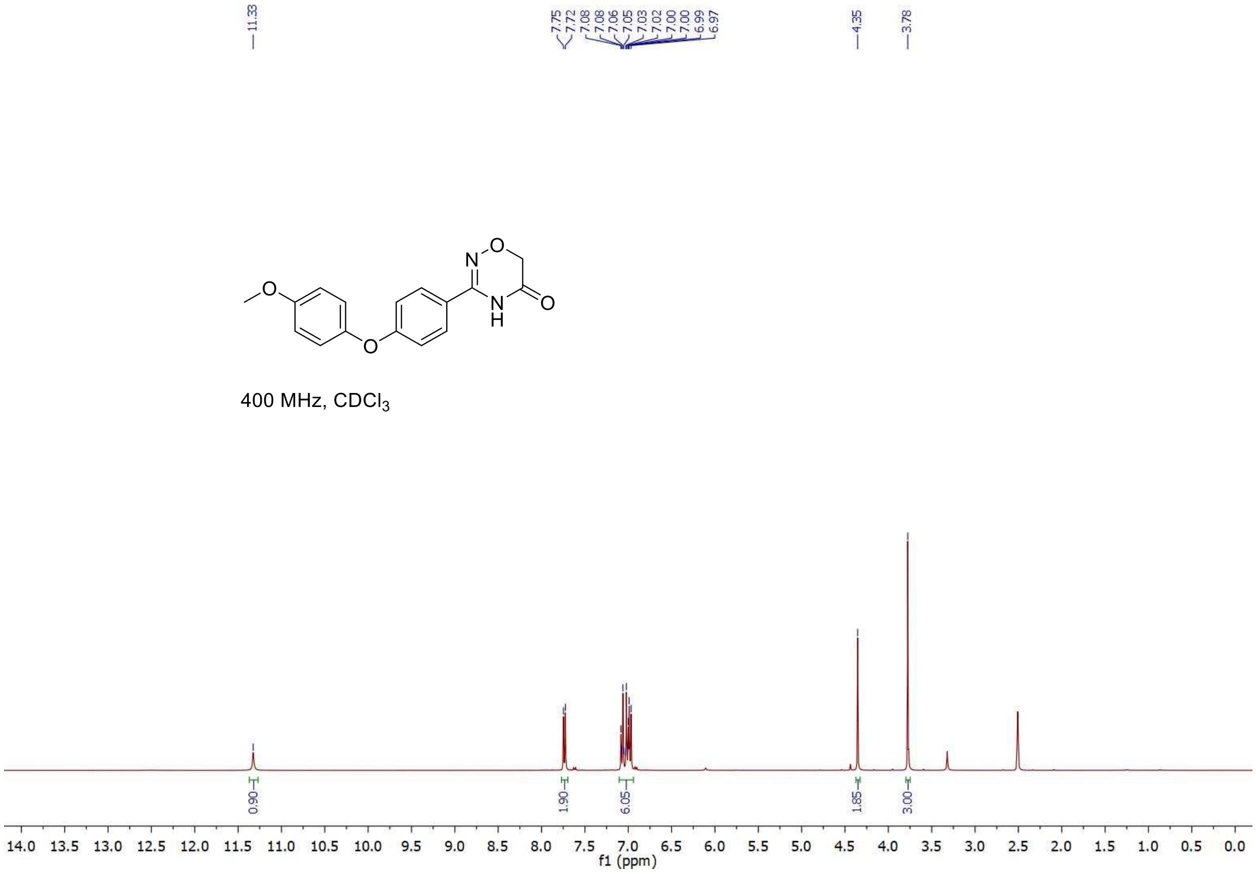

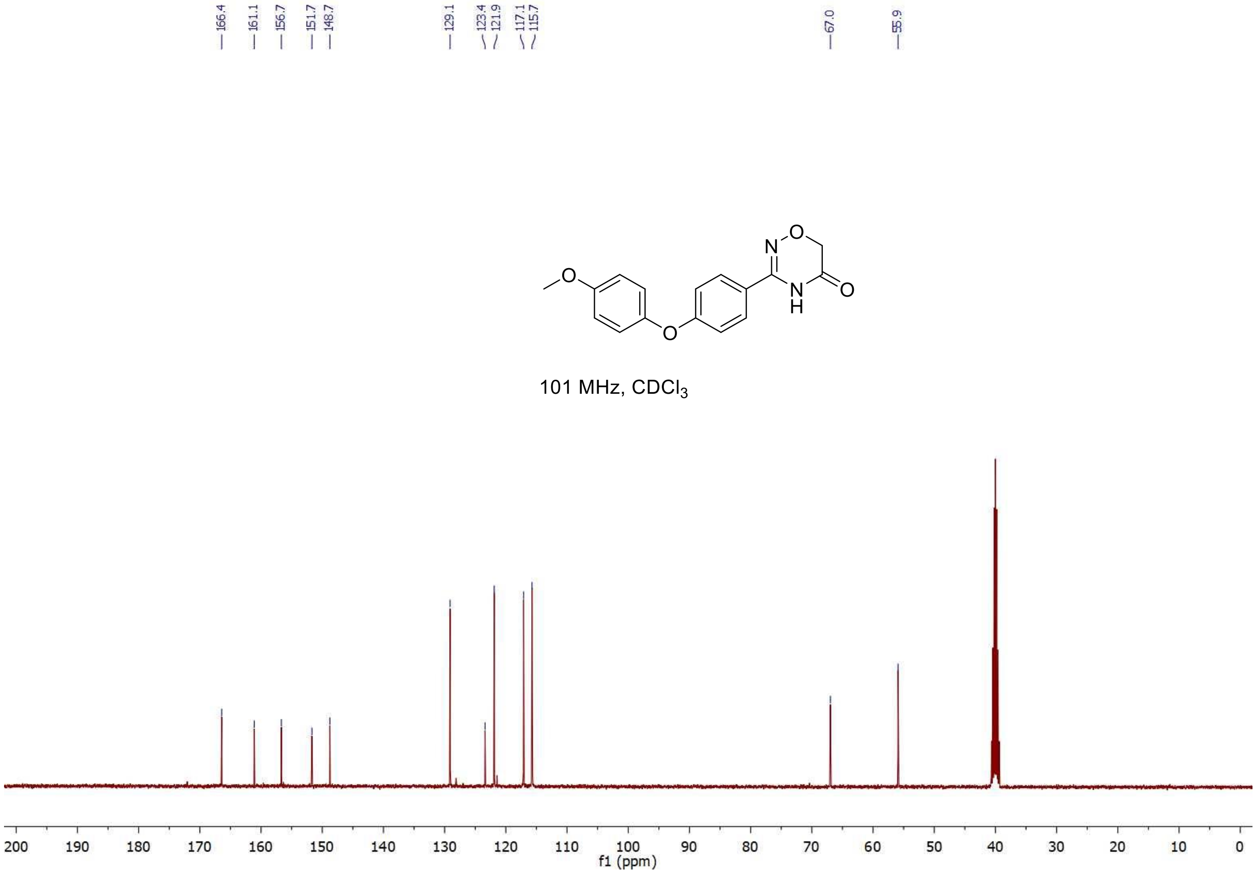

^{1}H and ^{13}C spectra of 6-methyl-3-(*p*-tolyl)-4*H*-1,2,4-oxadiazin-5(6*H*)-one (**4a**)

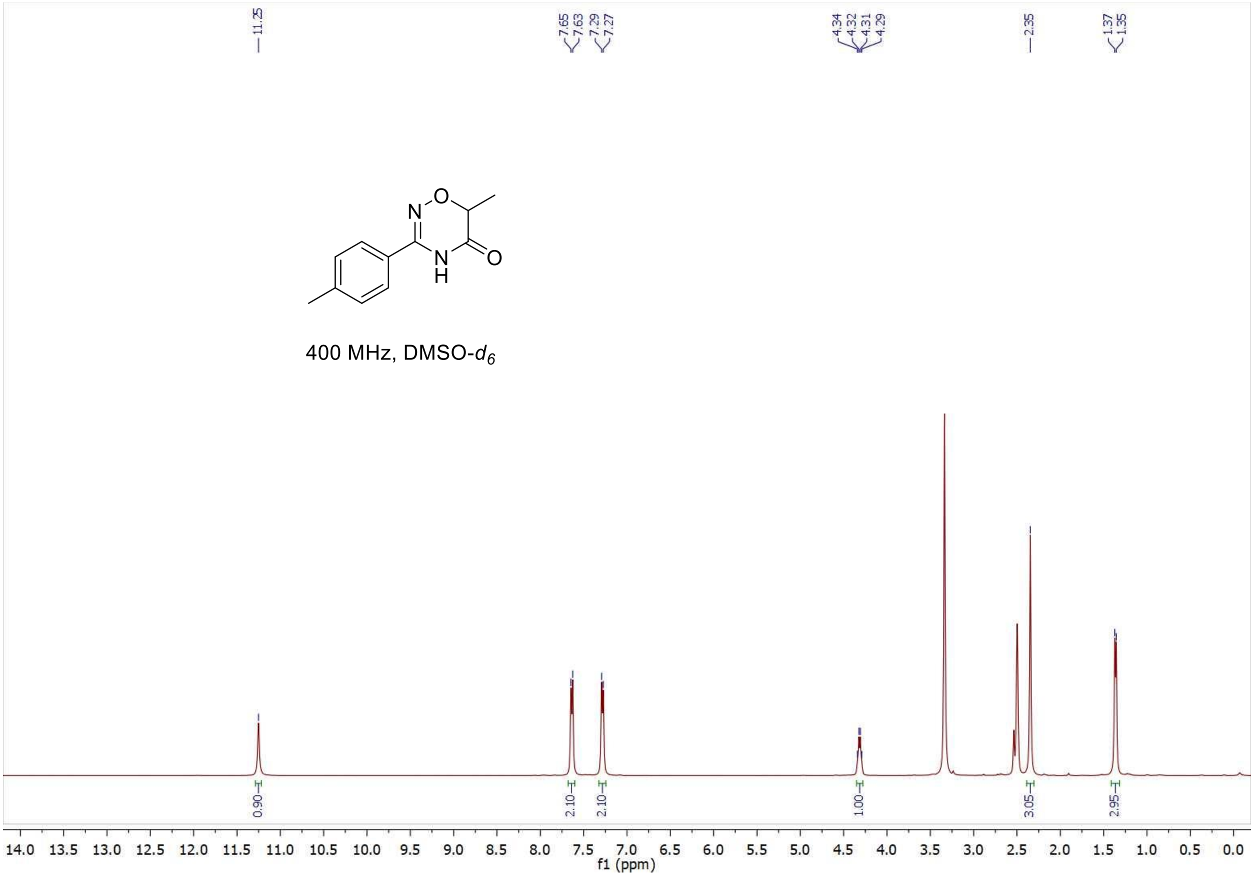

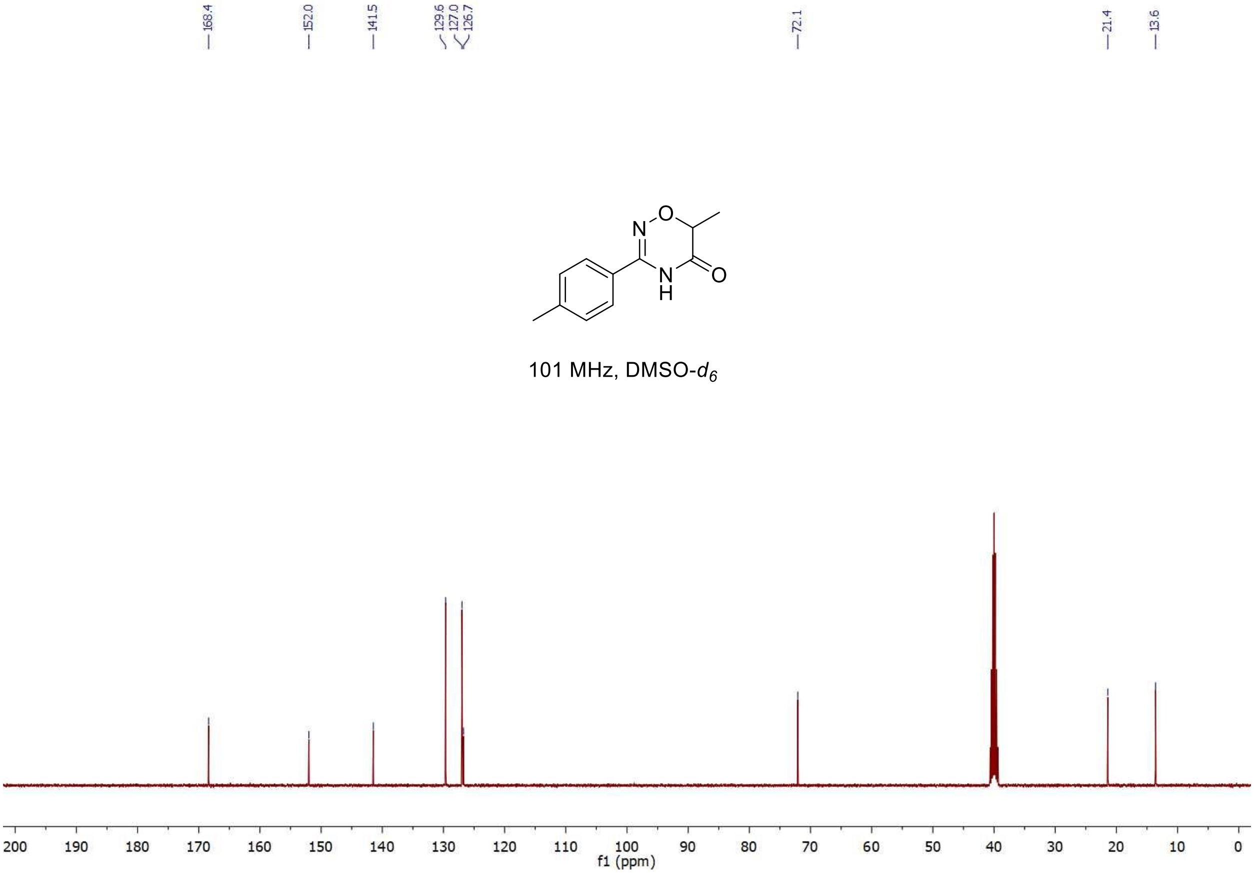

^{1}H and ^{13}C spectra of 3-(4-bromophenyl)-6-methyl-4*H*-1,2,4-oxadiazin-5(6*H*)-one (**4b**)

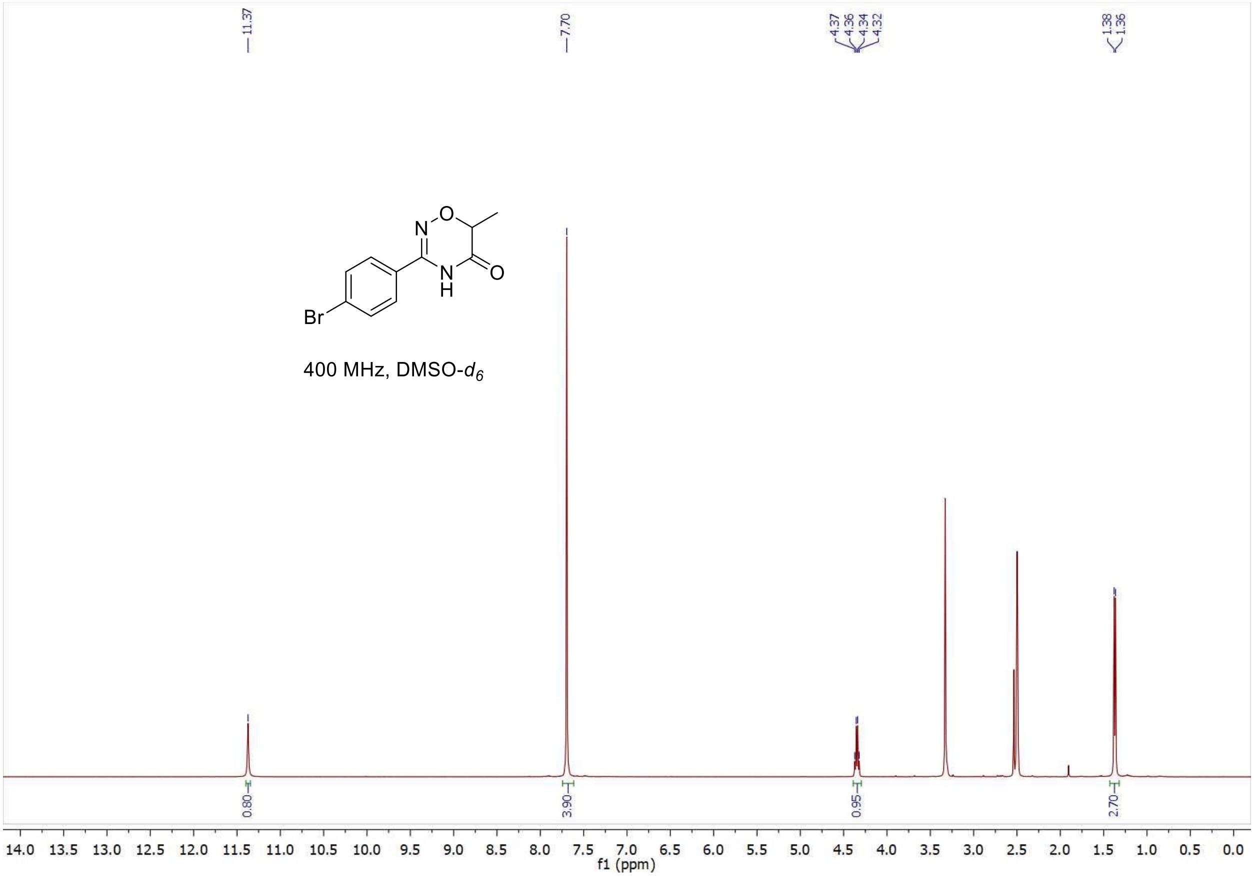

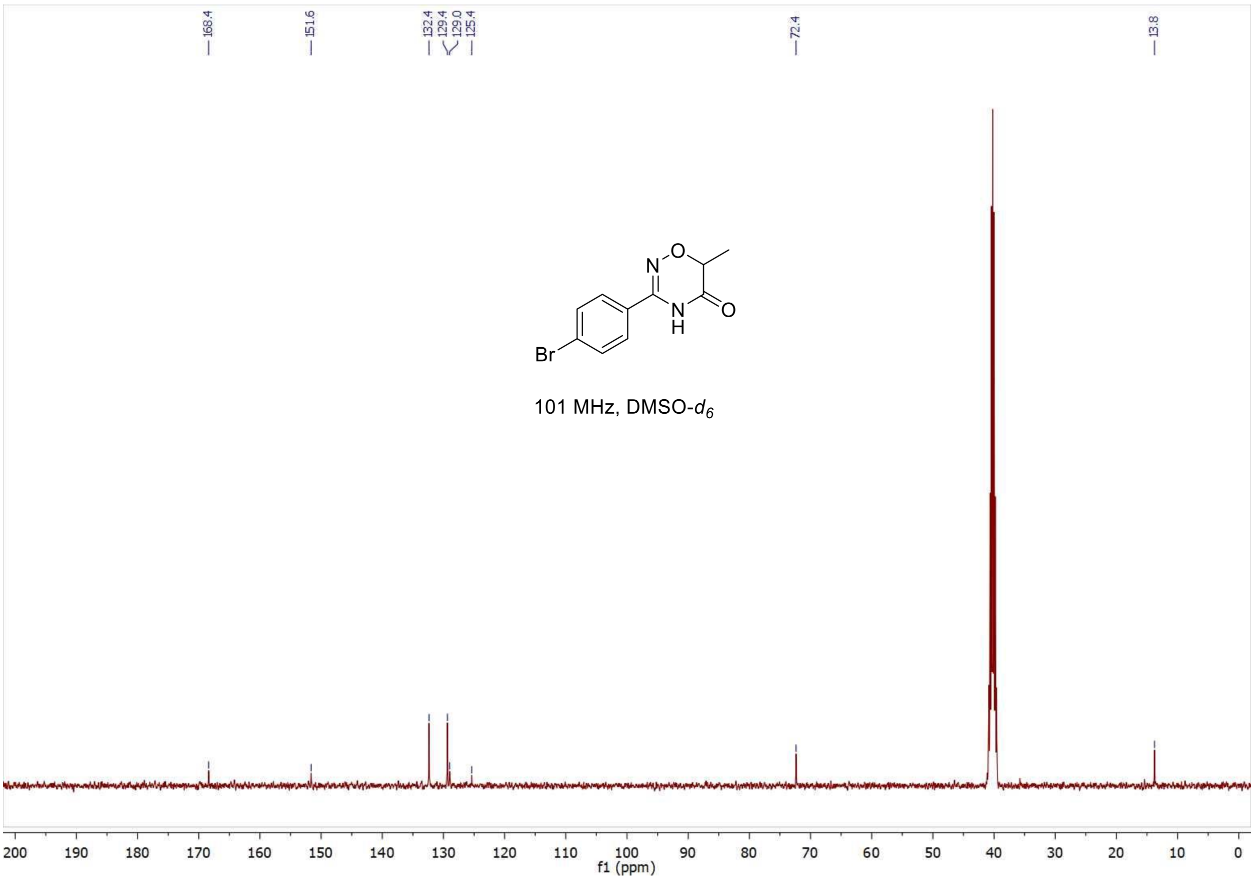

^{1}H and ^{13}C spectra of 6-methyl-3-(4-nitrophenyl)-4*H*-1,2,4-oxadiazin-5(6*H*)-one (**4c**)

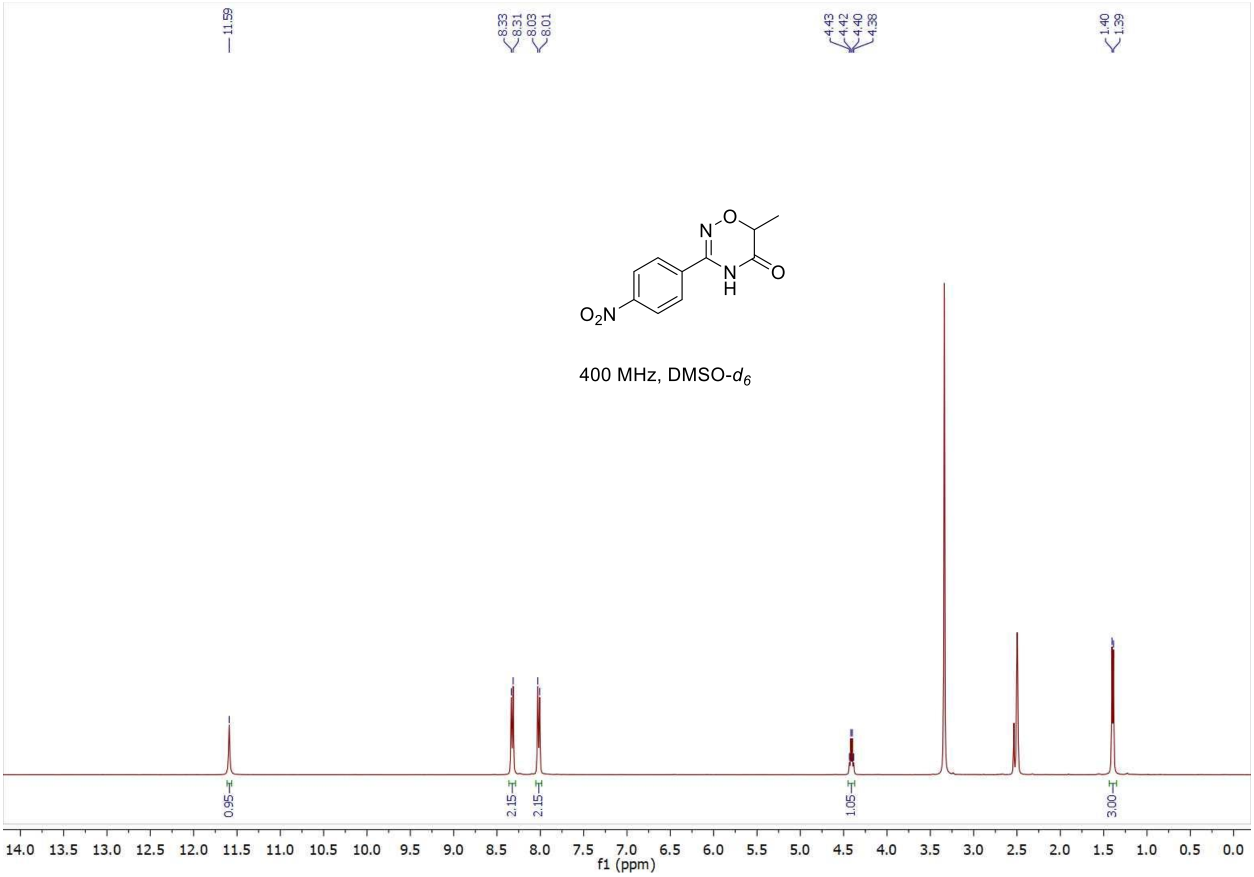

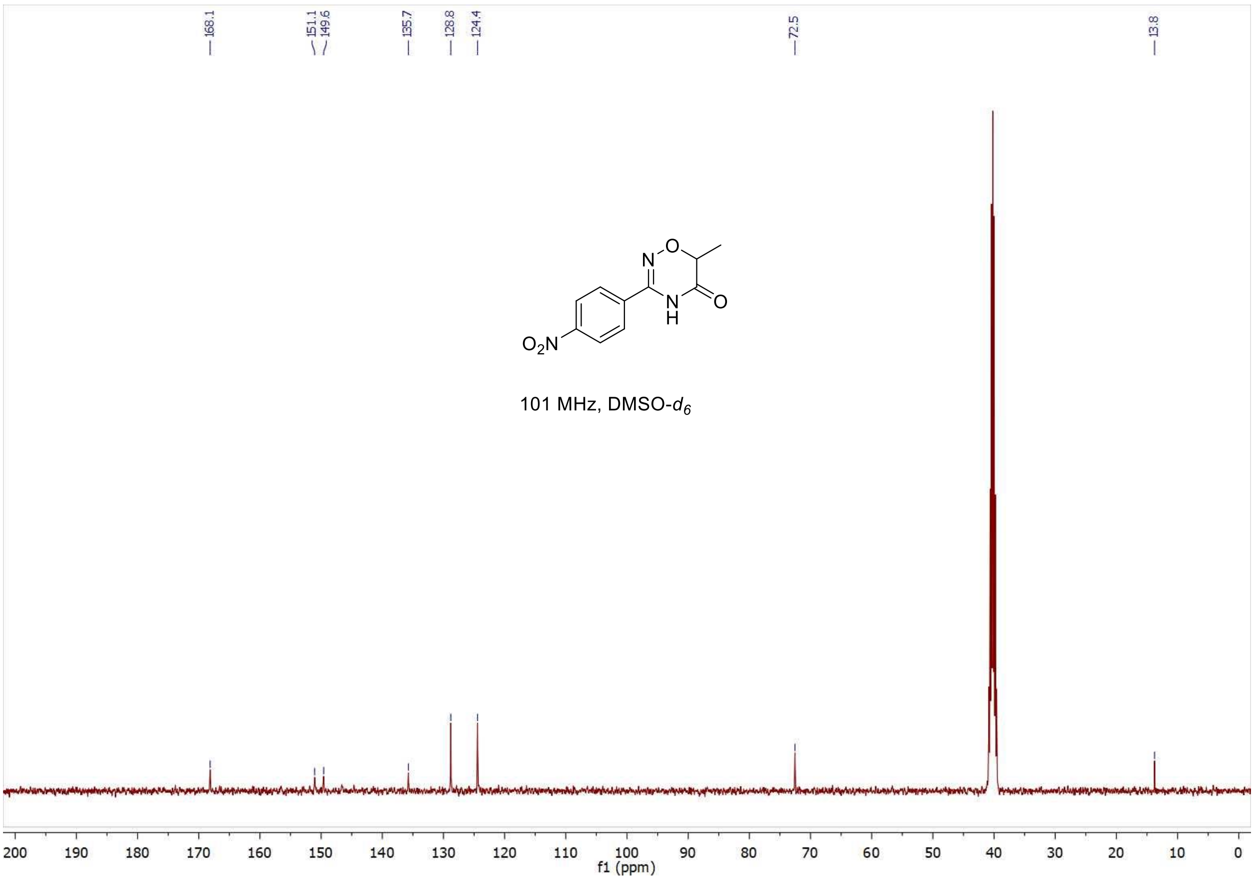

^{1}H and ^{13}C spectra of 6-methyl-3-(5-methylthiophen-2-yl)-4*H*-1,2,4-oxadiazin-5(6*H*)-one (**4d**)

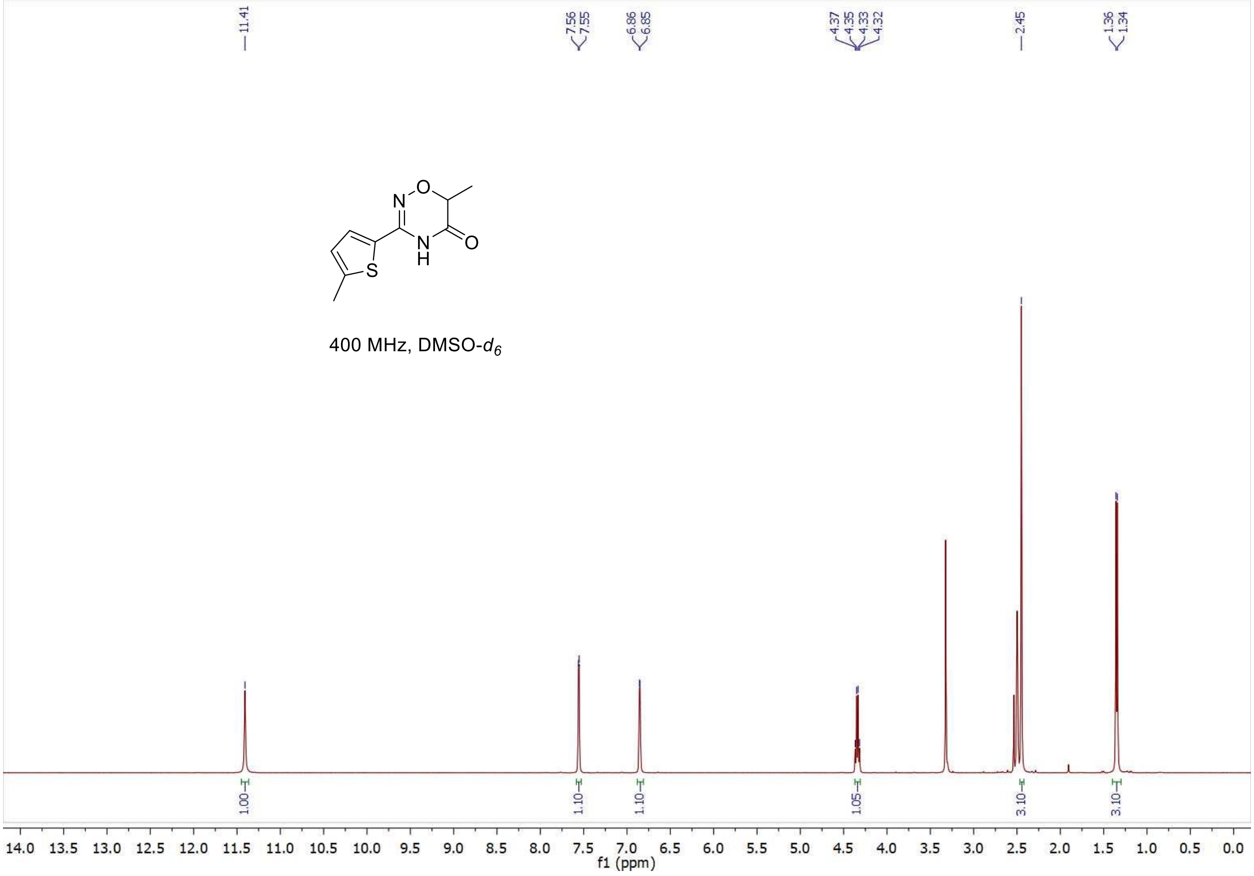

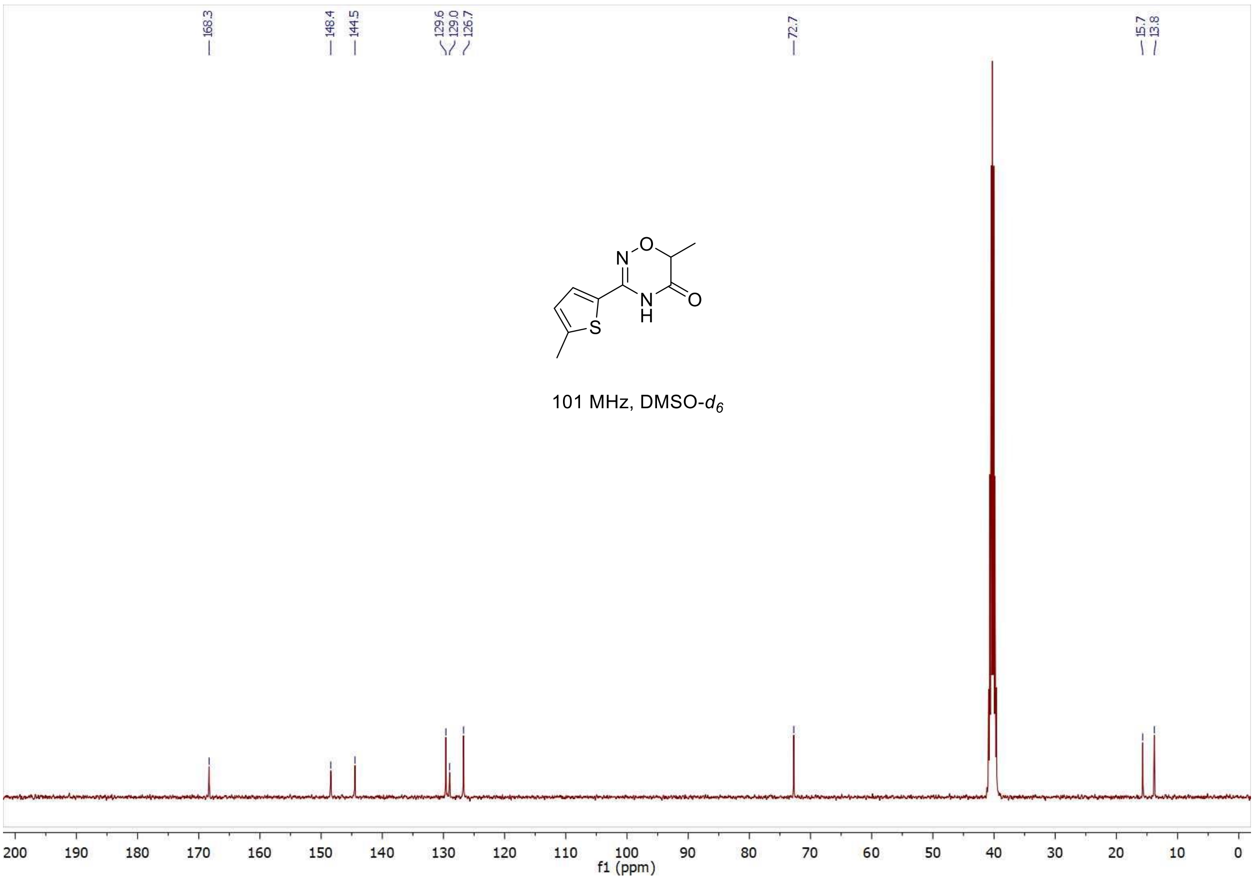

^{1}H and ^{13}C spectra of 3-(4-methoxyphenyl)-6-methyl-4*H*-1,2,4-oxadiazin-5(6*H*)-one (**4e**)

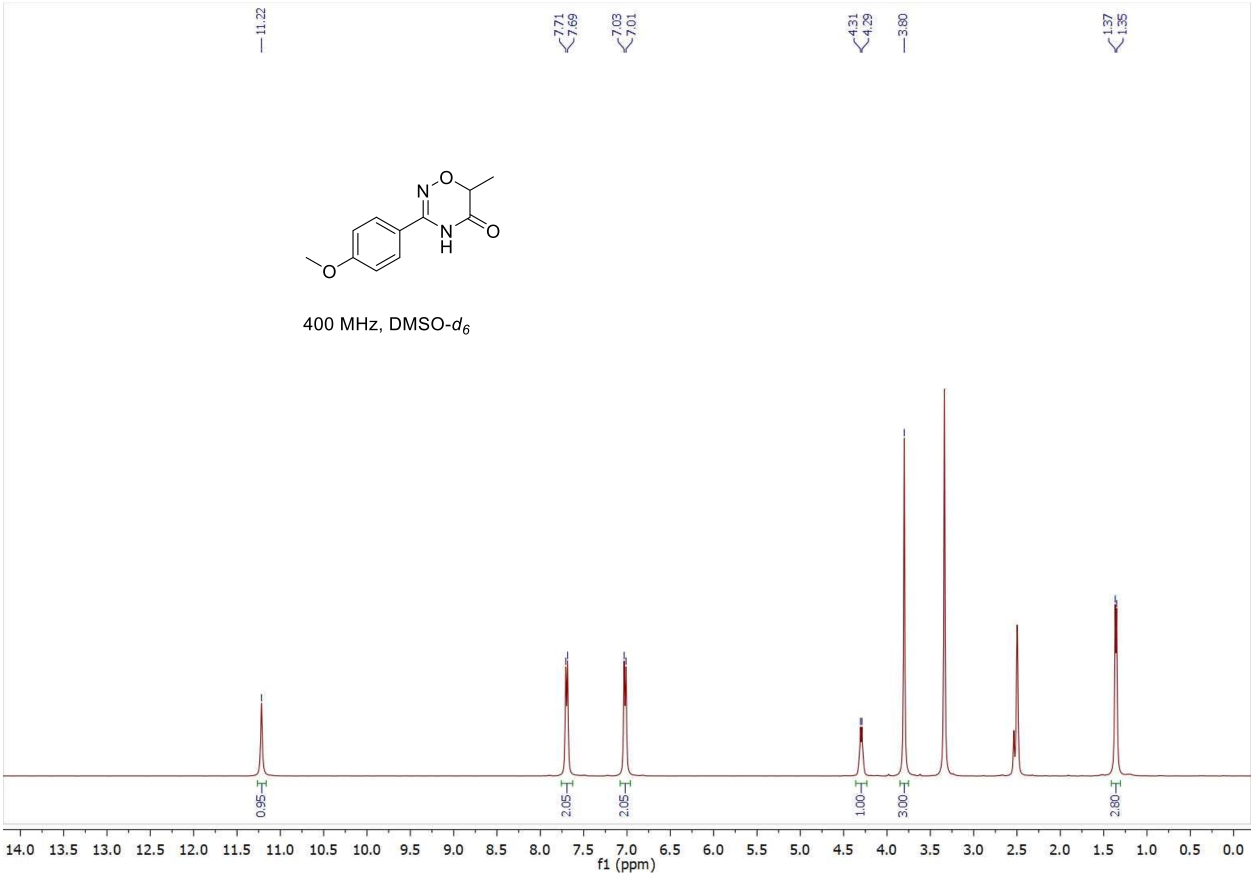

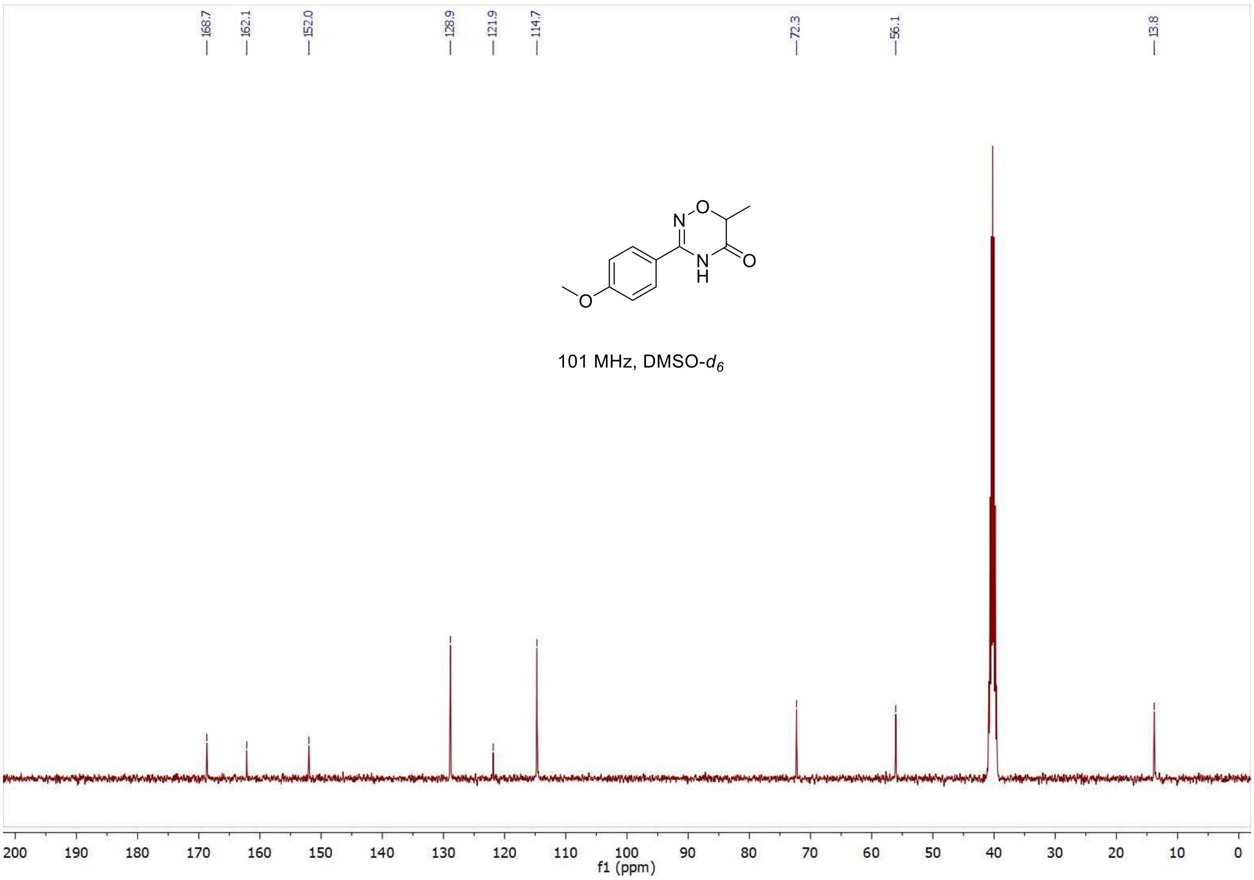

^{1}H and ^{13}C spectra of 6-ethyl-3-(*p*-tolyl)-4*H*-1,2,4-oxadiazin-5(6*H*)-one (**5a**)

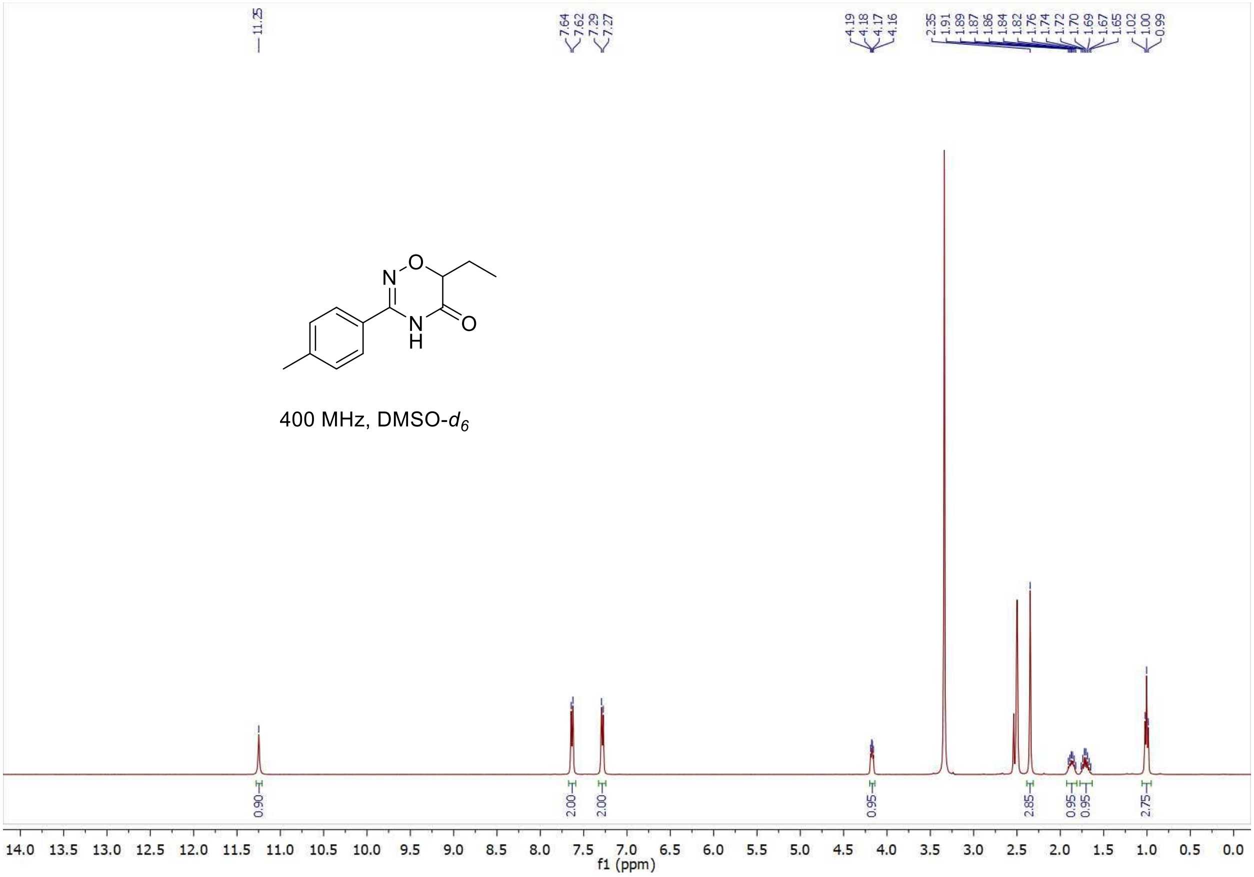

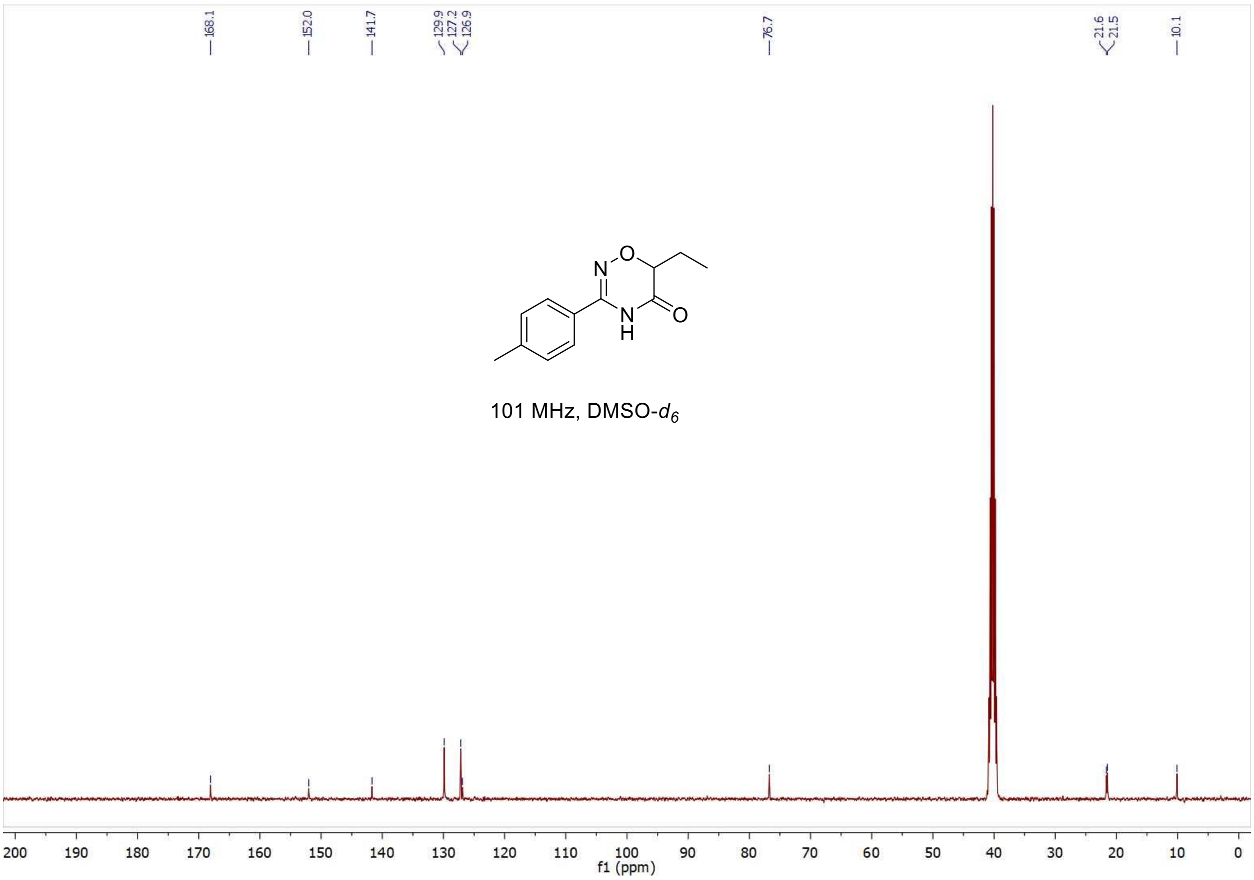

^{1}H and ^{13}C spectra of 3-(4-bromophenyl)-6-ethyl-4*H*-1,2,4-oxadiazin-5(6*H*)-one (**5b**)

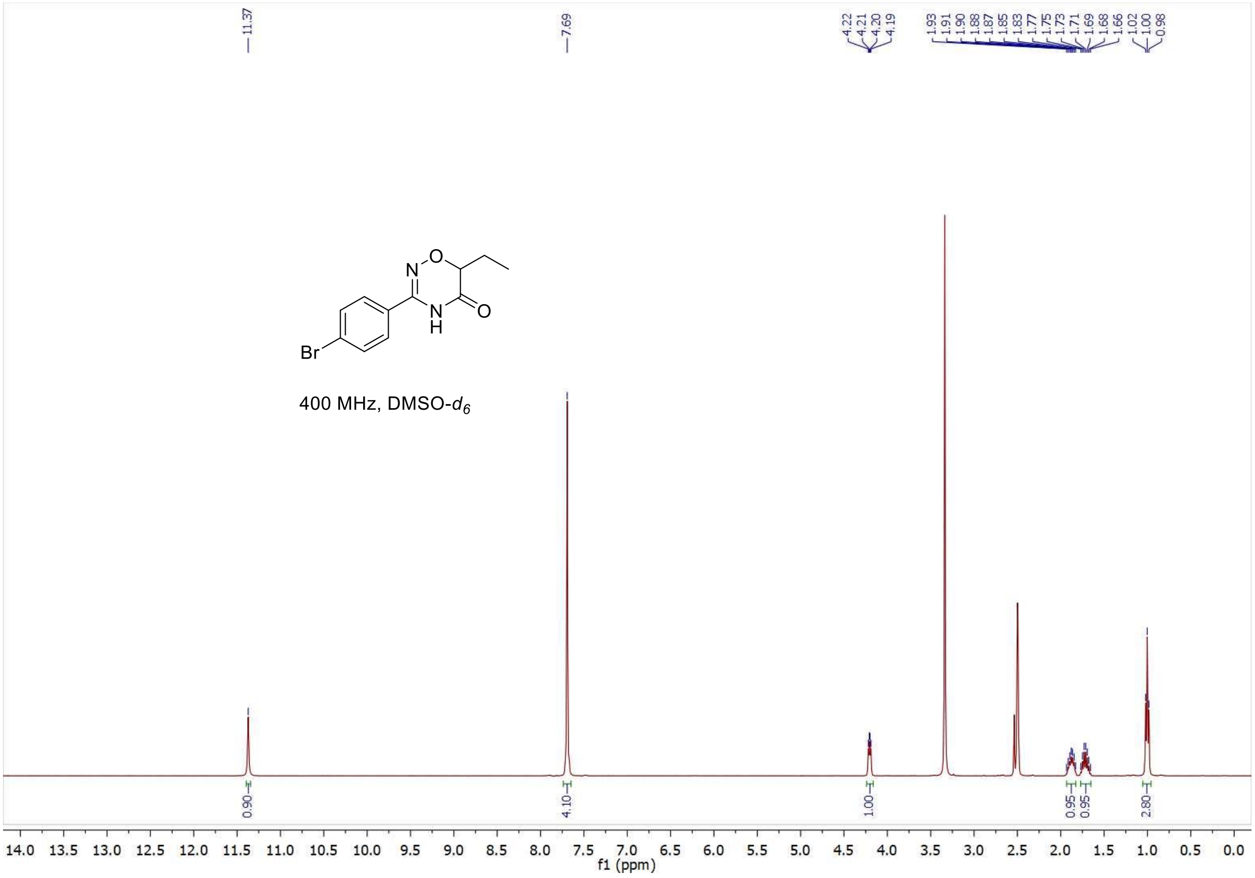

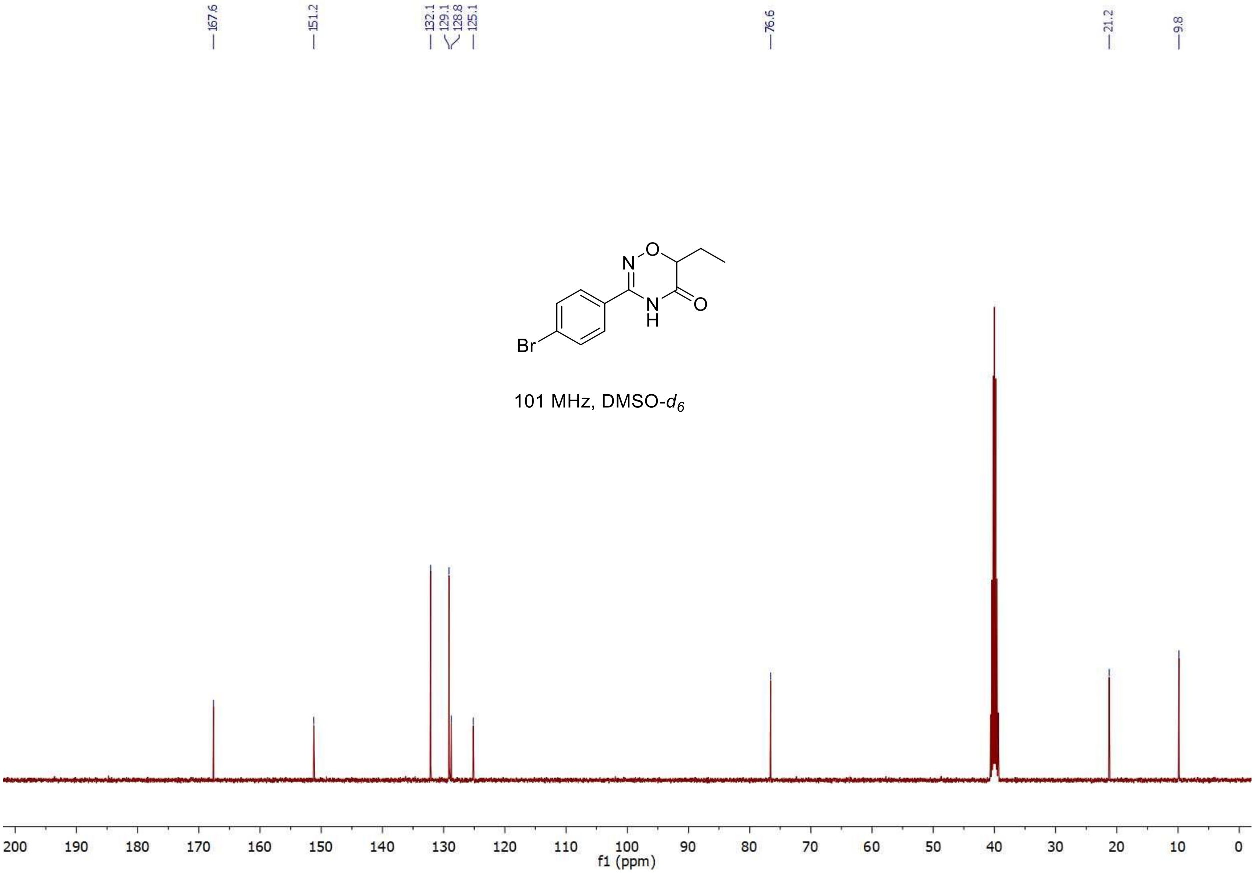

^{1}H and ^{13}C spectra of 6-ethyl-3-(4-nitrophenyl)-4*H*-1,2,4-oxadiazin-5(6*H*)-one (**5c**)

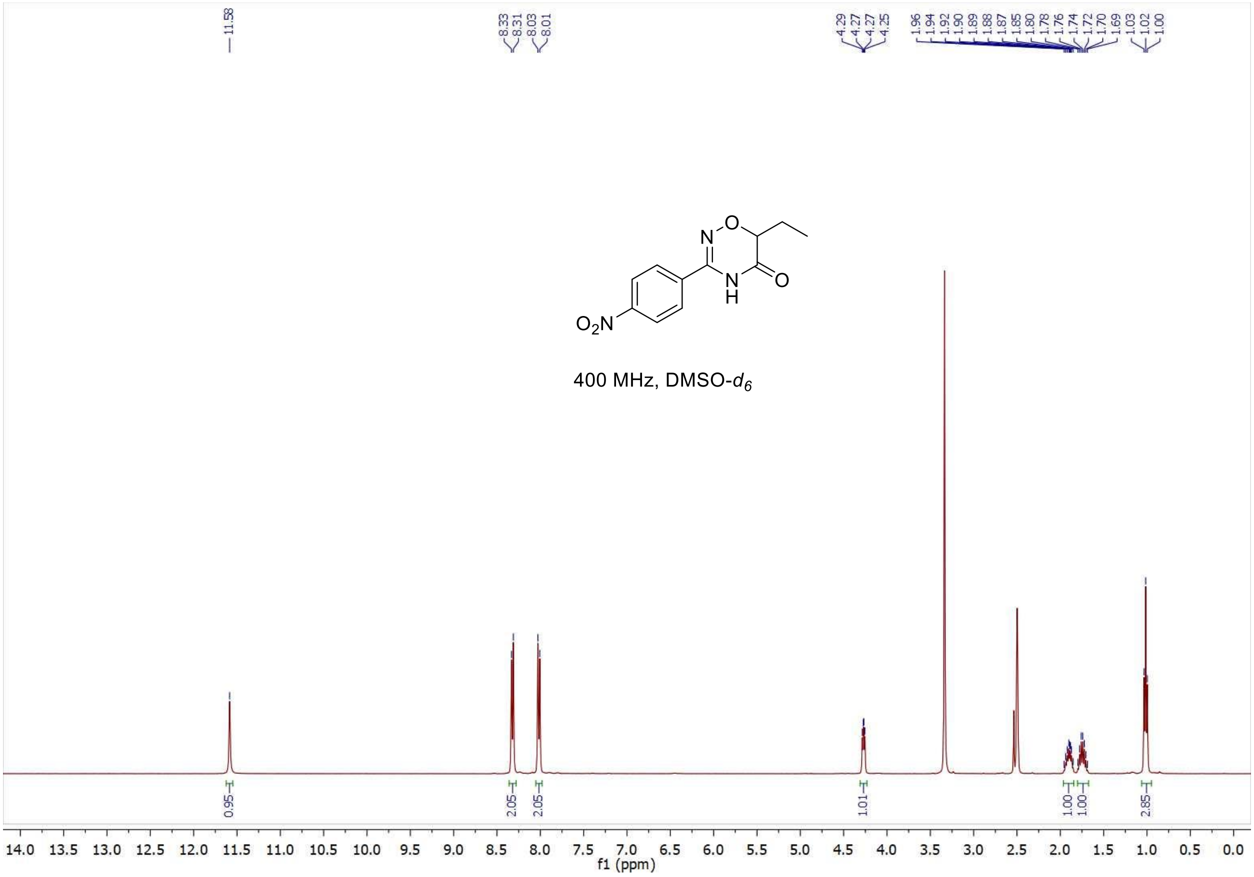

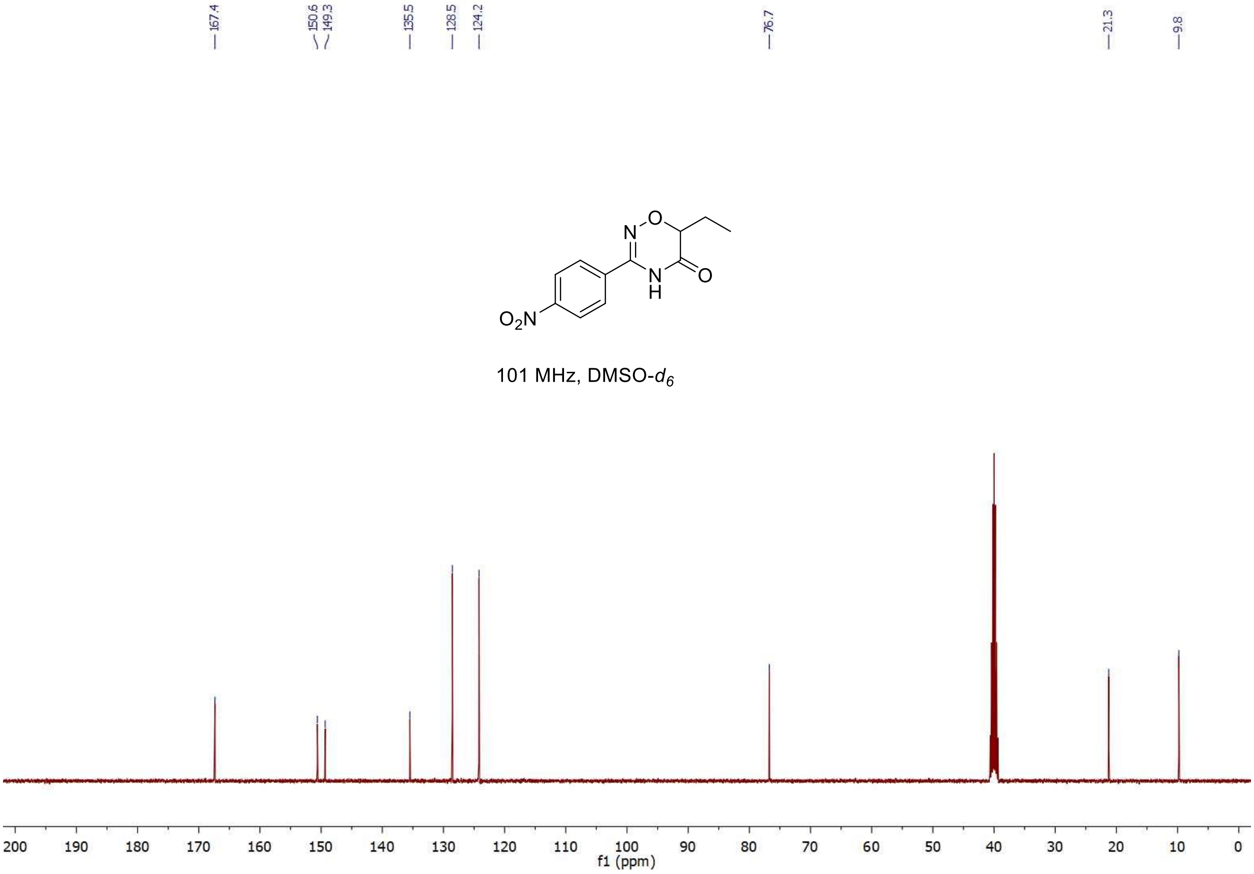

^{1}H and ^{13}C spectra of 6-ethyl-3-(5-methylthiophen-2-yl)-4*H*-1,2,4-oxadiazin-5(6*H*)-one (**5d**)

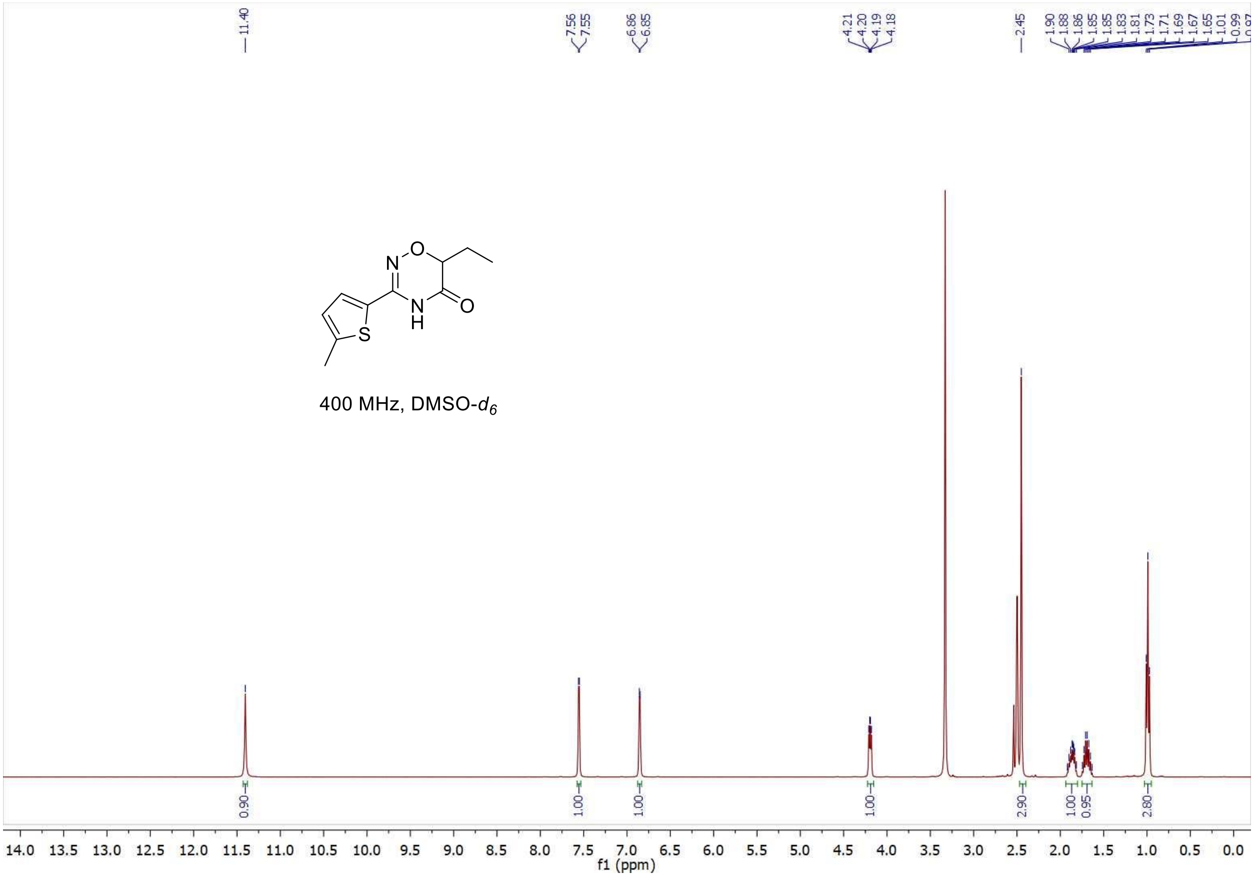

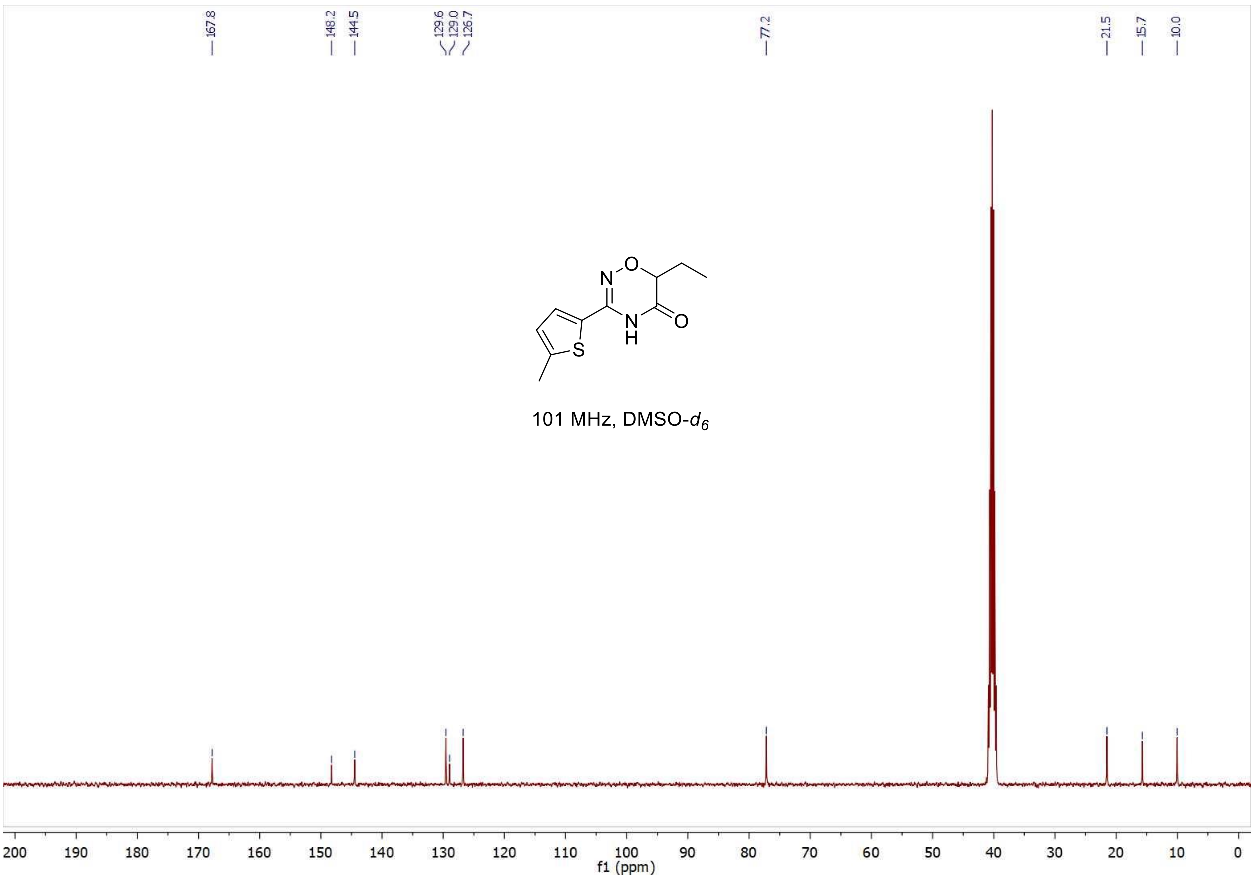

^{1}H and ^{13}C spectra of 6-ethyl-3-(4-methoxyphenyl)-4*H*-1,2,4-oxadiazin-5(6*H*)-one (**5e**)

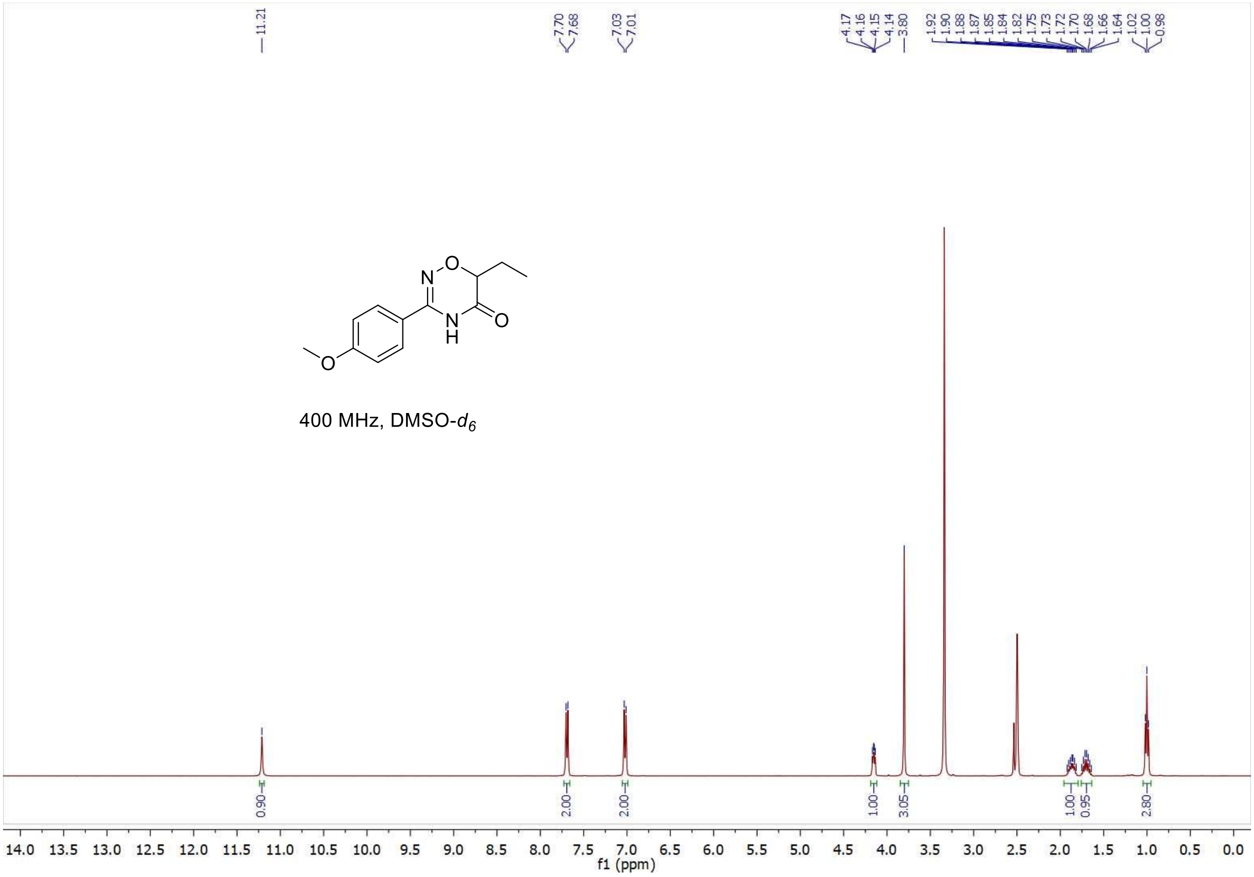

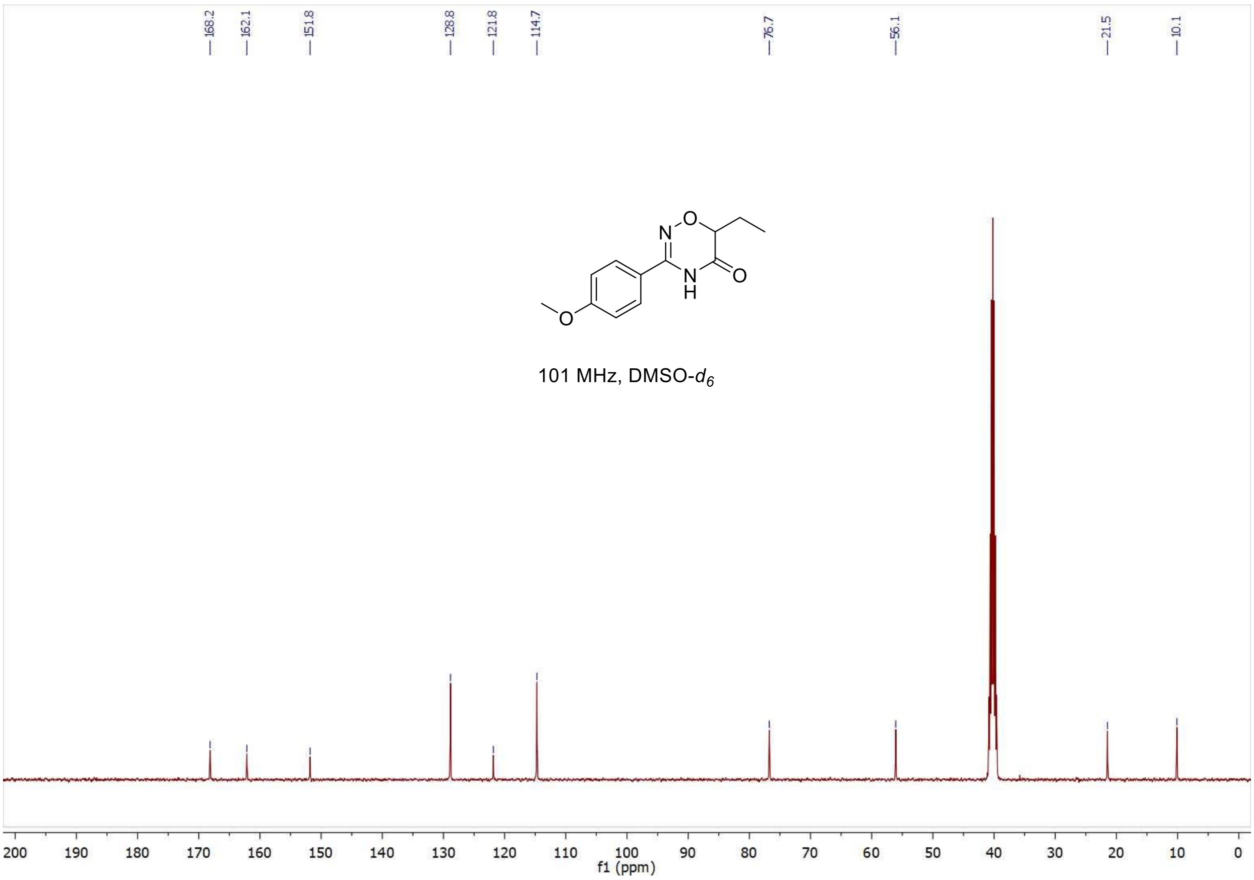

^{1}H and ^{13}C spectra of 6-ethyl-3-(4-(4-methoxyphenoxy)phenyl)-4*H*-1,2,4-oxadiazin-5(6*H*)-one (**5f**)

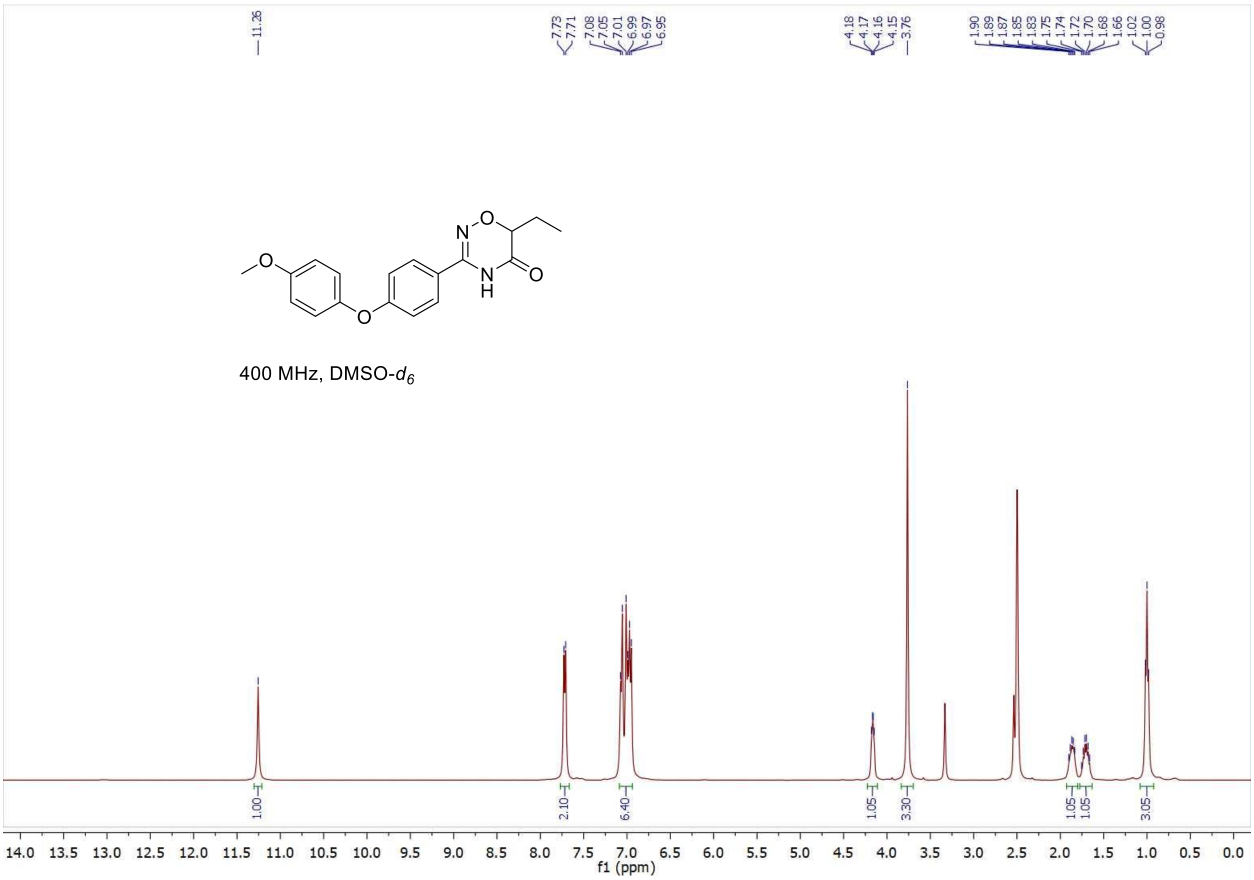

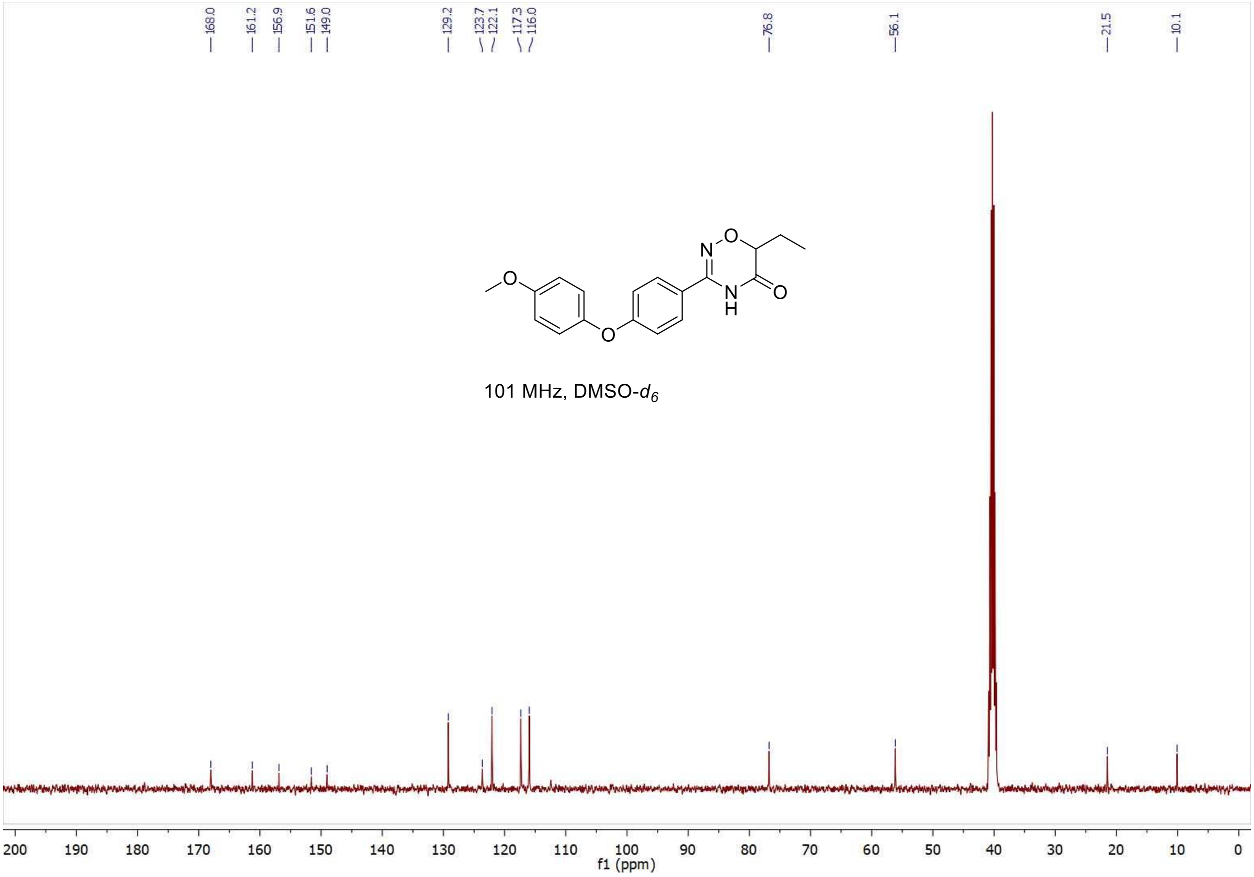

^{1}H and ^{13}C spectra of 6-ethyl-3-phenyl-4*H*-1,2,4-oxadiazin-5(6*H*)-one (**5g**)

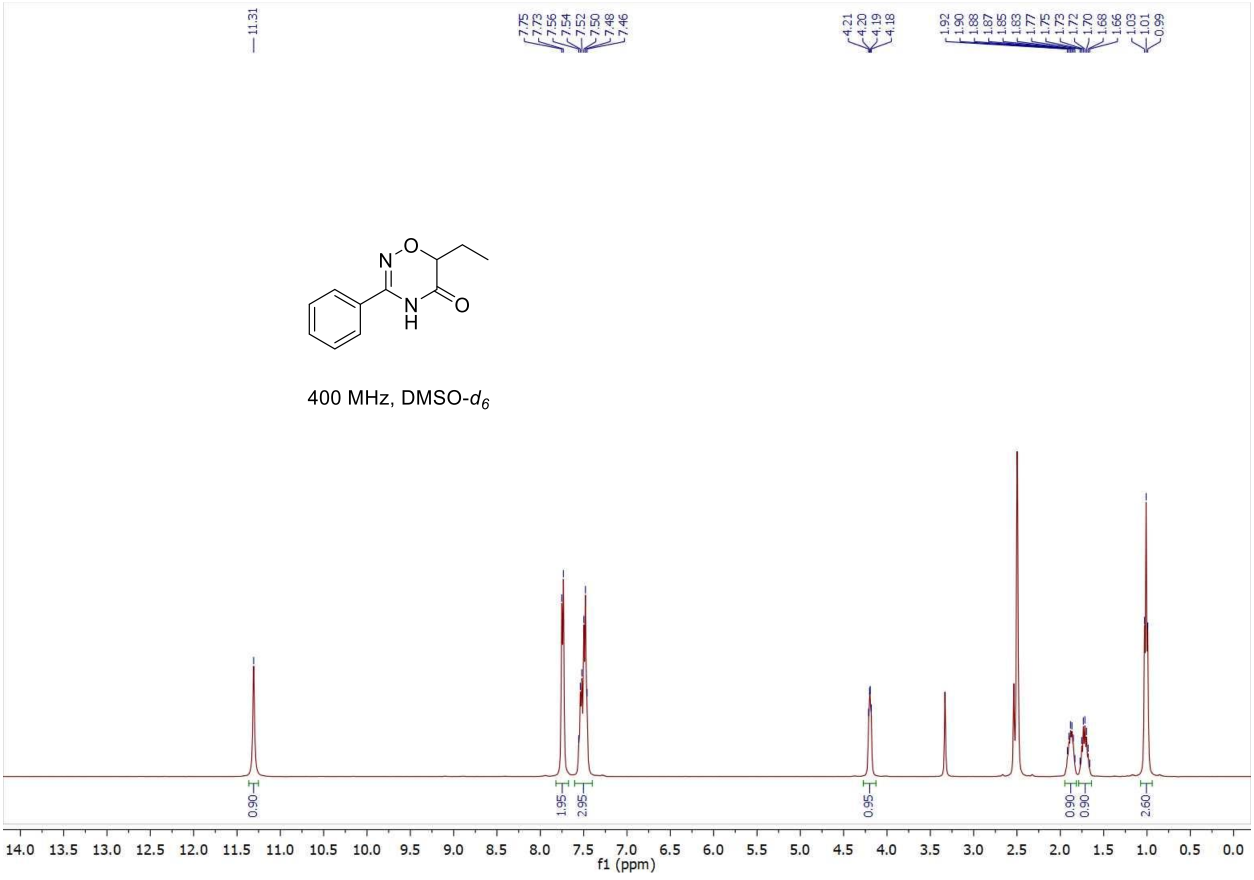

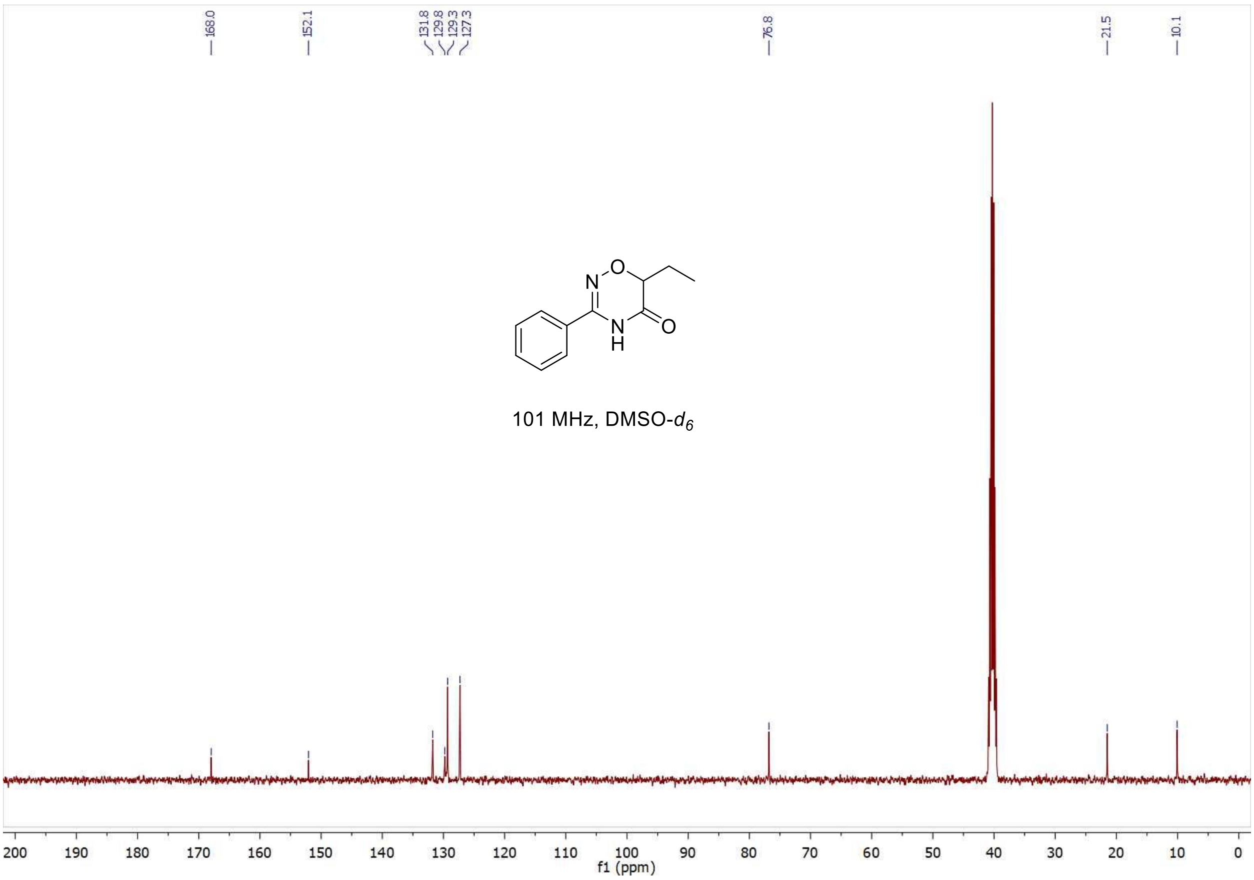

^{1}H, ^{13}C and ^{19}F spectra of 6-ethyl-3-(4-fluorophenyl)-4*H*-1,2,4-oxadiazin-5(6*H*)-one (**5h**)

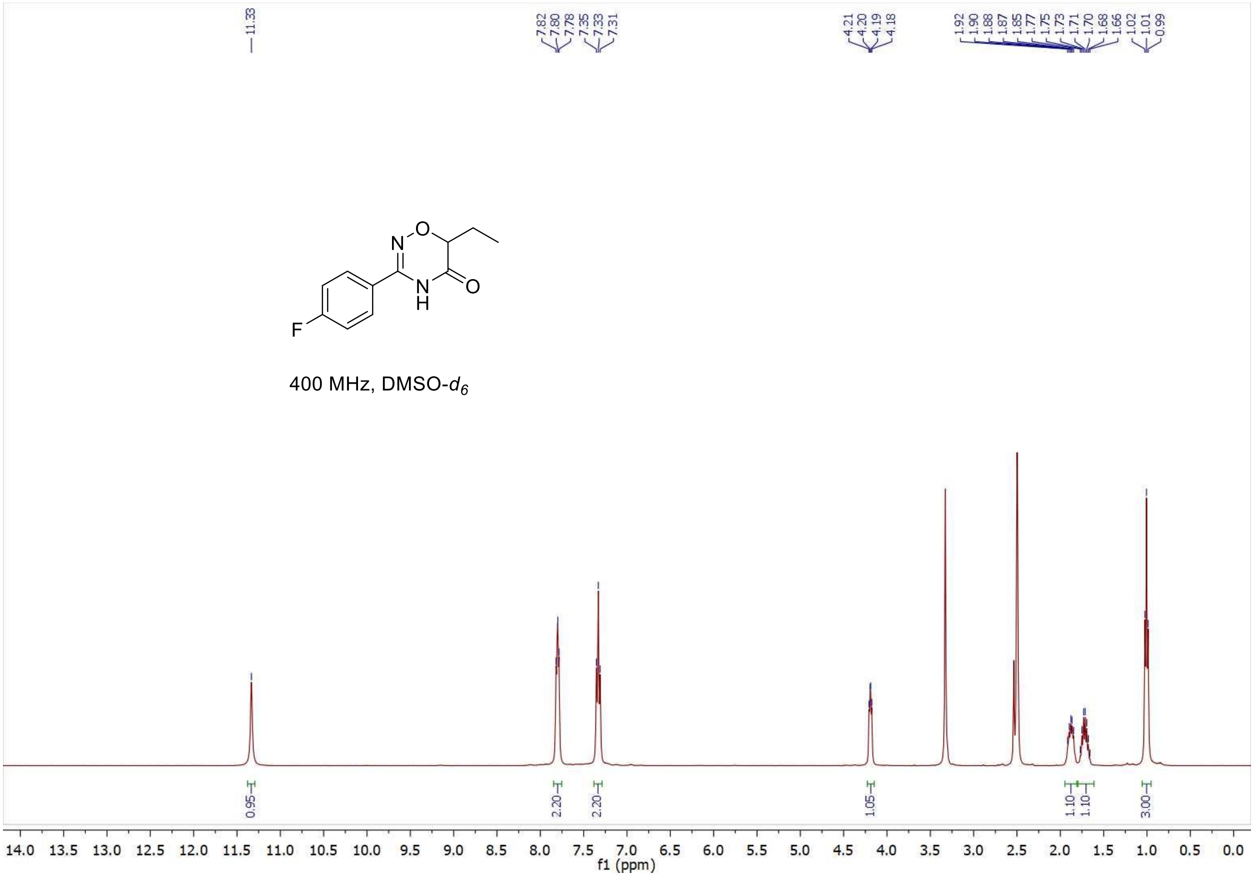

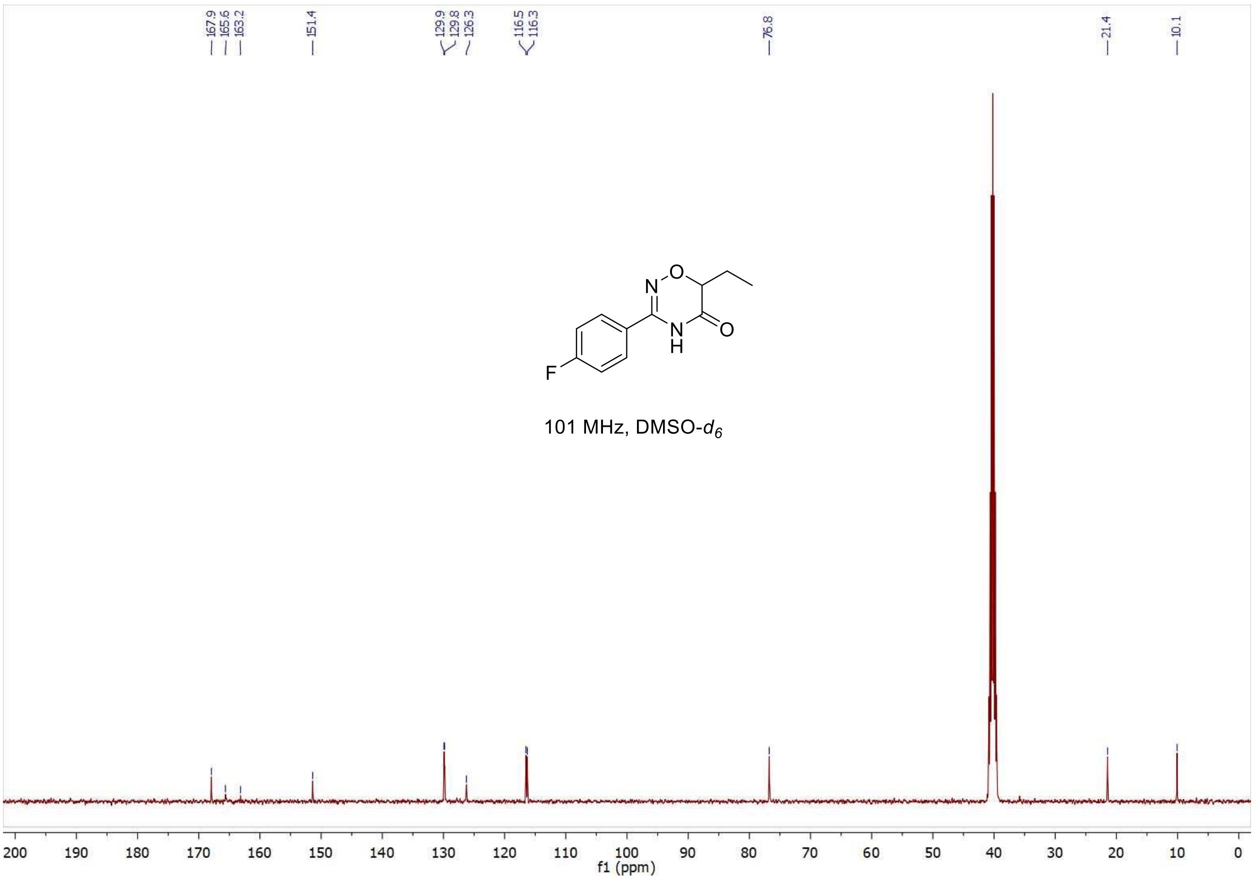

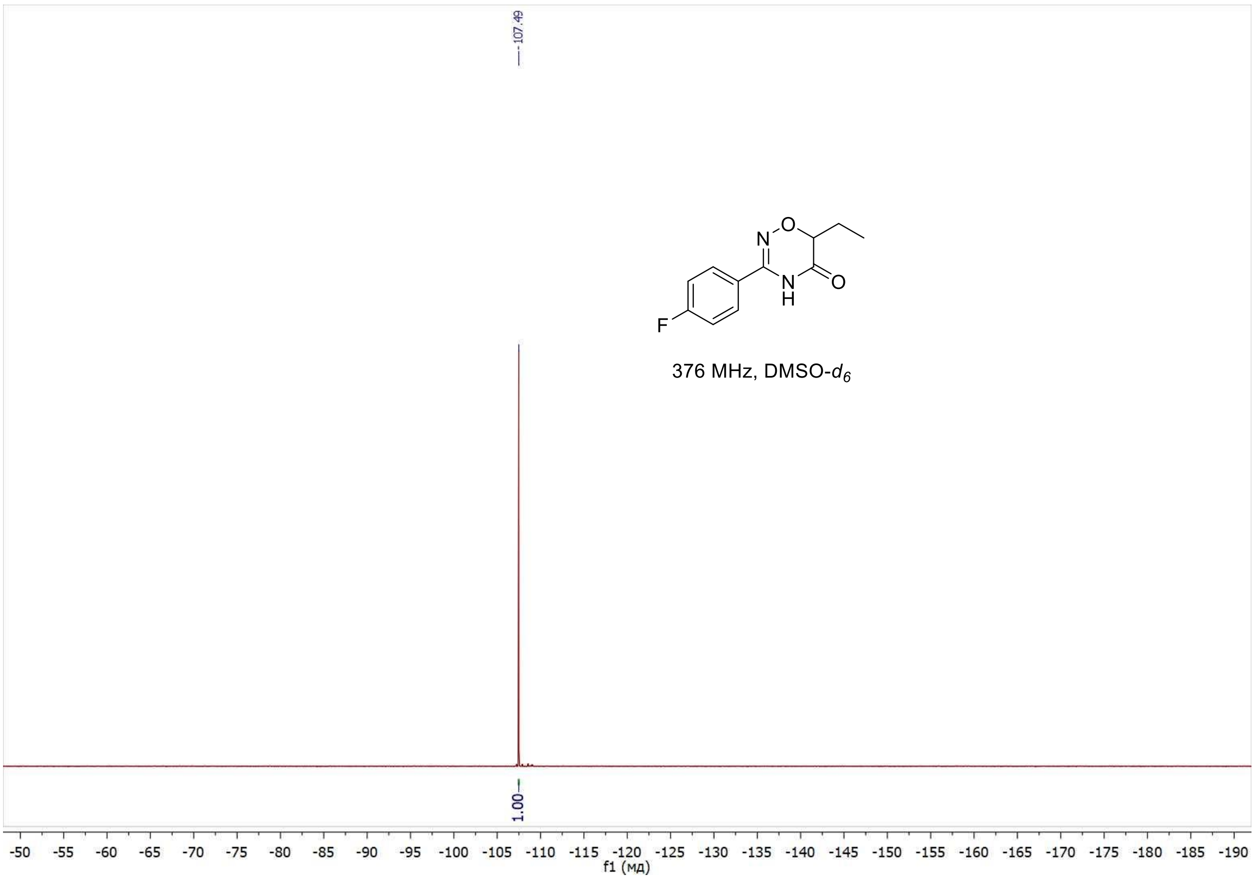

-107.49
376 MHz, DMSO-d6
1.00
-50 -55 -60 -65 -70 -75 -80 -85 -90 -95 -100 -105 -110 -115 -120 -125 -130 -135 -140 -145 -150 -155 -160 -165 -170 -175 -180 -185 -190
f1 (мд)

^{1}H and ^{13}C spectra of 3-benzyl-6-ethyl-4*H*-1,2,4-oxadiazin-5(6*H*)-one (**5i**)

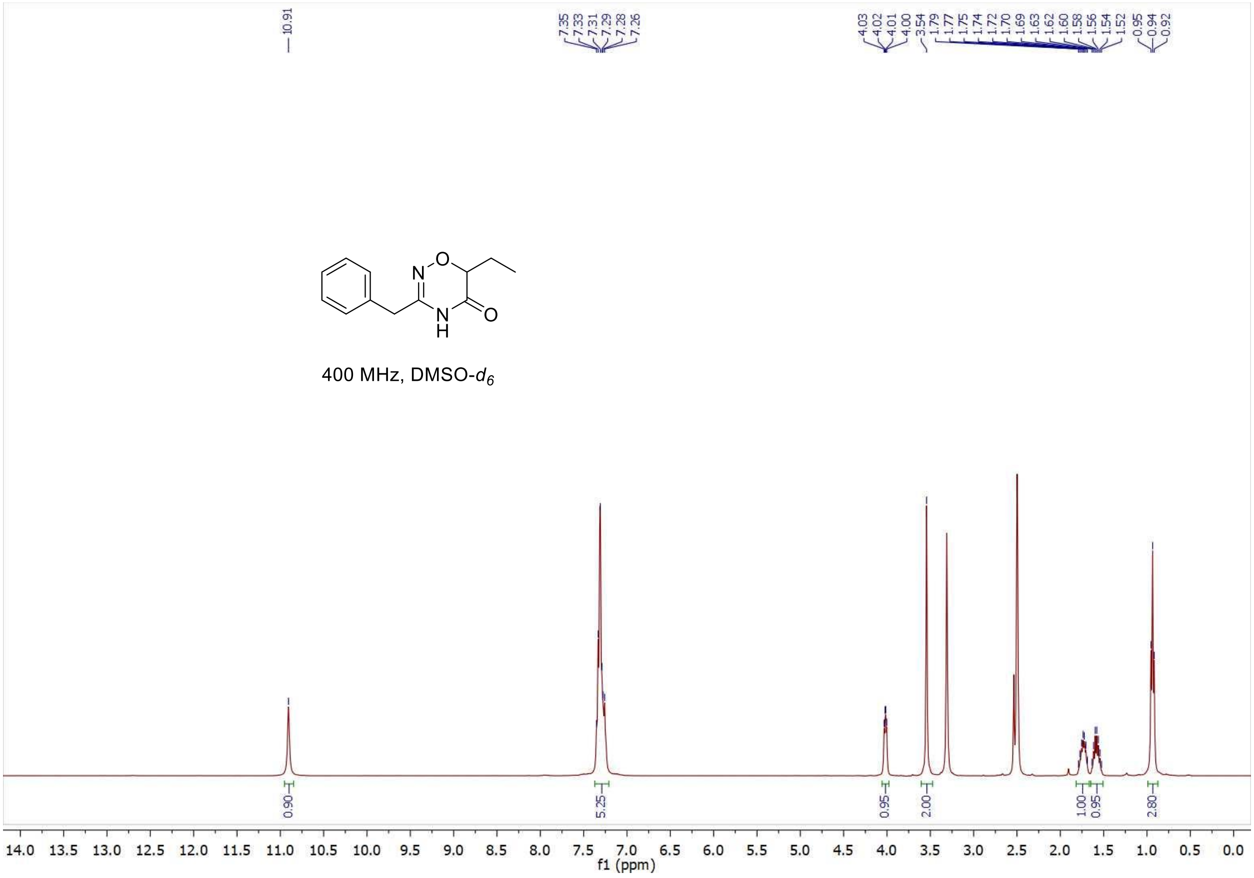

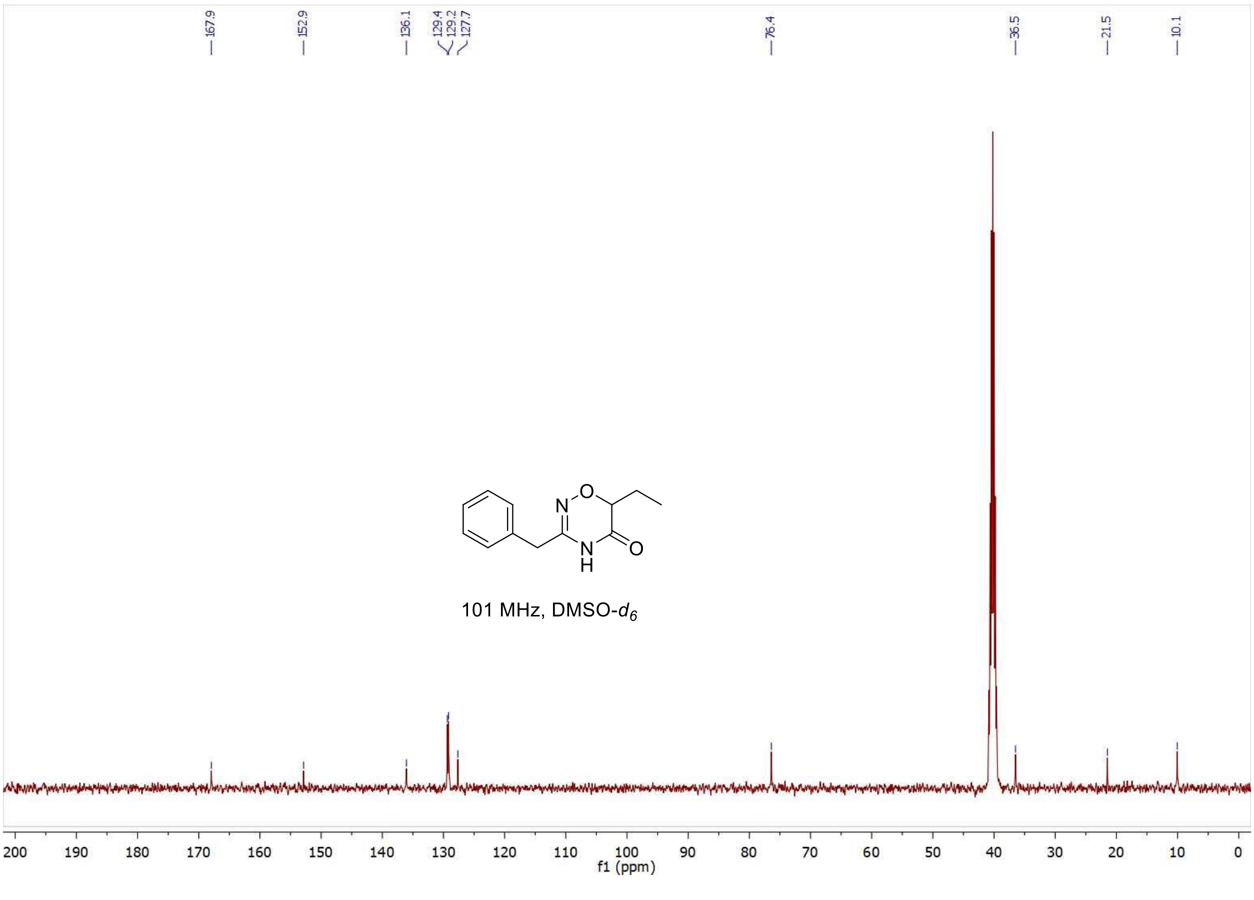

^{1}H and ^{13}C spectra of 2-(3-(3-chlorophenyl)-5-oxo-5,6-dihydro-4*H*-1,2,4-oxadiazin-6-yl)acetic acid (**8b**)

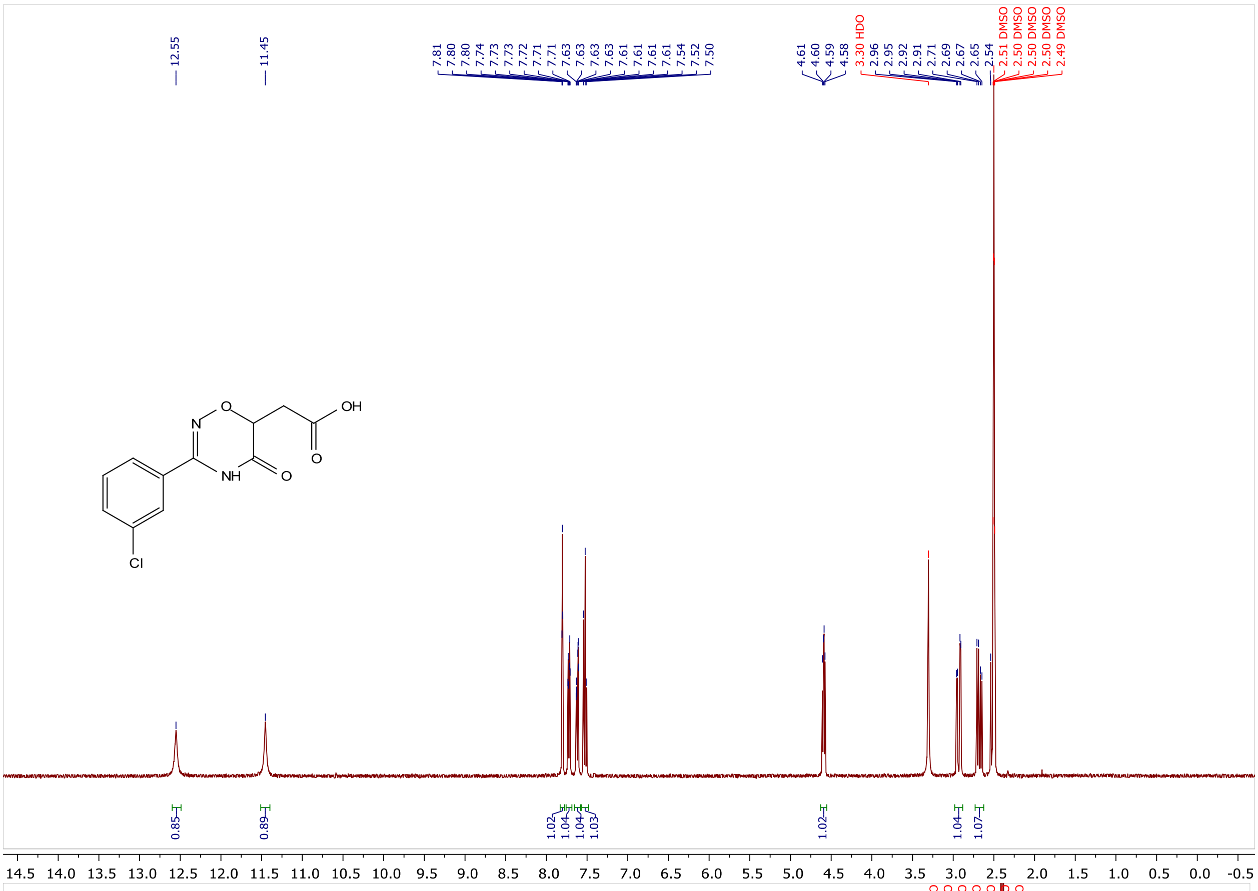

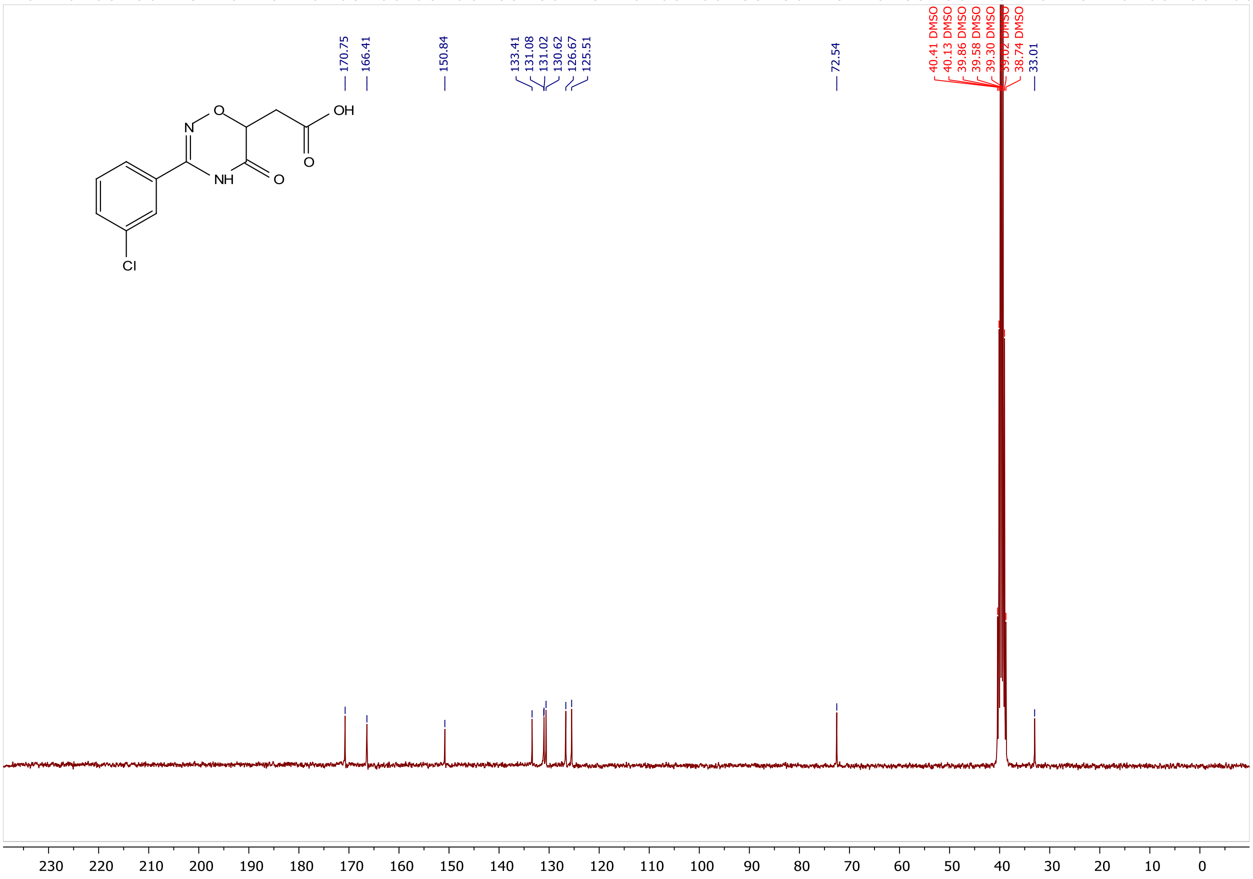

^{1}H and ^{13}C spectra of 2-(5-oxo-3-(4-(trifluoromethoxy)phenyl)-5,6-dihydro-4*H*-1,2,4-oxadiazin-6-yl)acetic acid (**8c**)

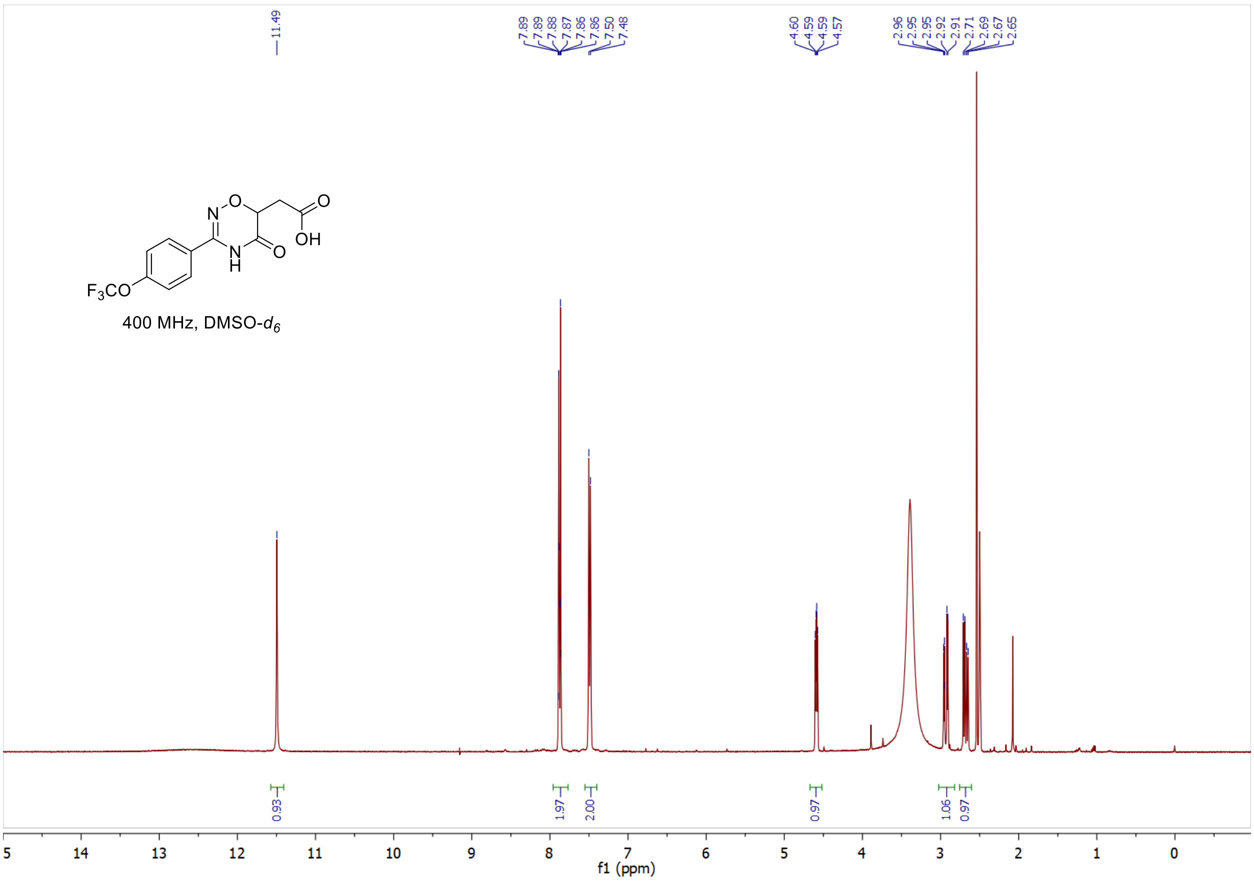

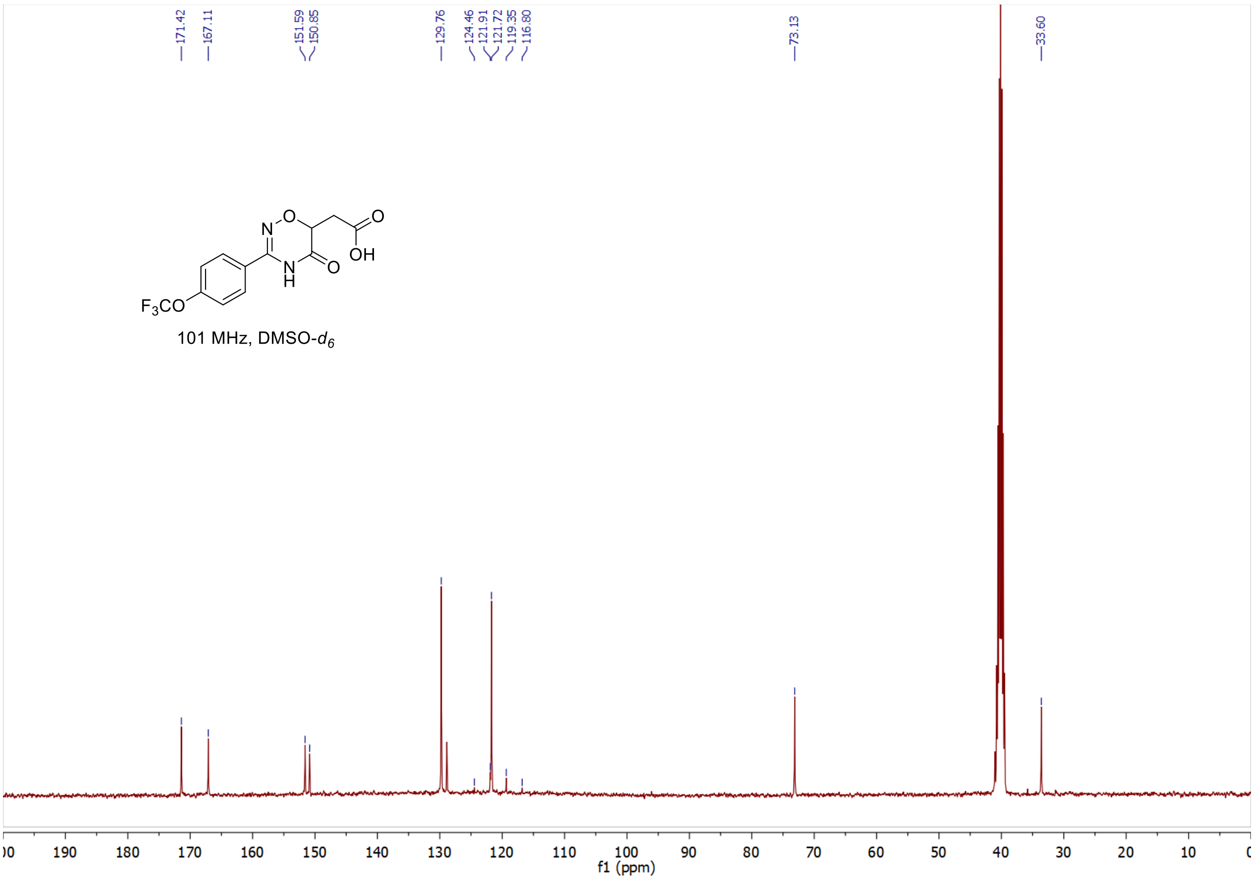

S2. X–ray diffraction data for compounds 3a and 5b

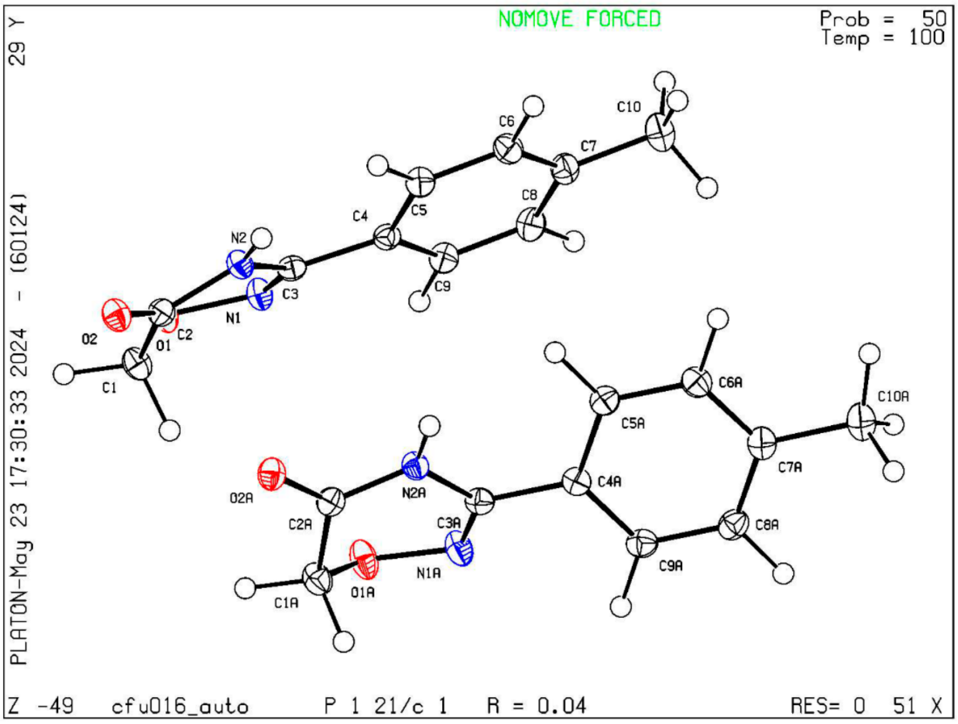

Figure S1. The molecular structure of **3a**. Thermal ellipsoids are drawn at the 50% probability level.

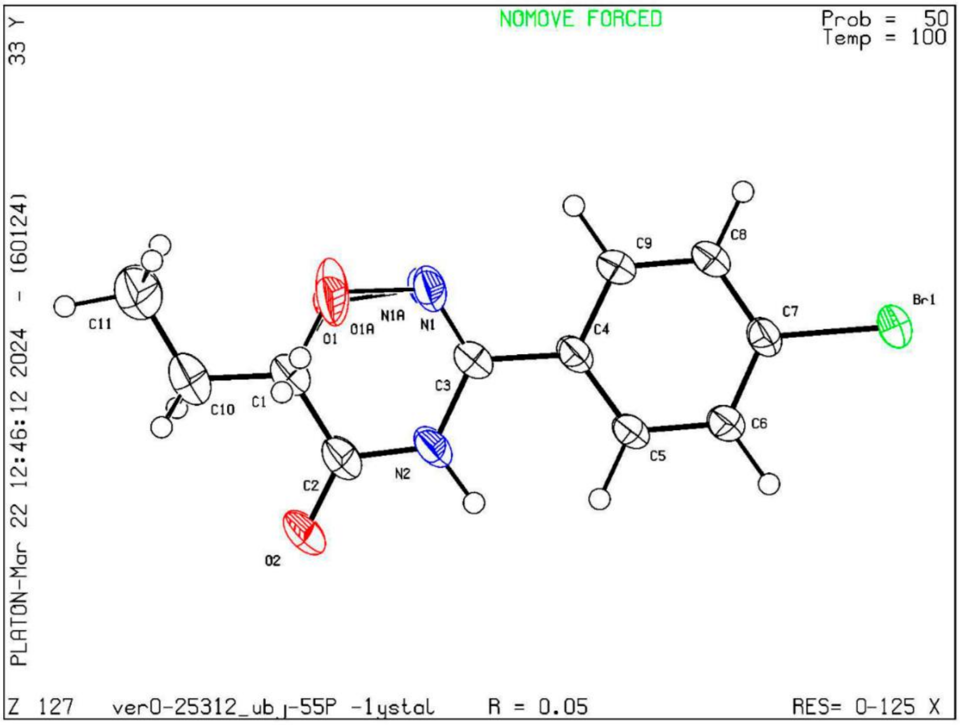

Figure S2. The molecular structure of **5b**. Thermal ellipsoids are drawn at the 50% probability level.

Table S1. Crystal data and structure refinement parameters for **3a** and **5b**.

Compound	**3a**	**5b**
Identification code	CFU-016	CJL-550
CCDC number	2376673	2376674
Empirical formula	$C_{20}H_{20}N_4O_4$	$C_{11}H_{11}BrN_2O_2$
Formula weight	380.40	283.13
Temperature, K	100(2)	100(2)
Crystal system	monoclinic	triclinic
Space group	$P2_1/c$	P-1
a, Å	8.05820(10)	5.6030(2)
b, Å	7.04220(10)	9.0736(5)
c, Å	31.8313(5)	11.6421(4)
α, °	90	84.502(4)
β, °	95.2110(10)	79.054(3)
γ, °	90	74.316(4)
Volume, $Å^3$	1798.88(4)	558.85(4)
Z	4	2
$\rho_{calc}g, cm^3$	1.405	1.683
μ, mm^{-1}	0.827	4.921
F(000)	800.0	284.0
Crystal size, mm^3	0.1 × 0.07 × 0.05	0.26 × 0.24 × 0.18
Radiation	CuKα (λ = 1.54184)	CuKα (λ = 1.54184)
2Θ range for data collection, °	5.576 to 138.18	7.744 to 138.824
Index ranges	$-9 \leq h \leq 9$<br>$-8 \leq k \leq 8$<br>$-35 \leq l \leq 38$	$-6 \leq h \leq 6$<br>$-10 \leq k \leq 10$<br>$-14 \leq l \leq 14$
Reflections collected	8926	4096
Independent reflections	3321<br>[R_{int} = 0.0249<br>R_{sigma} = 0.0333]	2066<br>[R_{int} = 0.0231<br>R_{sigma} = 0.0287]
Data/restraints/parameters	3321/0/255	2066/6/164
Goodness–of–fit on F^2	1.063	1.092
Final R indexes [I>=2σ (I)]	R_1 = 0.0502<br>wR_2 = 0.1432	R_1 = 0.0514<br>wR_2 = 0.1323
Final R indexes [all data]	R_1 = 0.0568<br>wR_2 = 0.1502	R_1 = 0.0526<br>wR_2 = 0.1336
Largest diff. peak/hole/ $eÅ^{-3}$	0.77/-0.78	1.17/-0.87
